# Ex-Situ and In-Situ Hybrid Processing Opportunities in Metal Additive Manufacturing

**DOI:** 10.3390/ma19183927

**Published:** 2026-09-15

**Authors:** Vishnu Ramasamy, Mingchen Qu, John J. Lewandowski

**Affiliations:** 1 Department of Materials Science and Engineering, Case Western Reserve University, Cleveland, OH 44106, USA; 2 Department of Materials Science and Engineering, The Ohio State University, Columbus, OH 43210, USA

**Keywords:** hybrid additive manufacturing, ex-situ hybrid processing, in-situ hybrid processing, directed energy deposition (DED), powder bed fusion (PBF), thermomechanical processing, heat treatment, recrystallization, porosity mitigation, microstructure control

## Abstract

Metal additive manufacturing (AM) enables near-net-shape fabrication of complex components, but process-induced defects and heterogeneities continue to limit performance and broader adoption. This review examines ex-situ and in-situ hybrid processing across multiple AM processes and alloy systems, including heat treatment, deformation-based processing, hot isostatic pressing, machining, friction stir processing, ultrasonic and magnetic-field assistance, alloying, stirring, and surface preparation. Ex-situ routes modify the completed build through bulk densification, homogenization, phase control, and surface improvement, whereas in-situ routes modify defects, microstructure, residual stress, and phase evolution during fabrication by interacting with the liquid melt pool, viscoplastic material, or solidified layer(s). The in-situ hybrid processing literature is dominated by wire-based directed energy deposition (DED) because its open architecture facilitates secondary-tool integration. Deformation-based methods were the most common and frequently promoted pore closure, recrystallization, texture modification, residual-stress redistribution, and improved mechanical performance. Hybrid processing was most effective when integrated to target the dominant material-specific limitation. Aluminum alloys benefited primarily from porosity control, stainless steels from grain and texture modification, titanium alloys from prior-β and α/β morphology control, nickel-based superalloys from segregation and Laves-phase mitigation, and steels from control of transformation microstructures. This material-state-aware framework may also extend to polymers and composites.

## 1. Introduction

**Figure 1:**
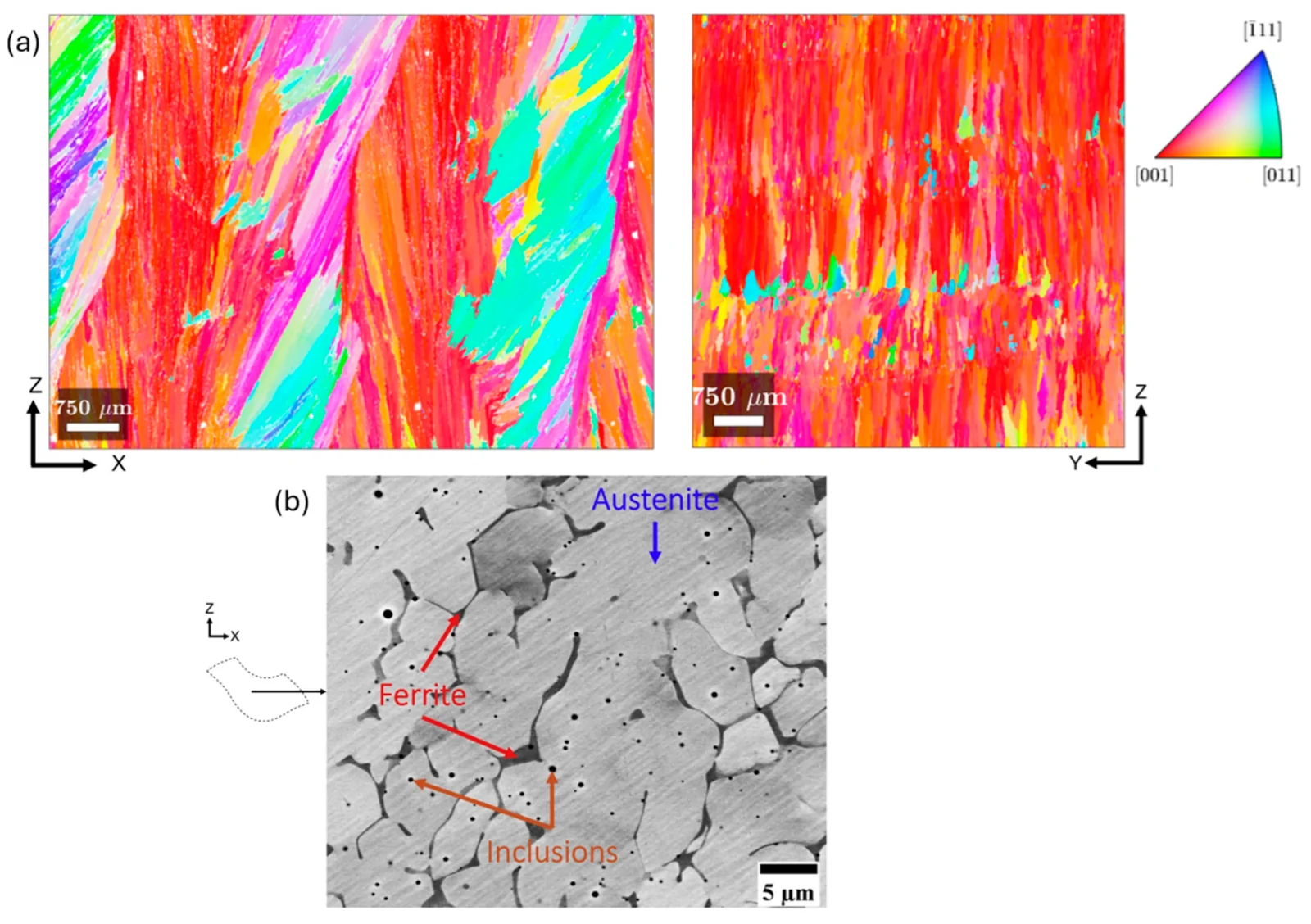
Wire DED 316L (**a**) Electron Backscatter Diffraction (EBSD) orientation maps showing coarse, directional columnar grains with strong <100> texture in XZ and YZ planes (**b**) Scanning Electron Microscopy (SEM) image showing microstructural segregation (Ferrite—red arrows), inclusions, and pores distributed within the γ-austenite matrix [7]—reproduced with modifications under Creative Commons (CC BY) license.

**Figure 2:**
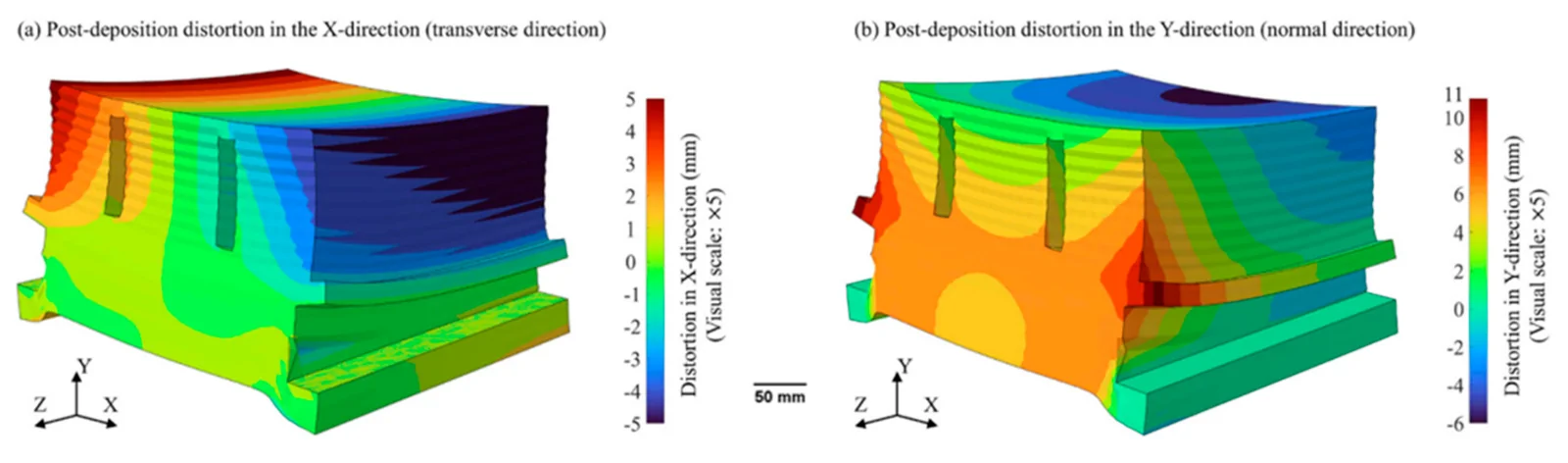
Distortion experienced in large-scale wire DED of Ti-6Al-4V at the end of deposition in (**a**) X-direction and (**b**) Y-direction, after cooling down to ambient temperature (Note: Distortion is visually exaggerated ×5 to assist perception) [6]—reproduced under Creative Commons (CC BY NC ND) license.

**Figure 3:**
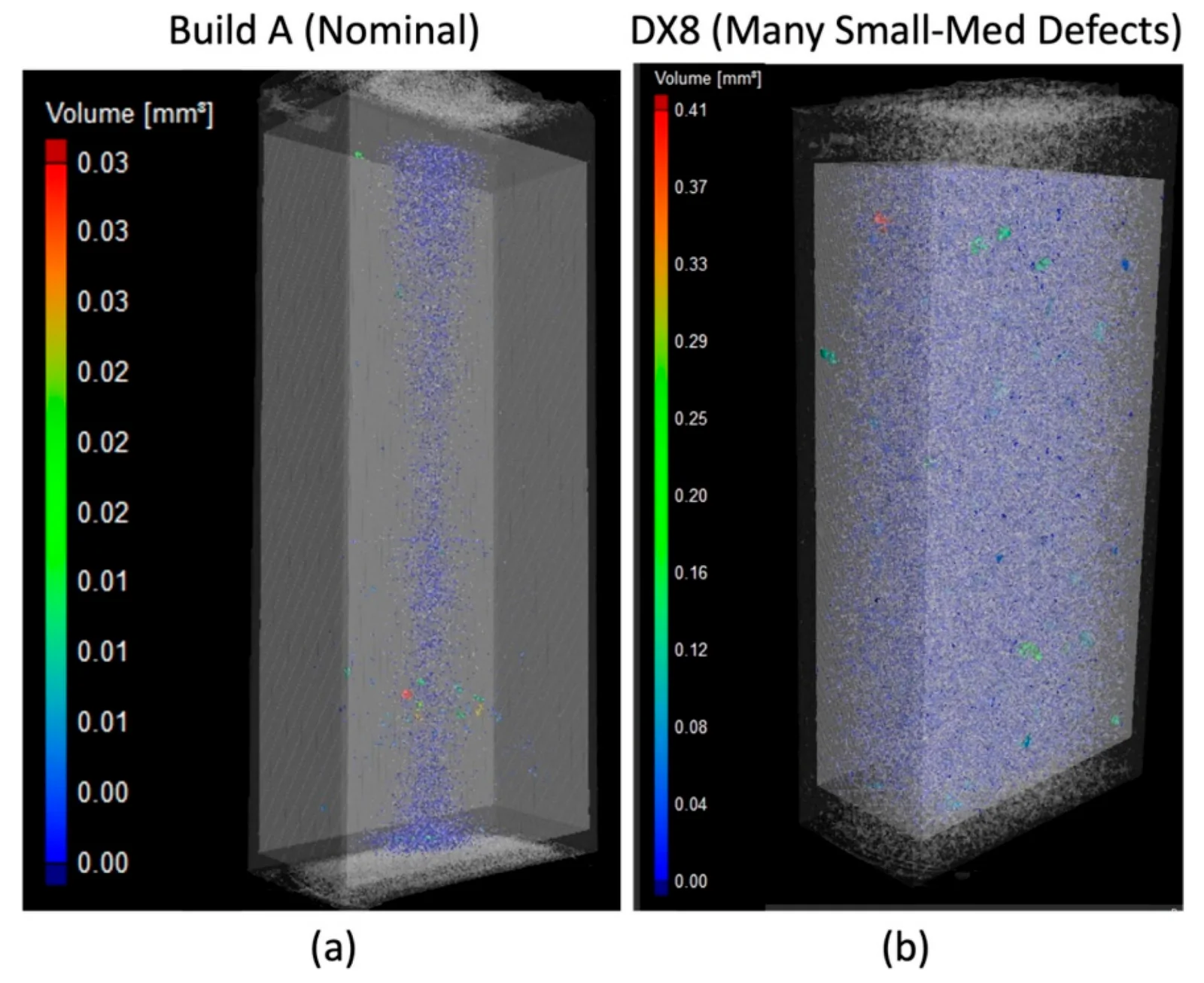
X-Ray Computed Tomography (XCT) scans showing porosity distribution in PBF AlSi10Mg with (**a**) optimal parameters resulting in relative density of 99.6% and (**b**) sub-optimal parameters resulting in relative density of 92.2% [8]—reproduced under Creative Commons (CC BY NC ND) license.

Metal additive manufacturing (AM) has transformed modern manufacturing by enabling the fabrication of complex, near-net-shape components with high material efficiency. Among AM techniques, powder bed fusion (PBF) and directed energy deposition (DED) are two of the most commercially relevant process families [1,2], because of their ability to fabricate high-performance components while reducing material waste and lead time for high-value, high-mix applications in aerospace, automotive, biomedical, and energy sectors [3,4]. Despite these advantages, the layer-by-layer nature of AM imposes complex thermal histories characterized by rapid solidification, steep thermal gradients, and repeated cyclic reheating. These conditions are widely reported to promote undesirable features, including large directional columnar grains, porosity, elemental segregation, residual-stress-induced distortion, and spatially heterogeneous microstructures [5,6,7,8], as shown in Figure 1 [7], Figure 2 [6], and Figure 3 [8]. Such features are associated with pronounced microstructural and mechanical anisotropy [9,10]. Consequently, as-built AM metallic components have often exhibited mechanical property scatter and reduced performance consistency, creating challenges for qualification and certification in critical structural applications [11,12]. The principal AM-induced defects and microstructural heterogeneities with typical consequences are summarized in Table 1.

**Figure 4:**
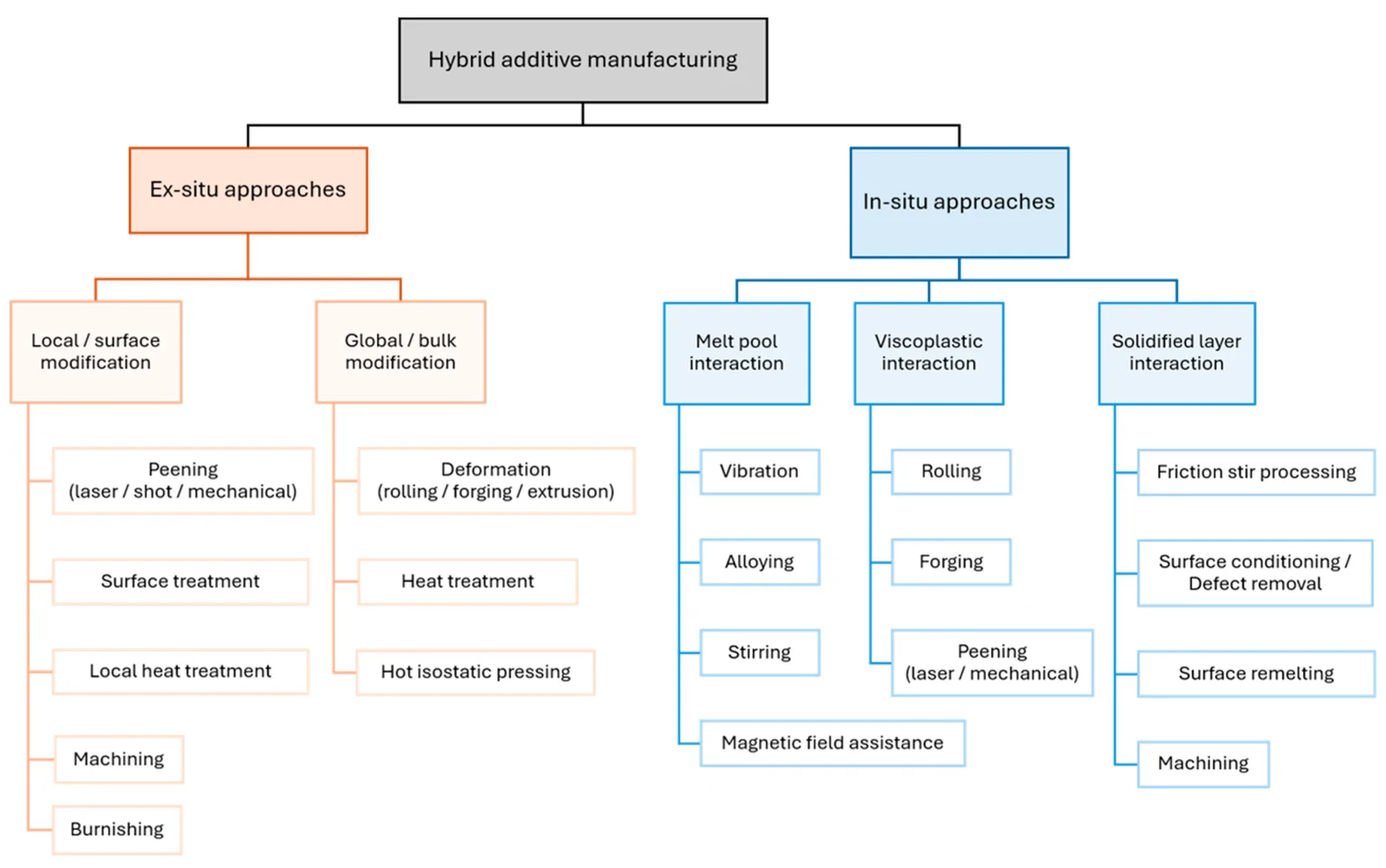
Conceptual classification of hybrid additive manufacturing approaches based on in-situ material-state interactions and ex-situ post-processing routes.

Hybrid manufacturing provides a pathway to overcome the limitations of individual manufacturing processes by combining two or more processes, tools, or energy sources within a unified manufacturing workflow. Classic descriptions of hybrid manufacturing emphasize that such combinations may involve additive, subtractive, transformative, joining, or dividing operations, and that the benefit of hybridization depends on the processes being combined, as well as their timing [13,14]. In metal AM, this concept is particularly relevant because the deposition process can be coupled with one or more secondary processes to overcome inherent limitations. Throughout this review, hybrid processing will be described using a primary/secondary process framework. The primary process refers to the metal AM deposition itself, whereas the secondary process is the supplemental operation combined with the primary process to enhance the final material response. These secondary processes may include deformation, thermal treatment, magnetic-field assistance, surface preparation, alloying, or the addition of reinforcements, that are intended to modify, locally or globally, one or more aspects of geometry, surface condition, or evolving material state, including microstructure, defect population, residual-stress distribution, and phase evolution, with the broader goal of improving mechanical, corrosion, or other service-relevant performance. Hybrid AM can be broadly classified into ex-situ or in-situ approaches. Ex-situ hybrid approaches apply standalone secondary processing after the primary AM fabrication sequence is complete, whereas in-situ hybrid approaches integrate secondary processing with primary AM, including synchronously with the deposition, between deposited layers, or immediately after deposition but within the fabrication sequence. The effectiveness of either approach depends strongly on the selected processing window, and improperly controlled secondary processing can introduce or reintroduce undesirable features such as pore reopening during subsequent heat treatment, grain coarsening, unwanted phase precipitation, loss of deformation history through remelting, or undesirably non-uniform spatial modification. A conceptual classification of ex-situ and in-situ hybrid AM approaches is shown in Figure 4.

This review first summarizes representative ex-situ processing approaches to establish a conventional post-processing baseline for metal AM. It then focuses on in-situ hybrid processing, emphasizing how secondary processes interact with the evolving thermal, mechanical, and microstructural states of the material during deposition. Across multiple alloy systems, the review summarizes how these approaches address dominant material-specific limitations, including porosity, columnar grain growth, elemental segregation, and undesirable phase formation. By comparing the resulting microstructural evolution, governing mechanisms, and property changes, the review provides a structured assessment of current hybrid-processing strategies and identifies key scientific and technological gaps that must be overcome to enable broader industrial adoption of such hybrid approaches. Details of the literature-search strategy, study-selection criteria, and quantitative classification procedures are provided in Appendix A.

## 2. Ex-Situ Hybrid Processing

Conventional ex-situ processing remains an established route for modifying the microstructure and improving the performance of metal AM components. In these workflows, AM fabricates near-net-shape components or intermediate preforms, which are then subjected to separate thermal, mechanical, thermomechanical, densification, or surface-finishing operations [15,16]. Representative ex-situ studies reported since the early 2000s are summarized in Table 2.

**Figure 5:**
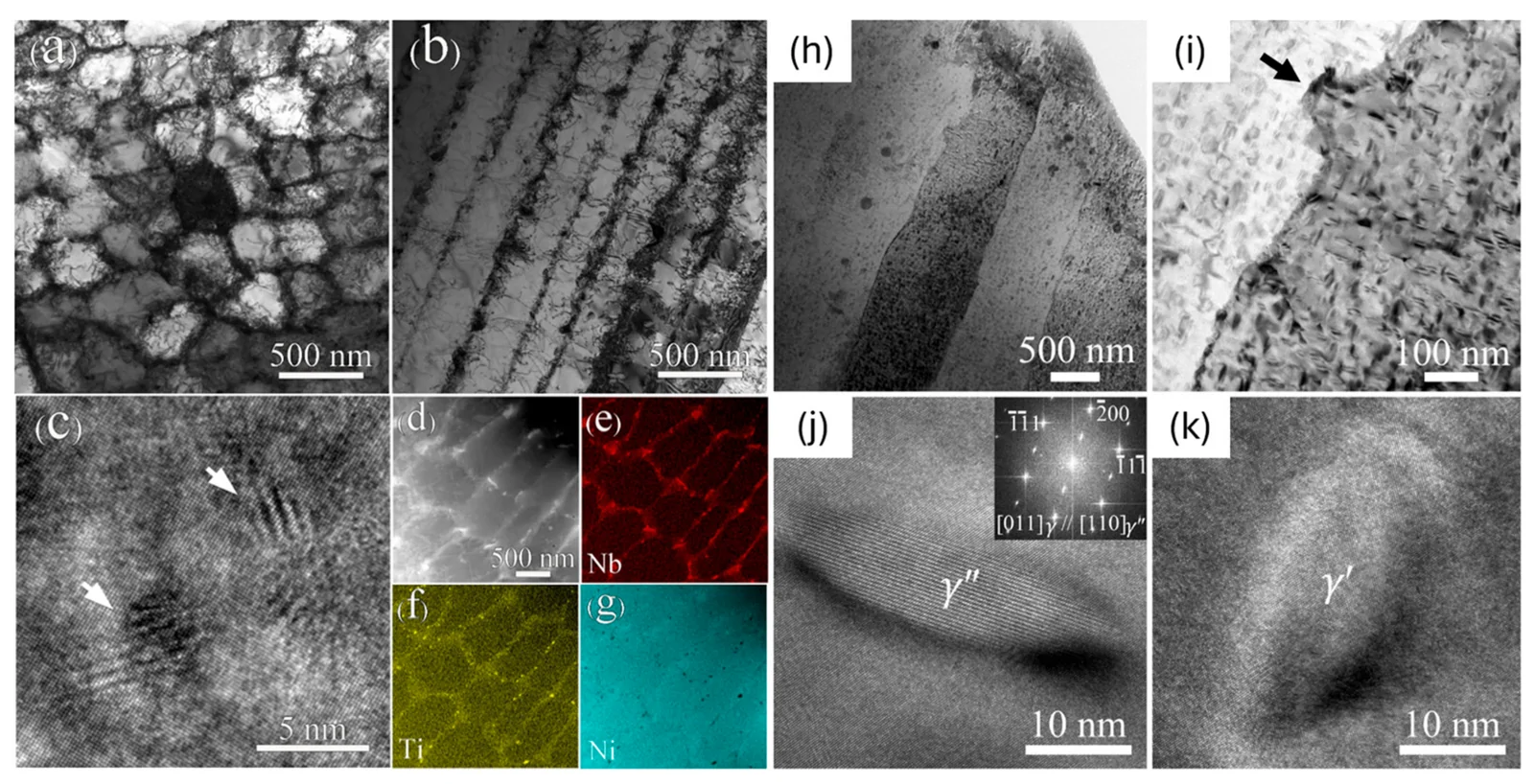
(**a**,**b**) Bright-field micrographs of as-deposited PBF IN718 showing formation of columnar dislocation cells; (**c**) high-resolution transmission electron microscopy (TEM) images of nanoprecipitates observed within the dislocation cells; (**d**) high-angle annular dark-field scanning transmission electron microscopy image; (**e**–**g**) corresponding energy dispersive X-ray spectroscopy maps highlighting Nb, Ti, and Ni distribution; (**h**) Bright-field micrograph of PBF IN718 in AM-specific heat treated condition showing dislocation cell network with diffraction contrast from dense nanoprecipitation; (**i**) micrograph showing the presence of nanoprecipitation and Zener pinning of the intercellular boundary by MC carbide particle; high-resolution TEM micrograph taken along [011]γ showing formation of (**j**) γ″ and (**k**) γ′ nanoprecipitates throughout the γ matrix [18]—reproduced with modifications under Creative Commons (CC BY) license.

Heat Treatment (HT) is among the most widely used ex-situ approaches because it is simple yet effective for modifying phase constitution, relieving residual stress, homogenizing segregation, and optimizing strength-ductility balance with minimal geometric change. Its effectiveness strongly depends on alloy chemistry, initial AM microstructure, and the selected thermal path. For instance, in one PBF Ti-6Al-4V study [17], a sub-transus heat treatment at 850 °C for 2 h, followed by furnace cooling, decomposed the as-built α′ martensite into α + β while largely retaining the columnar prior-β structure. This increased elongation from 7.28 ± 1.12% to 12.84 ± 1.36%, although yield strength (YS) and ultimate tensile strength (UTS) decreased by 14% and 21%, respectively, due to a proposed reduction in interface strength and work-hardening capacity. With super-transus treatment at 1020 °C for 2 h followed by furnace cooling, elongation increased only to 14.06 ± 2.53%, but prior-β grains became substantially coarser, and YS and UTS fell by 31.5% and 33.7%, respectively, compared with as-deposited conditions [17]. Similarly, in PBF IN718 [18], the AM microstructure contained dislocation cells, Nb/Ti segregation, Laves phase, and aging-responsive γ′/γ″ precipitation. An AM-specific heat treatment using a short solution anneal above the δ-solvus (1020 °C for 15 min), followed by water quenching and aging at 720 °C for 24 h, dissolved the Laves phase and suppressed δ formation, while retaining the AM-induced dislocation-cell network and promoting dense γ′/γ″ nanoprecipitation (Figure 5) [18]. This achieved a YS of 1245 MPa, a UTS of 1640 MPa, and an elongation of 16.6%. Compared with the conventional heat treatment route, this represented increases of 0.4% in YS, 5.1% in UTS, and 43.1% in elongation [18]. A similar microstructural response was reported in binder-jet IN625 [19]. Solutionizing the sintered component at 1150 °C for 2 h dissolved pre-existing precipitates and redistributed solute while increasing elongation from 40.9 ± 3.1% to 45.1 ± 3.3%. However, subsequent aging at 745 °C reintroduced γ″, carbide, and TCP/Laves-type phases, reducing elongation to 30.1 ± 6.7% [19].

**Figure 6:**
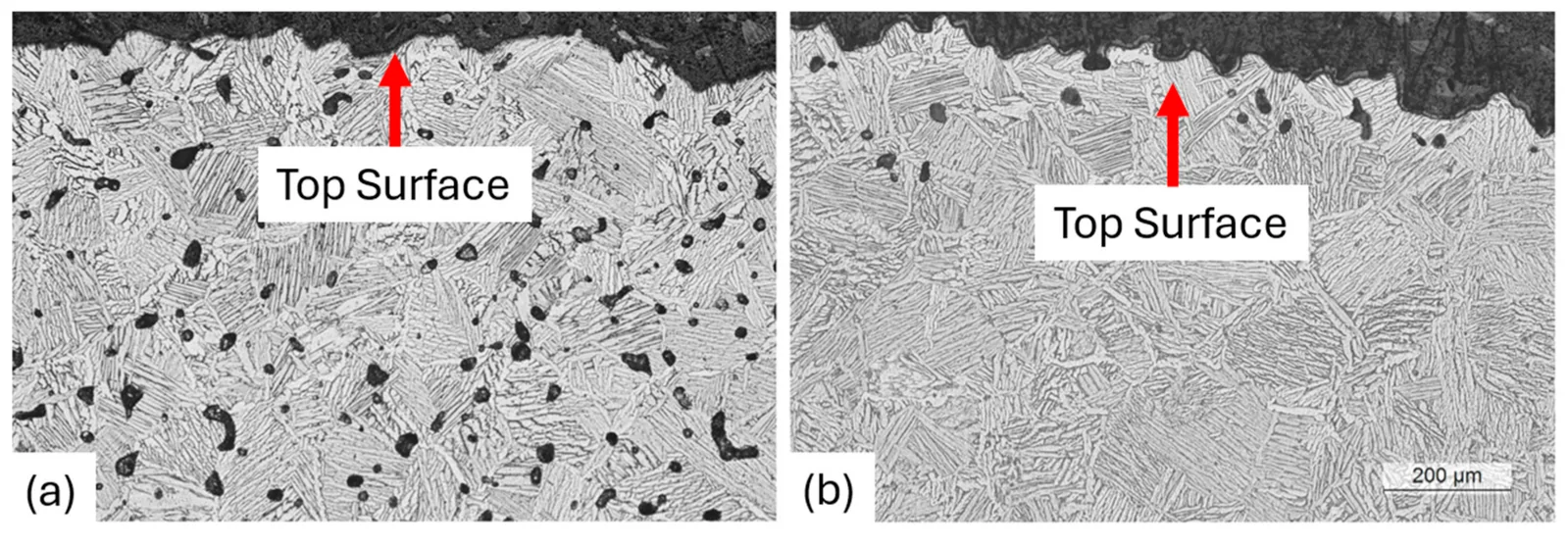
Cross-sectional micrographs of binder jet Ti-6Al-4V show porosity (presumably connected to the outside surface) near the top surface of (**a**) as-sintered and (**b**) after HIP at 850 °C for 2 h [22]—reproduced with modifications under Creative Commons (CC BY) license.

Hot Isostatic Pressing (HIP) is used as an ex-situ densification route, especially for reducing residual porosity, in powder metallurgy and metal AM. The effectiveness of HIP in controlling porosity in metal AM strongly depends on defect morphology, particularly whether pores are isolated, surface-connected, or contain entrapped gas that may be insoluble in the alloy (e.g., Ar in Ti alloys). This distinction is especially important in material extrusion (ME) and binder jetting (BJ), where consolidation is inherent to the process chain and substantial residual porosity can remain after debinding and sintering. In these processes, the printed green part consists of metal powder held together by a polymer blend or binder and must undergo debinding and/or sintering to form a fully metallic component [36,37]. In BJ Ti-6Al-4V, HIP sealed nearly all internal pores and channels formed during sintering, but surface-connected defects persisted because pressurized gas could enter open voids during HIP, blocking pore closure (Figure 6) [22], as also demonstrated in PBF AlSi10Mg study [8]. While the as-sintered BJ Ti-6Al-4V material exhibited a YS of 790 MPa, UTS of 912 MPa, and elongation of 9.35%, HIP at 850 °C/200 MPa for 2 h increased these values to 1002 MPa, 1089 MPa, and 10.58%, respectively, but the elongation remained well below the wrought reference value of 19.5% [22]. The limited ductility and fatigue recovery were attributed to residual porosity and α-lath coarsening, in addition to ~0.35 wt.% oxygen content and oxygen-rich Ti dispersoids associated with the binder/sintering route.

**Figure 7:**
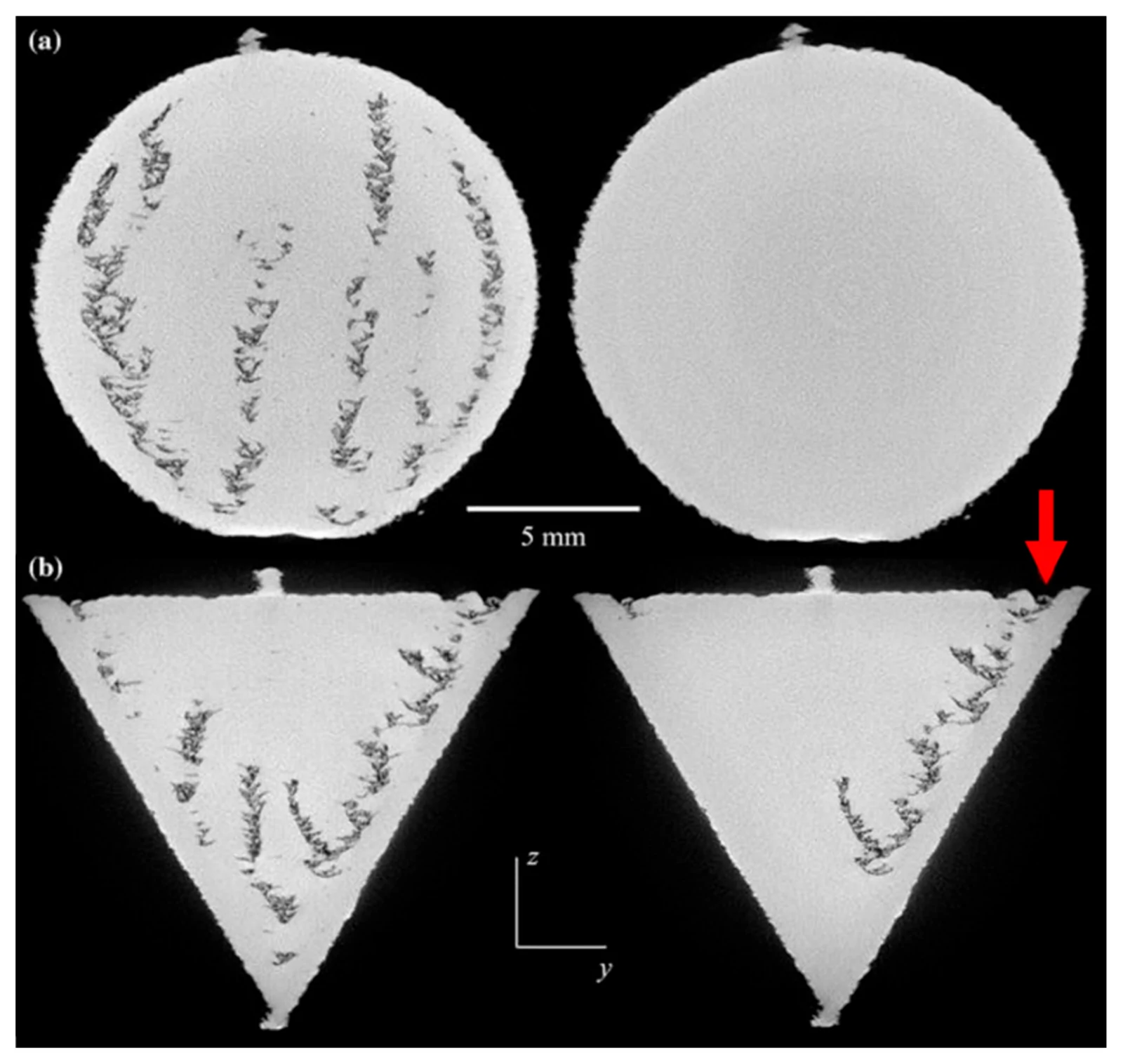
Slices of PBF Ti-6Al-4V from X-ray computed tomography data collected as-built (left) and after HIPing (right) in (**a**) cylindrical and (**b**) triangular prism samples. Red arrow in (**b**) shows a tunnel-like surface-connected defect present after HIPing. The build direction for both samples is along *z*-axis [20]—reproduced with modifications under Creative Commons (CC BY) license.

**Figure 8:**
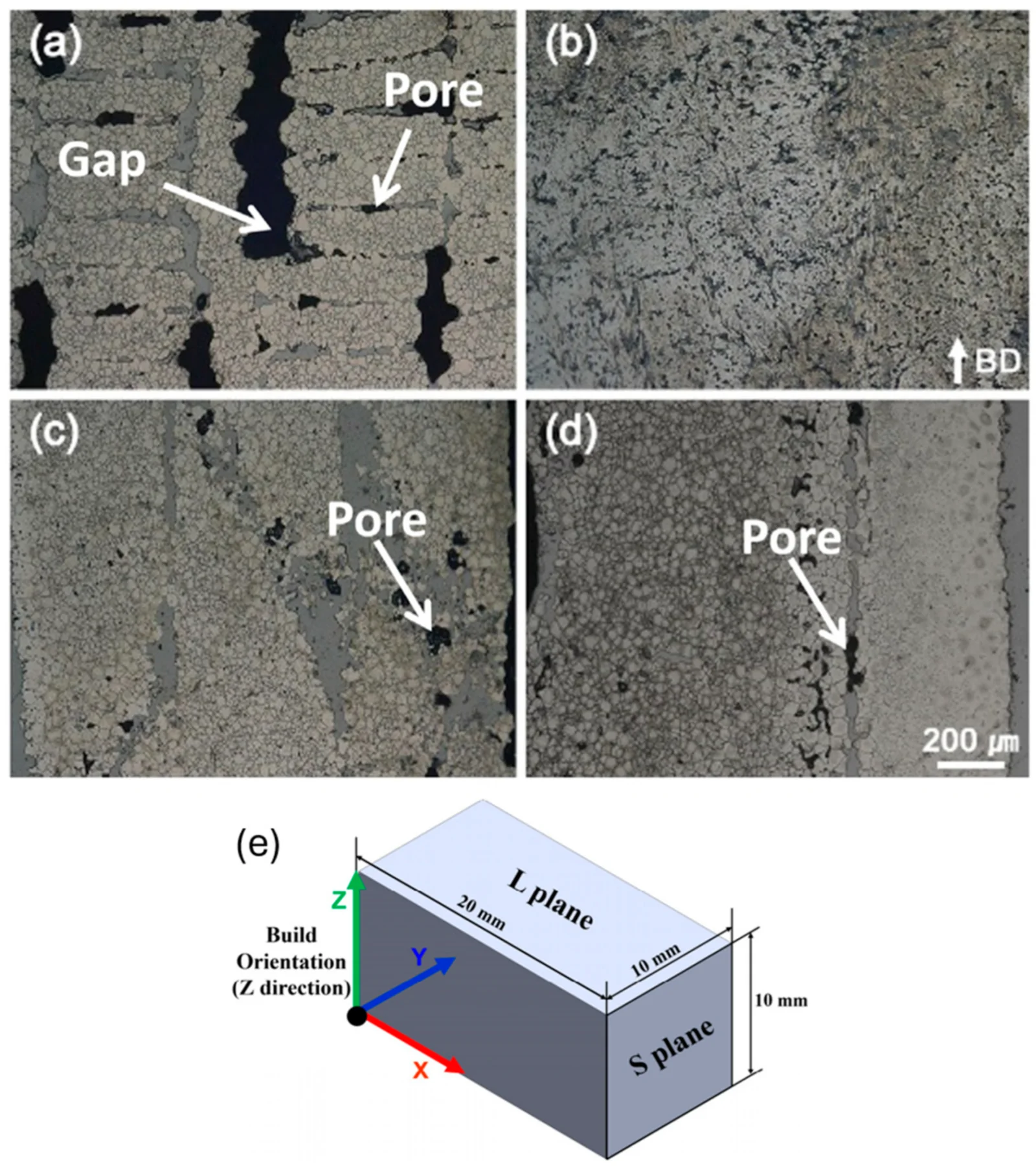
Optical micrographs of material extrusion 316L specimens at (**a**) S plane without warm isostatic pressing (WIP), (**b**) S plane with WIP, (**c**) L plane without WIP, (**d**) L plane with WIP, and (**e**) illustration of S and L planes [27]—reproduced with modifications under Creative Commons (CC BY) license.

Similarly, in PBF Ti-6Al-4V, standard HIP at 920 °C/100 MPa for 2 h reduced internal porosity below the ~5.2 µm resolution limit, with 100% pore-volume reduction in several geometries. However, as shown in Figure 7, surface-connected tunnel defects persisted because HIP gas entered the open voids [20] and prevented their closure. In a 316L ME study, warm isostatic pressing (WIP) was applied to the green part before debinding and sintering, which increased the tensile strength by ~2.5x and the strain at UTS by ~4x compared with the non-WIP condition. Although WIP did not eliminate all pores, the study attributed the performance improvement to a 55.1% increase in metal-powder area fraction in the green part, and density increases of 8.7% in the green part and 7.8% in the final sintered part, and a resulting reduction in inter-strand gaps (Figure 8) [27].

**Figure 9:**
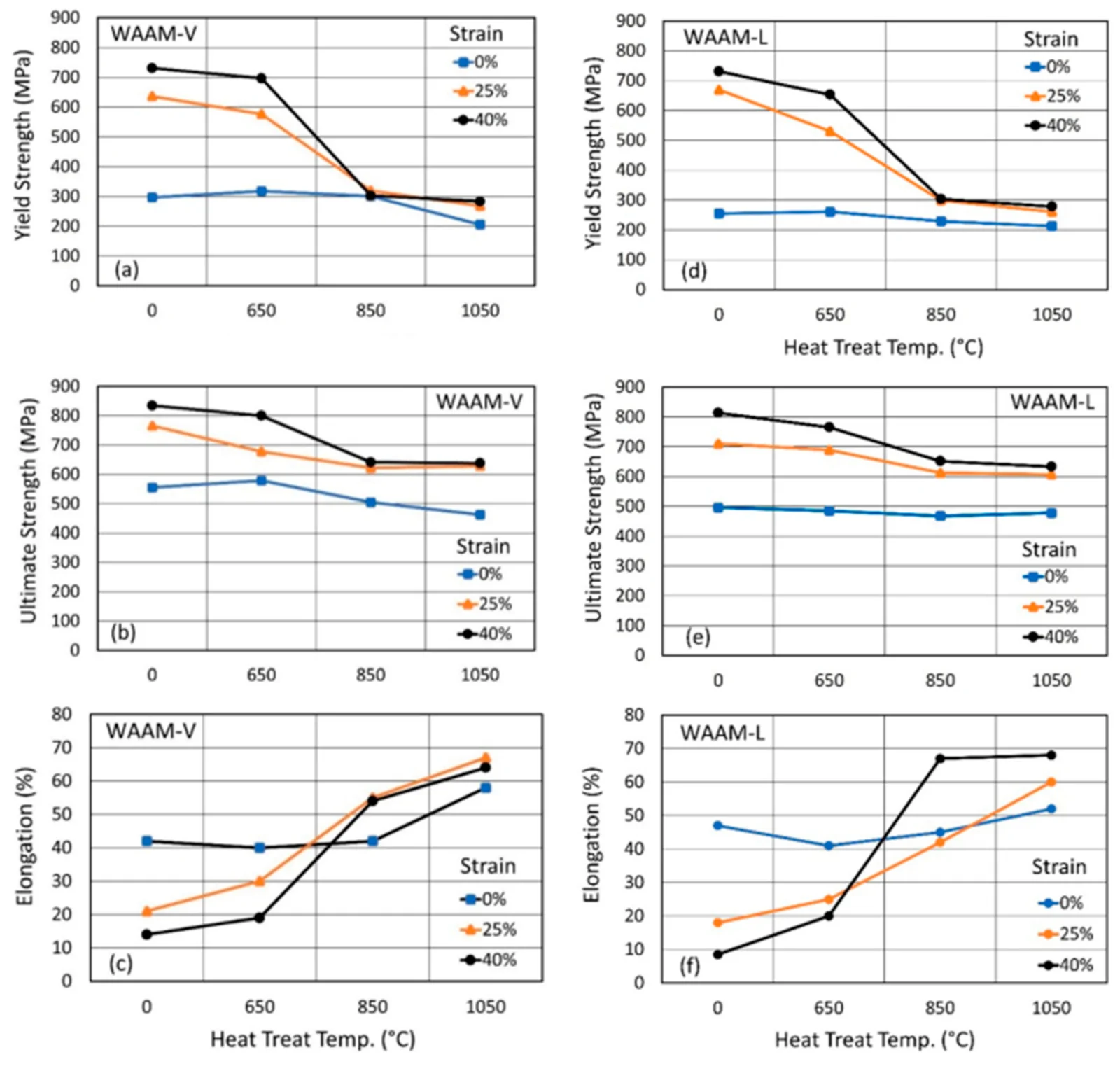
Wire DED 304L showing variations in (**a**,**d**) Yield Strength (**b**,**e**) Ultimate Tensile Strength and (**c**,**f**) Elongation along vertical-V and longitudinal-L directions, at various temperatures, before and after ex-situ thermomechanical processing using rolling and heat treatment. (**a**) to (**f**) show the sample rolling strain ID that was converted to both engineering and true strain [31]—reproduced under Creative Commons (CC BY) license.

Thermomechanical processing provides an aggressive ex-situ route by imposing plastic deformation that disrupts the AM grain structure and stores strain energy, followed by thermal exposure that activates recovery and recrystallization. This combined action can accelerate globularization, weaken texture, and reduce microstructural and property anisotropy [38]. In PBF and DED produced Ti-6Al-4V, the initially fine α features were ~0.54 and 0.94 µm, respectively, compared with 2 µm in wrought material. After compression at 950 °C, 0.01 s^−1^, and a strain of 0.9, the globularized fraction reached ~90% in PBF material and 98% in DED material, compared with only 54% in wrought Ti-6Al-4V, while peak flow stresses were reduced by up to 18–20% [28]. Another DED Ti-6Al-4V study also reported substantial modification to the as-deposited microstructure by forging at ~930 °C to 50% reduction [30]. Subsequent stress-relief annealing at 710 °C for 6 h produced partially globularized α grains of ~2.3 µm with about 9% β phase and largely removed the characteristic DED morphology. The resulting tensile properties were nearly orientation-independent, with UTS of 912–916 MPa, YS of 838–844 MPa, and elongation of 13.1–14.5% [30]. Another substantial modification to the as-deposited microstructure was reported in a wire DED 304L study [31], where heat treatment without prior deformation failed to eliminate the columnar structure, texture, and elastic anisotropy. Warm rolling to 24.9% or 41.1% true strain (engineering strains of 22.6% or 35.4%) followed by 850–1050 °C heat treatment reduced texture and elastic anisotropy [31]. As shown in Figure 9, after 41.1% true strain (sample rolling strain ID = 40% in Figure 9 and engineering strain of 35.4% converted to true strain of 41.1%) and recrystallization at 850 °C, vertical and longitudinal moduli converged to 190–193 GPa, UTS to 641–652 MPa and elongation to 54–67%, indicating near-elimination of the orientation-dependent response [31].

**Figure 10:**
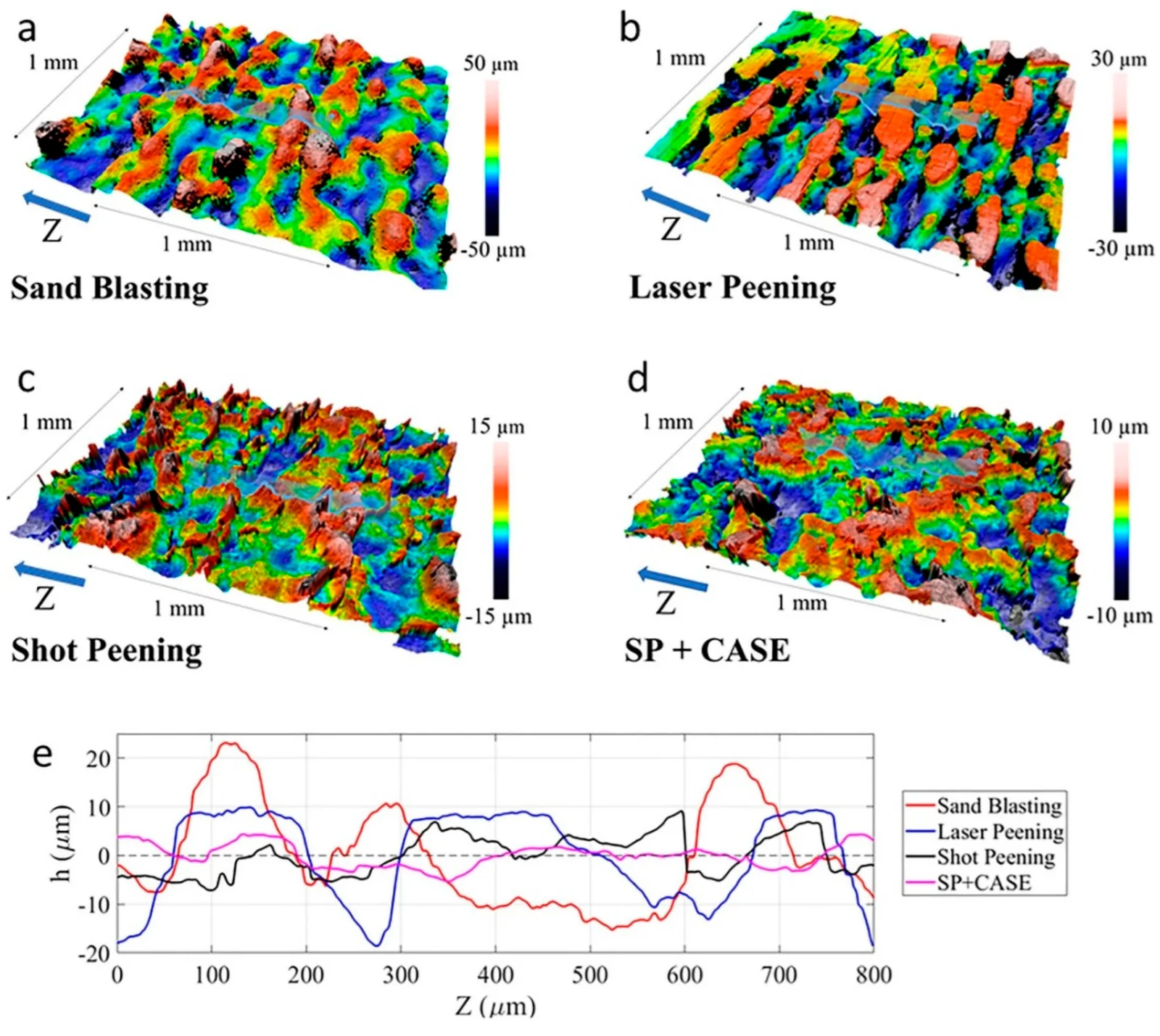
3D profiles of the surface roughness for the four surface treatments of PBF Ti-6Al-4V: (**a**) Sand Blasted baseline (SB), (**b**) Laser Peened (LP), (**c**) Shot Peened (SP), and (**d**) SP + chemically assisted surface enhancement (CASE), (**e**) Line roughness profiles along the blue lines marked in mid-surfaces of (**a**) to (**d**) [32]—reproduced under Creative Commons (CC BY) license.

In addition to bulk modification approaches, localized methods such as peening and machining operations target surface-level limitations, including roughness, near-surface defects, and residual stresses. In PBF Ti-6Al-4V [33], mechanical surface finishing by milling, blasting, vibratory grinding, and micromachining was reported to substantially reduce roughness and improve fatigue performance. In this study, Ti-6Al-4V components were stress annealed and HIPed before surface-finishing. The HIPed condition had an initial roughness of Ra = 17.9 µm and produced poor fatigue performance, with a run-out stress of 300 MPa at 3 × 10^7^ cycles. Surface finishing reduced roughness, with milling reaching Ra = 0.3 µm and increasing the run-out stress to 775 MPa at 3 × 10^7^ cycles [33]. In another study of PBF Ti-6Al-4V [32], laser peening (LP), shot peening (SP), and SP + chemically assisted surface enhancement (CASE) were compared after a common sandblasted and annealed baseline. This study also targeted fatigue life improvement, but by introducing near-surface compressive residual stresses using peening. As shown in Figure 10 [32], SP reduced roughness from Ra = 11.7 to 4.6 µm, SP + CASE further reduced it to 2.7 µm, and LP retained relatively high roughness of 10.2 µm but produced a deeper compressive residual-stress field with peak compression near −600 MPa. Improvements were reported in all three treatments, with SP increasing fatigue strength by ~35–75%, SP + CASE increasing fatigue life by nearly 10 times relative to SP near 10^6^ cycles, and LP giving the best overall response. This ranking reflected the combined effects of roughness, compressive-stress depth, and crack-initiation location, with internal crack initiation observed in all SP + CASE failures, four of six LP failures, and only one SP failure [32].

**Figure 11:**
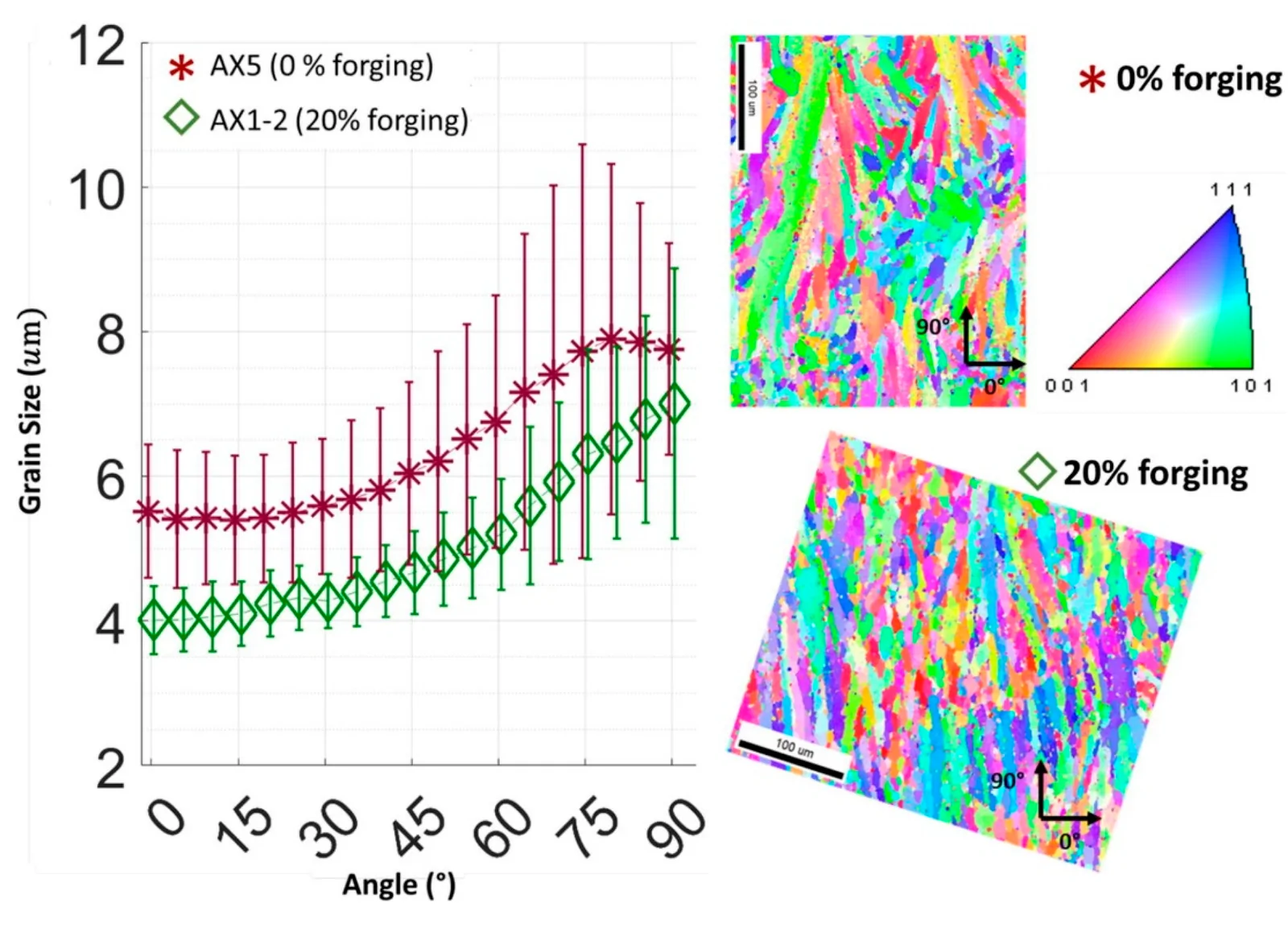
EBSD mapping and measurement of average grain size in nominal build PBF AlSi10Mg specimens under 0% and 20% forging reduction conditions, with changes in grain size relative to map orientation indicating grain anisotropy [8]—reproduced under Creative Commons (CC BY NC ND) license.

Advanced ex-situ routes combine multiple secondary processes for better microstructural control. For instance, in PBF Ti-6Al-4V [23], a super-β-transus + temper HIP route, 1050 °C/100 MPa for 2 h followed by rapid cooling and tempering at 800 °C/30 MPa for 2 h, reduced as-built porosity from 0.21 vol.% to below the XCT detection limit of ~3 µm. Unlike conventional sub-transus HIP, which coarsened α laths from 1.16 ± 0.26 to 2.17 ± 0.51 µm and reduced strength, this route retained fine α laths, 1.20 ± 0.32 µm, produced equiaxed prior-β grains, and maintained YS and UTS values comparable to the as-built condition at 885 ± 6 and 985 ± 12 MPa, respectively [23]. In the PBF AlSi10Mg study, isothermal forging was combined with stress relief HIP and T6 heat treatment for maximum benefit [8]. Baseline deposition conditions included a nominal build produced with 99.6% relative density and a defect-rich build with 92.2% relative density (Figure 3). The baseline heat treatment included stress relief at 285 °C for 120 min, HIP at 510 °C/102 MPa for 135 min, T6 solutionizing at 530 °C for 360 min, and aging at 160 °C for 360 min for both conditions. Forging was then performed at 530 °C after a 1 h hold, using 20% height reduction at 10^−3^ s^−1^, followed by water quenching and repeat T6 treatment. The defect-rich build contained lack-of-fusion defects hundreds of micrometers in size and showed a ~30% lower UTS than the nominal build, with failure occurring before measurable 0.2% yield strength. After 20% forging, defect area fraction decreased by ~50% in both builds; the reduction in area increased from 7% to 15% in the nominal material and from 0.7% to 5% in the defect-rich material, and the grain structure changed from typical AM morphology to smaller, more equiaxed grains, as shown in Figure 11 [8]. However, lack-of-fusion defects were reshaped into more ellipsoidal features rather than fully eliminated, indicating that moderate global deformation may improve ductility, grain morphology, and defect shape, but may not fully close severe process-induced defects at this lower forging strain [8]. Increasing the amount of forging reduction in such studies may continue to reduce the void density and change microstructure features with resulting improvements in various mechanical properties.

Beyond such sequentially integrated treatments, some studies combined processes that targeted different defect populations. For instance, in PBF GRX-810 [34], machining and HIP improved fatigue resistance through complementary surface and volumetric-defect control mechanisms. At a strain amplitude of 0.002 mm/mm, fatigue life increased from ~6 × 10^4^ cycles in the unmachined non-HIP condition to ~2–4 × 10^5^ cycles with either machining or HIP alone, and to ~1.8–2.4 × 10^6^ cycles with both. Machining removed surface micro-notches and near-surface defects, while HIP reduced volumetric defects and promoted intragranular (Nb,Ti)C carbides that impeded persistent slip-band formation [34].

These representative studies show that ex-situ processing can improve the microstructure and performance of metal AM components, while enabling the use of AM to create near net shape preforms that can then be subsequently post-processed. However, ex-situ treatments are applied only after the AM thermal history, defect population, residual stress state, and solidification microstructure have already developed. This route creates two important limitations. First, ex-situ processing can restrict AM-fabricated components to geometries compatible with conventional forming, machining, HIP, or surface-finishing tools, dies, and operations, thereby reducing the design freedom inherent to AM [15,28,30]. Second, reliance on large-scale industrial equipment, such as forging machines, HIP vessels, furnaces, and machining systems, along with additional handling and logistics, can increase energy consumption, cost, and lead time. A case-specific comparative study highlighted this potential inefficiency by reporting that the conventional wrought Ti-6Al-4V production of a demonstrative component required a specific energy consumption of ~3.32 × 10^5^ kJ/kg, whereas fabrication of the same component using an in-situ deformation-assisted DED process with comparable mechanical properties required ~5.31 × 10^4^ kJ/kg [39]. Although these values are strongly dependent on geometry, process route, and production scale, they highlight the potential of integrating secondary processes directly into AM to achieve efficient microstructural control during primary solidification, rather than relying solely on downstream post-processing. Unlike ex-situ methods, the in-situ hybrid approaches reviewed next can use the intrinsic thermal and mechanical conditions of solidifying material as processing opportunities rather than treating them as sources of defects.

## 3. In-Situ Hybrid Processing

Metal AM includes several process families [40], but their suitability for in-situ hybrid processing differs substantially because the timing of metallurgical consolidation, process accessibility, and available tool space vary widely across platforms. In binder jetting (BJ) and material extrusion (ME), as discussed earlier, the printed part is typically a green or partially consolidated body, and full metallurgical bonding is achieved later through debinding, sintering, and, in some cases, additional densification [36,37]. As a result, secondary intervention during the primary printing process has limited opportunity to directly modify solidification microstructure, residual stress, or metallurgically bonded defect structures. For these process families, ex-situ thermomechanical or HIP treatments, reviewed above, remain the more relevant routes for controlling final material performance.

Powder bed fusion (PBF), unlike BJ and ME, produces direct metallurgical consolidation during the primary AM deposition. However, the powder bed, recoating mechanism, enclosed chamber, inert atmosphere, and restricted build volume strongly limit the integration of external secondary tooling. Consequently, most in-situ strategies explored in PBF have relied on interventions that are compatible with optical access suitable for the limited chamber space, such as laser reheating, remelting, or localized surface modification. For instance, a dual-laser approach using nanosecond pulsed shock-wave cleaning removed up to 80.9% of loose powder from inclined maraging-steel surfaces, enabling subsequent continuous-wave laser remelting to reduce surface roughness, Ra, by 55%, from 15 to 6.8 µm [41]. Similarly, in-situ laser reheating in PBF stainless steels, including 2507 and 316L, reduced the fraction of very high-angle grain boundaries greater than 45° through dynamic recovery, while grain size and texture remained largely unchanged [42]. These studies demonstrate that PBF can benefit from targeted in-situ modification, but the accessible processing window is generally constrained to thermal, optical, or surface-level interventions.

Directed energy deposition (DED) is particularly well suited for in-situ hybrid processing because its process architecture provides substantially greater access to the evolving deposit. In wire DED, robotic or CNC platforms often operate in open or semi-open configurations, allowing secondary tools to be positioned near the deposition zone or applied between deposited layers. Powder DED may still require enclosed chambers for environmental control, but the absence of a powder bed and recoating system provides greater spatial freedom than PBF. These architectural advantages have encouraged the exploration of a broad range of in-situ hybrid strategies in DED systems over the past decade. Reported secondary processes include deformation-based processing such as forging, rolling, and peening, as well as ultrasonic assistance, friction stirring, magnetic-field assistance, interlayer surface preparation, alloying, reinforcement addition, and localized heat treatment. Accordingly, the remainder of this review focuses on in-situ hybrid DED studies, where secondary processes interacted directly with the evolving thermal, mechanical, and microstructural state of the material during fabrication.

### 3.1. Classification of Secondary Processes

Table 3 summarizes the reviewed in-situ hybrid DED approaches according to secondary processing category and provides details regarding the DED type, recrystallization (RX) response, and material systems explored in each category.

**Figure 12:**
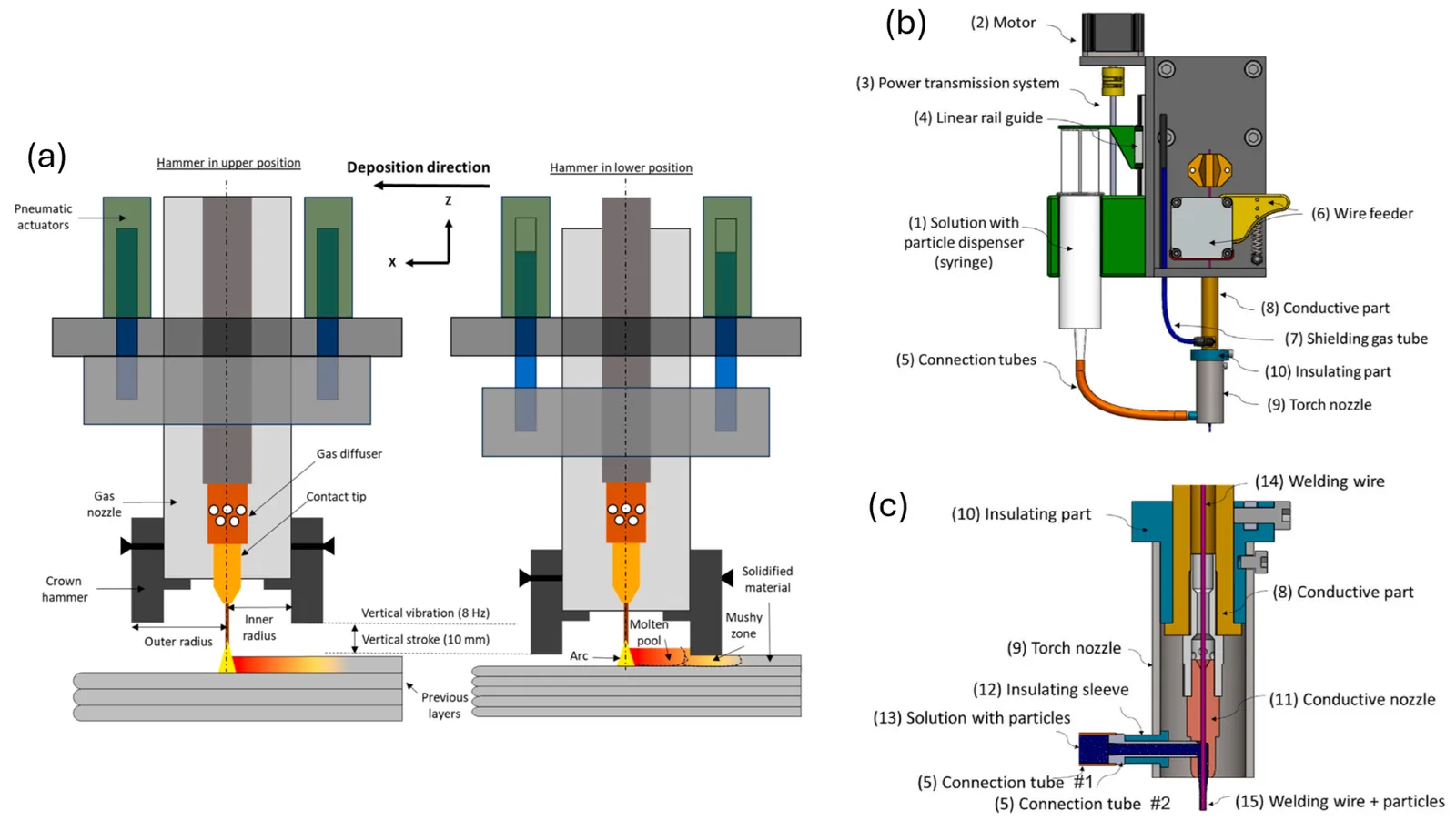
(**a**) Schematic of wire DED and in-situ hot forging [43]; Schematic of wire DED and in-situ alloying setup showing (**b**) full assembly and (**c**) cross-sectional view of the welding torch customized for in-situ alloying [98]—reproduced with modifications under Creative Commons (CC BY) license.

Representative in-situ hybrid DED systems are discussed here to illustrate the breadth of possible secondary process integration. In a wire DED study on Haynes 282 [43], the welding torch was coupled with a pneumatic forging unit positioned close to the deposition zone. A crowned hammer mounted to that forging unit impacted the bead shortly after deposition while the material remained in a viscoplastic state, as illustrated in Figure 12a [43]. Under the reported forging conditions, this approach modified microstructural texture, reduced porosity, refined grains, and improved selected mechanical properties. Figure 12b,c show another wire DED system [98], in which TiB_2_ particles were introduced into the molten pool during IN625 deposition. The added particles promoted heterogeneous grain nucleation and restricted dendritic growth, resulting in a finer, more equiaxed microstructure with reduced interdendritic segregation.

**Figure 13:**
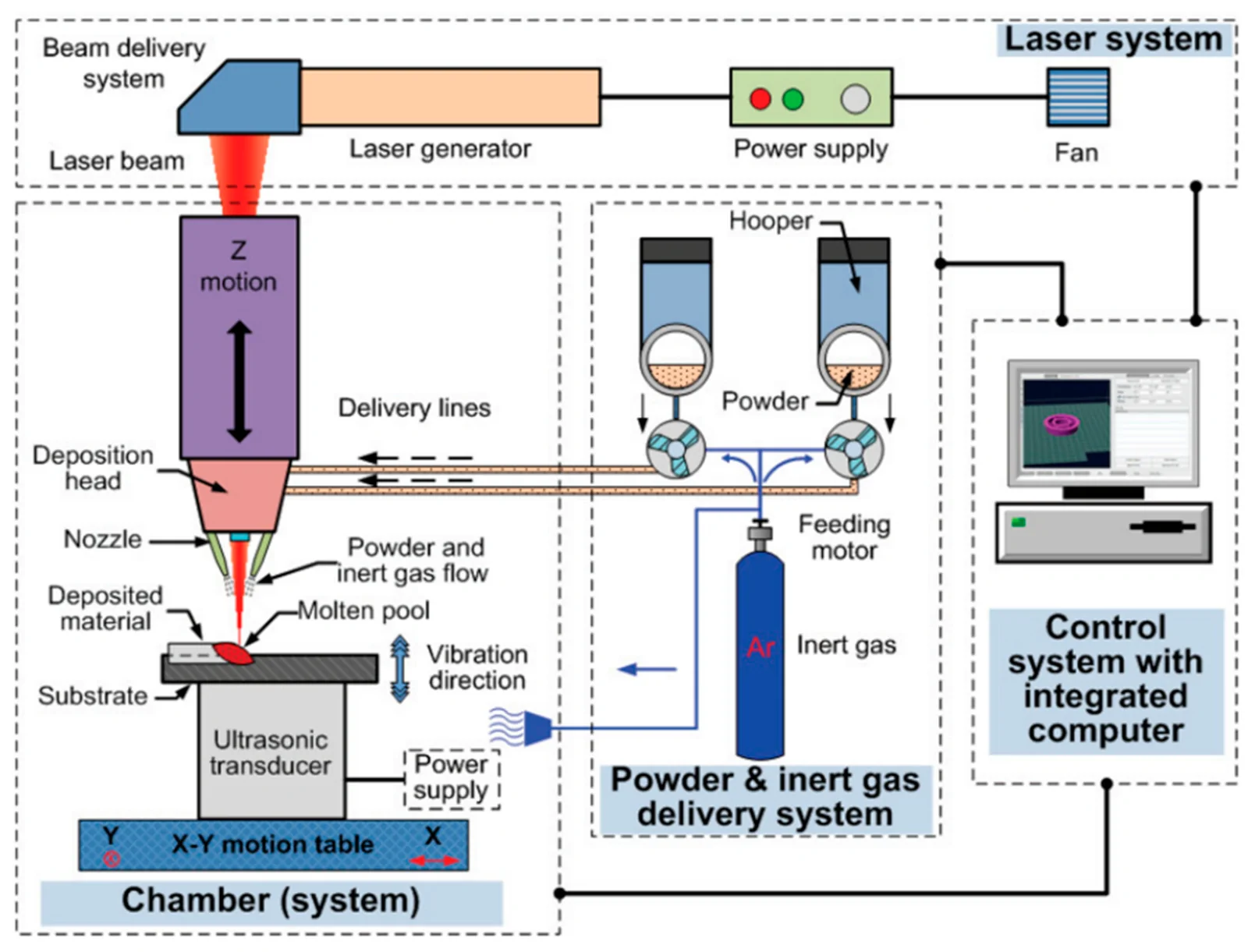
Schematic of ultrasonic vibration-assisted powder DED setup [89]—reproduced under Creative Commons (CC BY NC ND) license.

**Figure 14:**
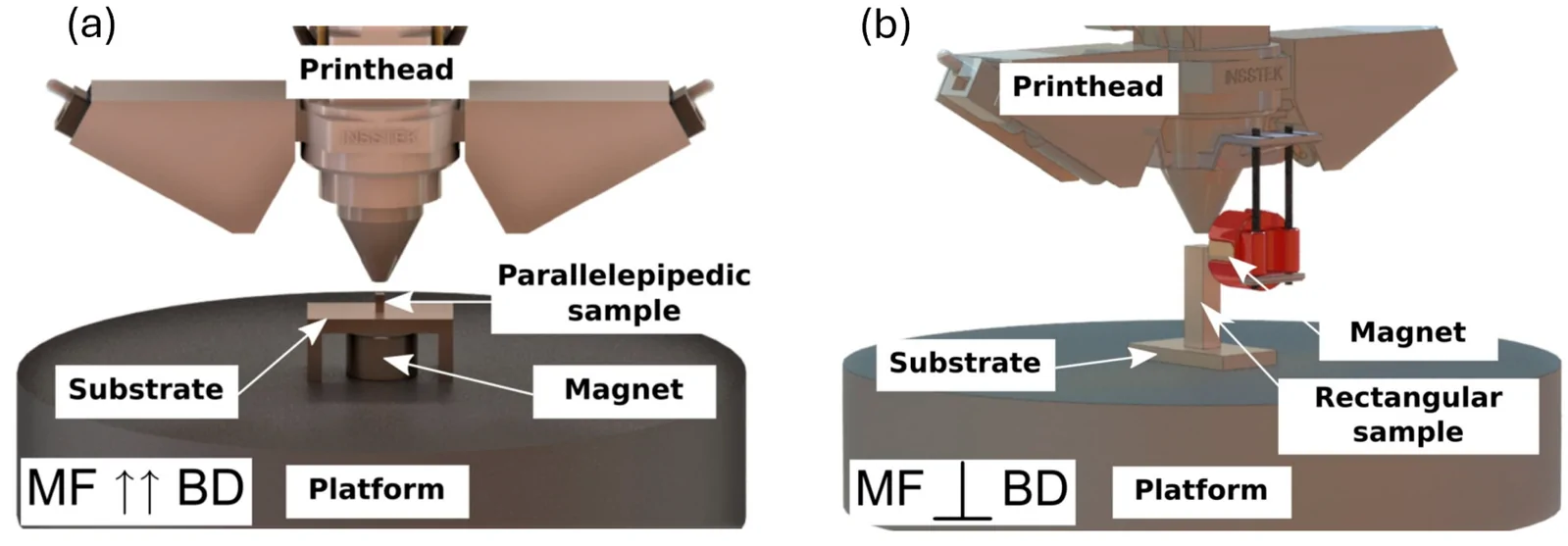
Schematic of powder DED with magnetic field setup (**a**) parallel to and (**b**) normal to the building direction [106]—reproduced with modifications under Creative Commons (CC BY) license.

Various contact and non-contact hybrid strategies were explored in powder DED systems as well. In a study on IN718 [89], an ultrasonic transducer was coupled to the substrate while powder was delivered into a laser-generated melt pool, as illustrated in Figure 13. Unlike forging or alloying approaches, vibration assistance did not directly contact the deposited material. Instead, acoustic streaming and cavitation modified melt-pool solidification for reduced porosity, grain refinement, and fragmentation of the Laves phase. Figure 14 shows another non-contact approach, in which a 0.2 T external magnetic field was developed around the melt pool during powder DED of IN718 [106]. The applied field influenced conductive fluid flow and solute redistribution through magnetohydrodynamic and thermoelectric magnetic effects, reducing Nb-rich phases and modestly improving ductility.

**Figure 15:**
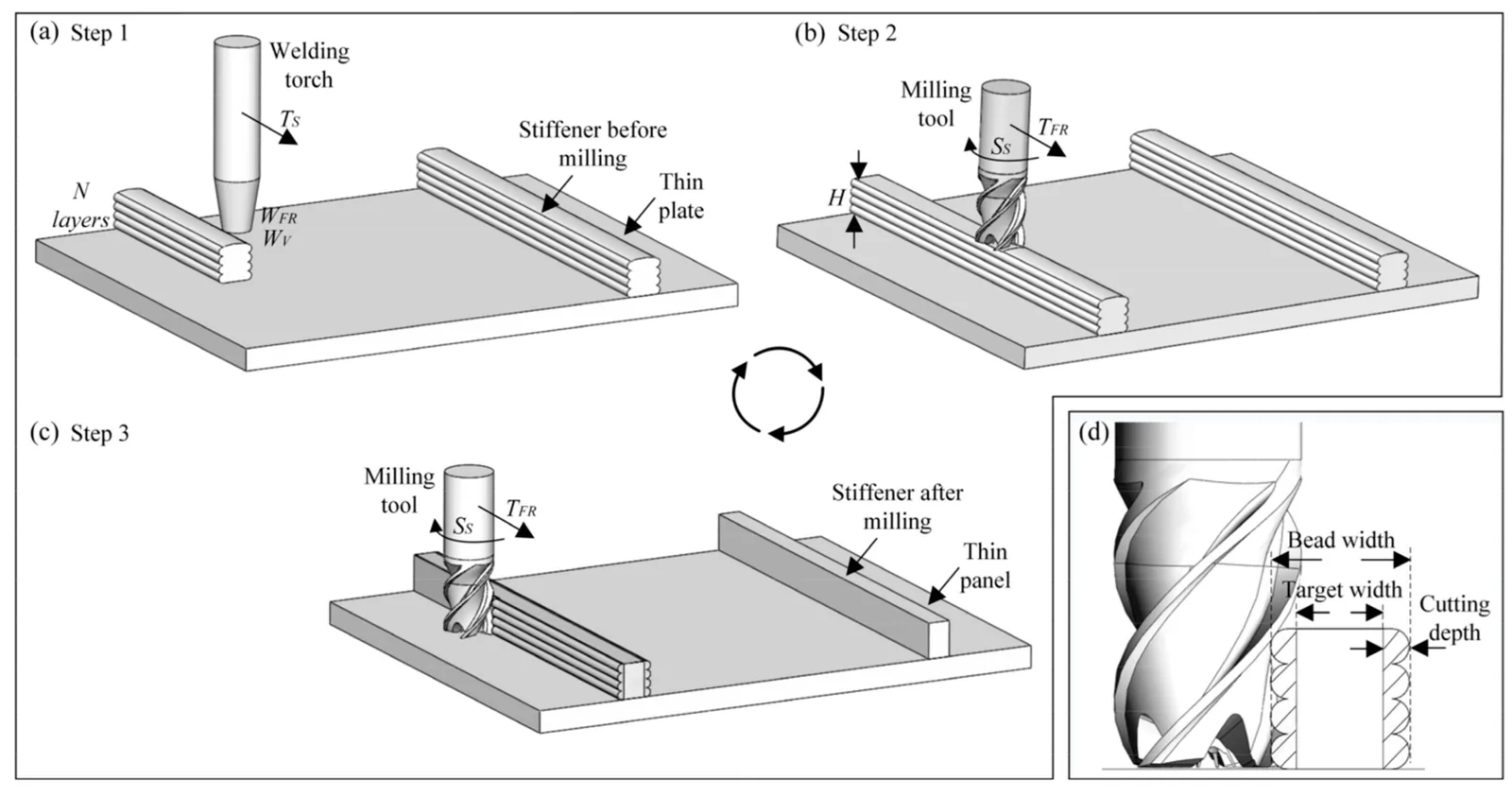
(**a**–**c**) Working principle of wire DED + milling hybrid process for fabricating stiffened panels, (**d**) Illustration of tool position, cutting depth, and bead width [109]—reproduced under Creative Commons (CC BY) license.

In addition to bulk microstructure-focused interventions, some approaches targeted surface condition, interlayer cleanliness, geometry control, or dimensional accuracy. In a wire DED of ER100S-G [108], interlayer surface preparation was evaluated using wire brushing and extensive grinding for slag removal after each deposited layer. Slag removal reduced lack-of-fusion defects, porosity, and oxide inclusions, providing varying levels of improvement in Charpy impact energy at 0 °C from 38.3 ± 7.6 J without treatment to 72.5 ± 7.7 J after wire brushing and 82.5 ± 2.1 J after extensive grinding. Similarly, a wire DED study explored milling as a geometry and surface quality driven strategy for fabricating Al 2325 stiffened panels [109]. In this approach, wire DED was used to deposit stiffeners onto a thin plate, while periodic milling removed irregular top and side surfaces to maintain dimensional accuracy and surface finish (Figure 15) [109]. For a representative stiffened panel, the optimized process achieved an average surface roughness of 1.38 µm, 91% material utilization, and 102 min construction time, compared with 34% material utilization and 166 min for conventional machining from a thick plate.

**Figure 16:**
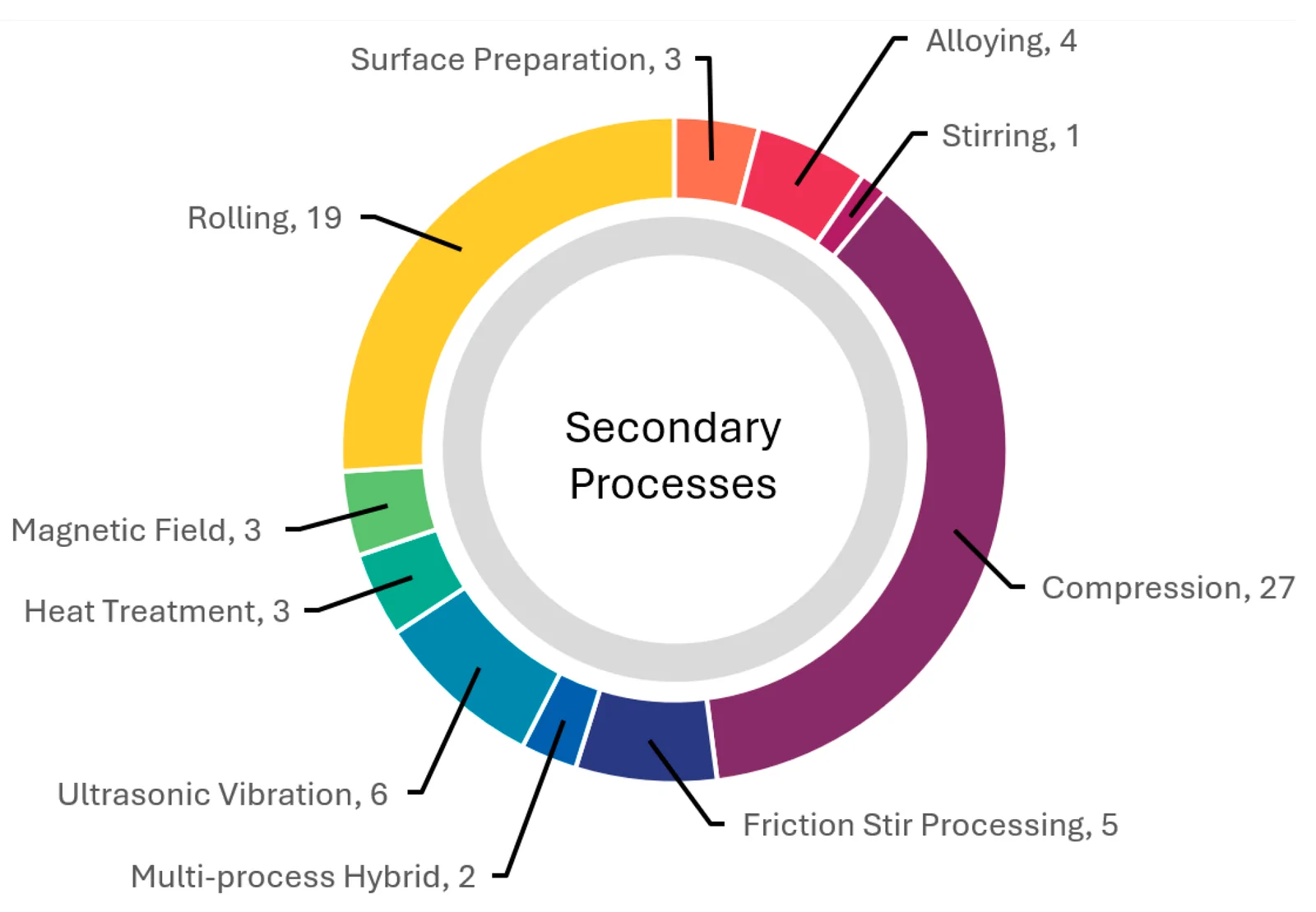
Distribution of secondary processes in the reviewed literature.

The dominance of wire DED in the in-situ hybrid DED literature is likely due to its lower cost, open process architecture, and easier access for tooling setup required for secondary processing. The distribution of secondary-process categories is summarized in Figure 16, including multi-process studies where more than one category of secondary process was intentionally combined. A strong trend is the widespread use of interlayer plastic deformation-based methods, including forging, peening, and rolling. These studies, however, span a broad thermomechanical window. Local forging and peening approaches generally used relatively low forces, from ~17 N to ~2.1 kN, whereas rolling commonly operated in the kN range, reaching up to 90 kN. Reported deformation temperatures also ranged from near-room temperature to warm working at ~250–700 °C and hot deformation at ~800–1150 °C, depending on the alloy system. As a result, the reported mechanisms vary widely, including cold work storage, residual-stress redistribution, pore closure, grain refinement, texture modification, hot recrystallization, and corresponding improvements in mechanical response. It is important to note that in the in-situ hybrid AM literature, terms such as forging, hammering, compression, and peening are used interchangeably for localized loading operations with forces commonly in the hundreds-of-newtons range. In contrast, ex-situ bulk forging generally uses loads orders of magnitude higher, commonly ranging from tens of kilonewtons to meganewtons depending on component size and process scale, whereas peening refers to lower force, near-surface deformation intended primarily for surface modification.

### 3.2. Evolving Trends in Secondary Processing

Since the early reports in 2014 [67], in-situ hybrid DED has shifted from a niche process modification to an increasingly active research direction. More importantly, the role of the secondary process has changed. One such instance can be seen in the emerging subset of multi-process in-situ hybrid DED studies [52,73], classified in Table 3. These works move beyond a single secondary processing approach by combining multiple interventions to act on different stages of material evolution.

**Figure 17:**
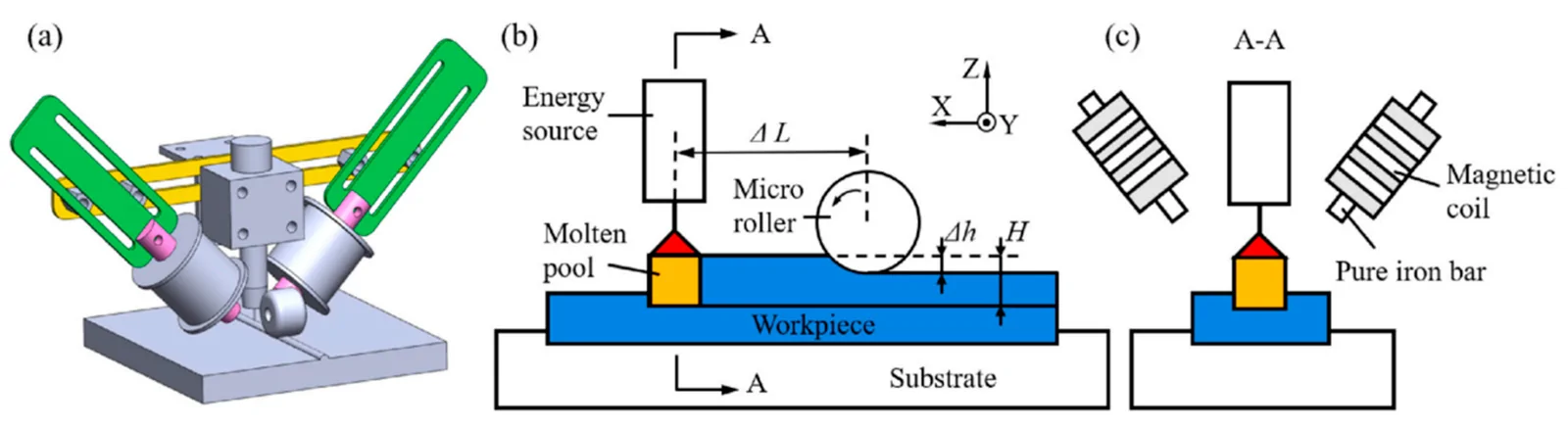
Magnetic field and in-situ hot rolling-assisted wire DED setup shown in (**a**) 3D model, (**b**) schematic normal to material deposition, and (**c**) schematic along the material deposition [73]—reproduced under Creative Commons (CC BY NC ND) license.

**Figure 18:**
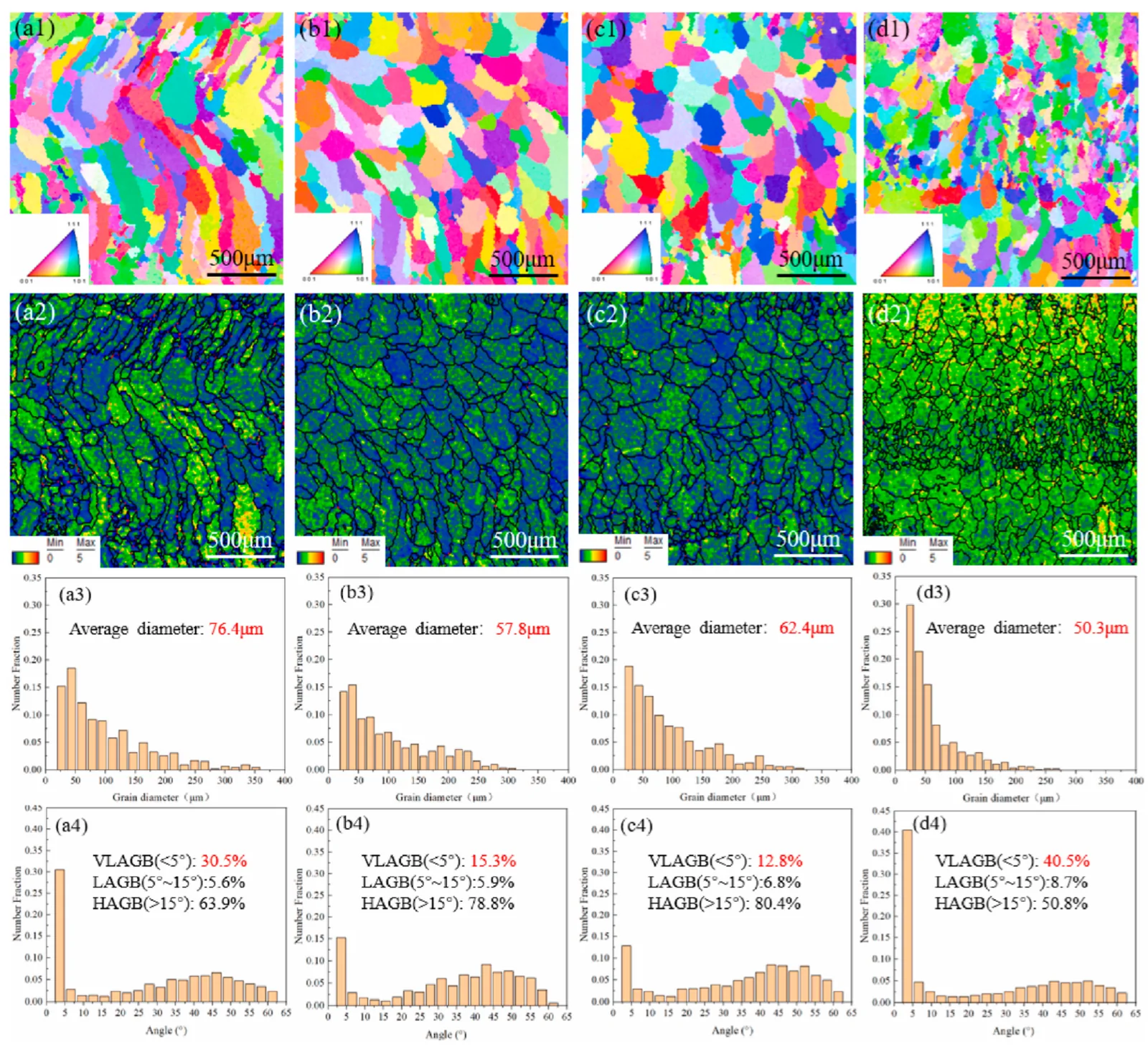
Electron Backscatter Diffraction images and statistics of Wire DED 5087 Al alloy showing (**a1**–**d1**) orientation map (**a2**–**d2**) Kernel Average Misorientation map (**a3**–**d3**) grain size distribution and (**a4**–**d4**) grain boundary angle distribution of (**a**) as-deposited (**b**) 0.2 T magnetic field assisted (**c**) 0.4 T magnetic field assisted (**d**) 0.2 T magnetic field assisted + hot rolled conditions [73]—reproduced under Creative Commons (CC BY NC ND) license.

In wire DED of 5087 Al-Mg alloy [73], magnetic-field assistance and in-situ hot rolling were integrated in the same process, as shown in Figure 17. The system used a 0.2 or 0.4 T magnetic field and a 4 kN roller positioned 30 mm behind the torch, producing ~20% deformation at ~400 °C. Magnetic stirring reduced porosity from 0.76% in as-deposited to 0.40% at 0.2 T, while the combined 0.2 T + hot rolling condition further reduced porosity to 0.23%, grain size from 76.4 to 50.3 µm (Figure 18) [73], and residual stress from 16.9 to 9.4 MPa. This combined condition also improved UTS, YS, and elongation by 16.7%, 19.1%, and 37.3%, respectively, over as-deposited conditions. In this multi-process setup, the magnetic field primarily modified the liquid-state arc and melt-pool behavior, while hot rolling modified the viscoplastic solid through deformation, pore closure, grain refinement, and stress redistribution.

A similar multi-process setup was studied using compound arc and vibration shock forging-rolling (CAVSFR) in wire DED of low-carbon steel [52]. The optimized condition used a vibration-assisted hammering roller, with 10 mm torch-to-roller distance, 136 N static force, and 35 m/s^2^ vibration shock, producing 28.3% deformation and reducing average grain size from 9.6 to 6.4 µm. Grain refinement was attributed to vibration-assisted dendrite fragmentation in the molten pool combined with the effects of medium to high temperature deformation assistance in solidified material. The optimized material reached 555 MPa UTS, 391 MPa YS, and 38.9% elongation in the build direction, compared with 486 MPa, 360 MPa, and 37.1%, respectively, for as-deposited conditions.

### 3.3. Timing of Secondary Processing

**Figure 19:**
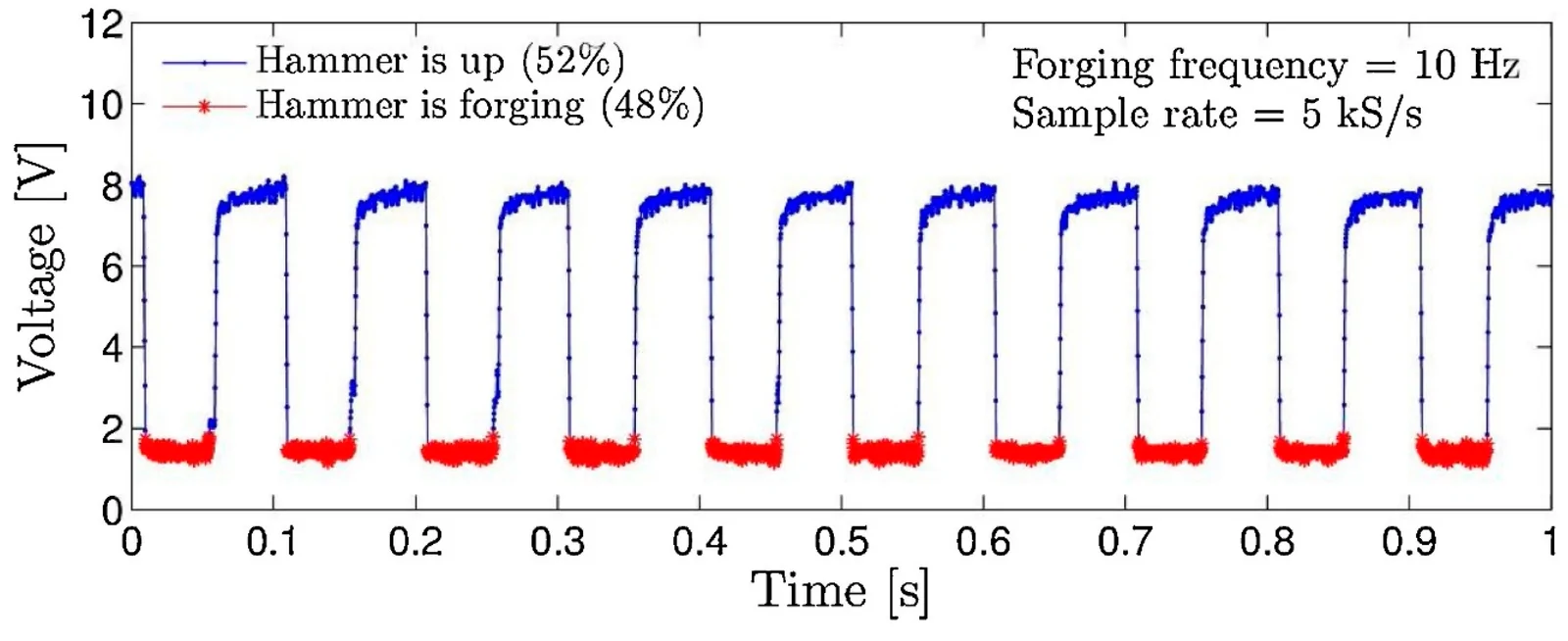
Voltage variation with respect to time was used to detect forging during wire DED of 316L [56]—reproduced under Creative Commons (CC BY NC ND) license.

Across the reviewed studies, the timing of the secondary process intervention was as important as the secondary process itself. In continuous or synchronous approaches, the intervention acted during deposition or immediately after material deposition, while the material was still liquid, semi-solid, or hot/soft enough to respond efficiently. For instance, ultrasonic vibration-assisted powder DED of 17-4 PH stainless steel continuously transmitted vibration through the substrate during deposition [90]. This reduced porosity from 0.68% to 0.35%, changed columnar grains of ~7–11 µm into finer equiaxed grains of ~1.5–3 µm, and increased tensile properties [90]. In powder DED of 316L, hammer forging was applied at ~700 °C and ~20 mm behind the laser spot; a 55 N hammering force produced ~21% deformation, reduced average grain size by 69%, and increased YS, UTS, and hardness to 494 ± 19 MPa, 677 ± 7 MPa, and 243 ± 11 HV0.2, respectively [66]. Similarly, synchronous deformation in wire DED of 316L stainless steel used the high-temperature state of the freshly deposited bead to reduce the required forming load [56]. In this approach, the forging hammer followed the arc at a 14 mm offset and operated at 10 Hz, as shown in Figure 19. At a bead temperature of ~917 °C, forging forces of only 17–55 N were sufficient to promote viscoplastic deformation, pore closure, grain refinement, and texture reduction [56].

A second group of studies used regular interlayer processing, where deposition was paused after each layer and the secondary process was applied before the next layer. In this mode, the interpass wait period based on time or temperature setpoint became an intentional processing step rather than idle time. For instance, interlayer surface preparation in wire DED of ER100S-G low-carbon steel was performed at an interpass temperature of ~50 °C using either wire brushing or extensive grinding after each deposited layer. This step, intended primarily for slag removal, also reduced oxide inclusions, porosity, and lack-of-fusion [108]. In interlayer rolling of wire DED 2319 and 5087 Al alloys, each layer was allowed to cool below 85 °C before rolling at 15, 30, or 45 kN. Porosity was reported to progressively decrease with load, falling by 73.7–83.5% at 15 kN and by ~97% at 30 kN, while 45 kN eliminated optically detectable pores larger than ~5 µm [77].

Periodic interlayer processing offered considerable benefits with lower interruption periods compared with every-layer interventions. In wire DED of 2319 aluminum alloy, friction stir processing (FSP) was applied after every three deposited layers, using 800 rpm rotation and 200 mm/min traverse speed [93]. Within the FSP-affected region, the process refined the average grain size from 19.4 to 5.2 µm and eliminated pores visible in the as-deposited condition, while increasing hardness, YS, and UTS from 65.3 ± 2.8 HV, 96 ± 2 MPa, and 249 ± 3 MPa to 70.6 ± 3.4 HV, 106 ± 3 MPa, and 278 ± 4 MPa, respectively [93]. In powder DED of Ti-6Al-4V, ultrasonic impact peening (UIP) at 20 kHz frequency and 40 µm amplitude was applied twice after every four 0.1 mm layers because of its strengthening depth of ~0.4 mm [63]. Subsequent heat treatment (HT) used three 970 °C/1 h thermal cycles, followed by 950 °C/1 h solution treatment and 550 °C/4 h aging. Compared with DED + HT, DED + UIP + HT increased primary α phase fraction from 38.6% to 76.7%, and reduced anisotropy in YS, UTS, and elongation to 1.3%, 0.5%, and 4.7%, respectively.

Finally, very few studies used secondary process intervention only once at selected stages of the deposition. In wire DED of an Inconel 625—HSLA steel functionally graded material, AM deposition was carried out until the critical 50% Inconel 625—50% HSLA region [103]. The semi-finished component was then heat treated at 1080 °C for either 1 or 2 h, air cooled, and returned to the deposition sequence for completion of the remaining graded regions. This single mid-build intervention was positioned immediately after formation of the region most susceptible to Laves-phase precipitation. Similarly, in a powder DED study on 316L, deformation was selectively applied after the 10th layer in a 20-layer build [67]. Low, moderate, and high deformation were introduced by laser shock peening, shot peening, and hammer peening, respectively, with corresponding affected depths of ~600, ~350, and ~1200 µm. During subsequent deposition of the 11th layer, static recrystallization was reported only in moderate and high deformation conditions, driven by stored strain energy. In the high deformation sample, local hardness increased from 198.5 ± 8.4 HV in the control region to 215.8 ± 3.8 HV in the recrystallized zone.

Together, these studies showed that the secondary process schedule is a critical control variable in in-situ hybrid DED because it determined whether the intervention acted on the liquid melt pool, hot viscoplastic bead, or solidified layer, thereby governing the active mechanism and overall effectiveness of the approach.

### 3.4. Role of Heat Treatment

Heat treatment (HT) appeared in the in-situ hybrid DED literature in two roles: as a post-processing step after an in-situ intervention, typically deformation-based, or as the in-situ secondary process itself. In deformation-assisted routes, post HT acted globally and provided sufficient diffusion time for recovery, recrystallization, phase dissolution, precipitation, and homogenization, often using the stored strain energy introduced during deposition. By contrast, in-situ HT imposed short, localized thermal cycles that enabled selective phase control while limiting grain coarsening and preserving more of the fine AM microstructure and hardness.

**Figure 20:**
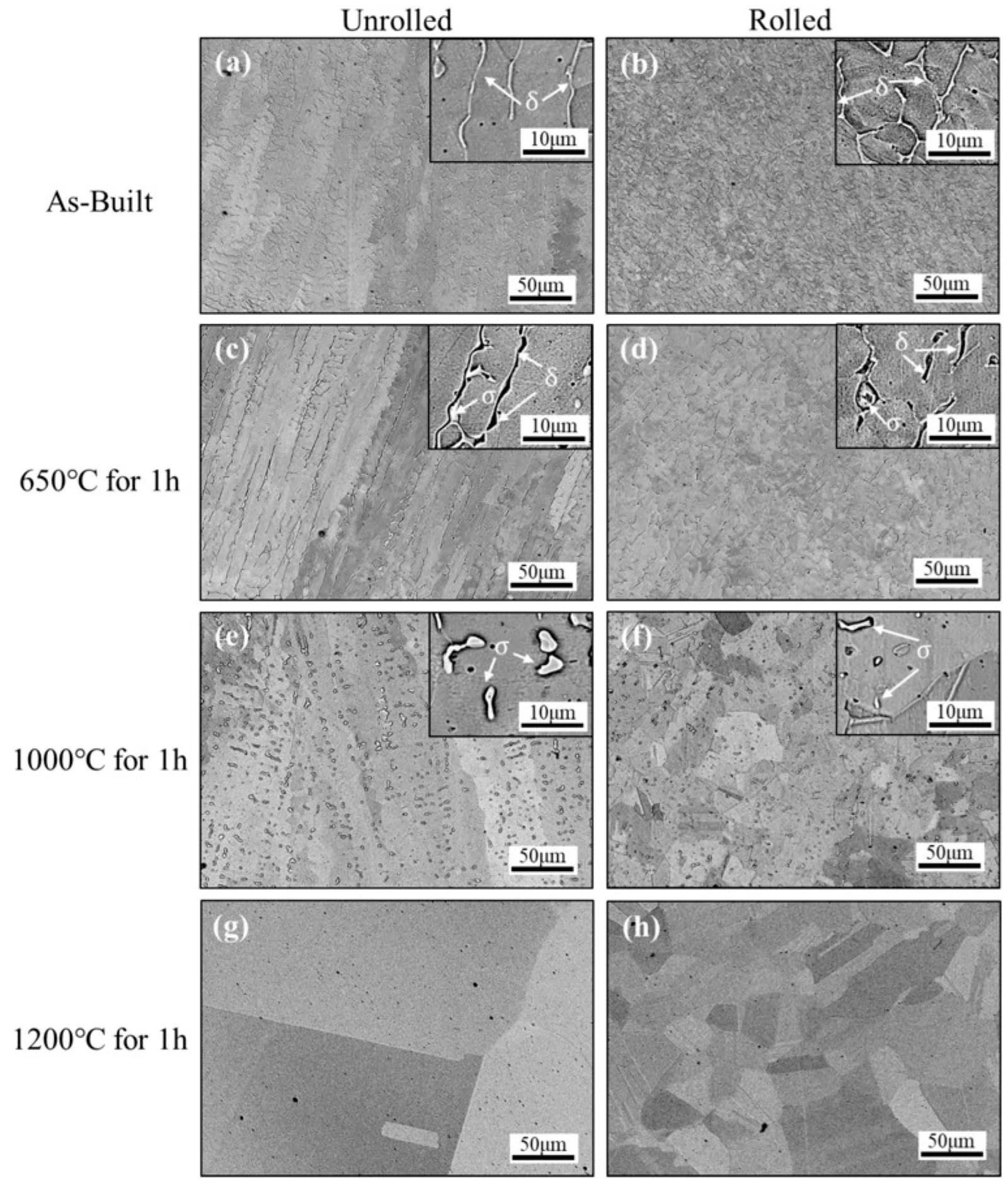
SEM images of as-deposited and as-deposited + rolled wire DED 316L specimens before and after HT indicating the evolution of grains and δ-ferrite: (**a**,**c**,**e**,**g**) as-deposited specimens after no HT, 650 °C/1 h, 1000 °C/1 h, 1200 °C/1 h; (**b**,**d**,**f**,**h**) as-deposited + rolled specimens after no HT, 650 °C/1 h, 1000 °C/1 h, 1200 °C/1 h [83]—reproduced under Creative Commons (CC BY NC ND) license.

The first role of HT is illustrated in Figure 20 by the evolution of 316L microstructure in a rolling-assisted wire DED study with post HT [83]. Rolling at 8 kN while the deposited material was at ~810–830 °C disrupted the continuous dendritic δ-ferrite network and introduced a heavily deformed substructure. The response to subsequent HT is shown in Figure 20 micrographs. After treatment at 650 °C for 1 h, the overall morphology changed little, although σ phase began to form within the retained δ-ferrite. At 1000 °C for 1 h, the non-rolled material retained much of its dendritic morphology, whereas the rolled material recrystallized into fine equiaxed austenite grains of ~22 µm, with residual ferrite and σ appearing as granular or short-rod features. After treatment at 1200 °C for 1 h, ferrite and σ were essentially dissolved in both conditions. However, the effect of prior rolling remained evident, with rolled material reporting finer equiaxed austenite grains of ~45 µm, compared with ~180 µm in the non-rolled material. In this case, rolling provided the deformation structure and stored energy, while post HT determined whether recovery, σ-phase formation, recrystallization, phase dissolution, or grain growth dominated the final microstructure. Heat-treatment-only experiments on thick-wall wire DED 316LSi provide a useful baseline for separating thermal effects from those enabled by prior deformation [111]. Treatments at 732 °C for 2 h and 1000 °C for 1 h progressively dissolved and altered the morphology of δ-ferrite at the microscale while largely preserving the columnar austenite grain structure. Only treatment at 1200 °C for 30 min produced near-complete δ-ferrite dissolution and localized recrystallization into equiaxed grains. Comparatively, these results indicate that prior rolling shifted the response from phase-scale evolution within a retained bulk grain structure to extensive recrystallization at a lower temperature.

Similarly, in Ti-6Al-4V, interpass forging reduced the span of the primary β grains from ~three to four deposited layers to one to two layers and refined the α and α′ constituents [60]. The subsequent treatment at 850 °C for 2 h did not substantially change the primary β-grain size. Instead, it reduced the detectability of the layer and β-grain boundaries, promoted decomposition of the acicular α′ martensite, and modified the α/β morphology. This increased elongation of the forged material to ~13–14% while maintaining an ultimate tensile strength of ~980–1000 MPa. In AerMet100 steel, in-situ rolling at 10 kN fragmented the prior-austenite structure, producing irregular grains of ~40–60 µm [85]. The subsequent multi-step HT formed a lath-martensitic matrix containing fine alloy carbides and film-like reversed austenite along the lath boundaries. The heat-treated material reported ultimate tensile strengths of ~1748–1821 MPa and fracture toughness values up to 70.6 MPa√m, while retaining similar properties in the deposition and build directions.

**Figure 21:**
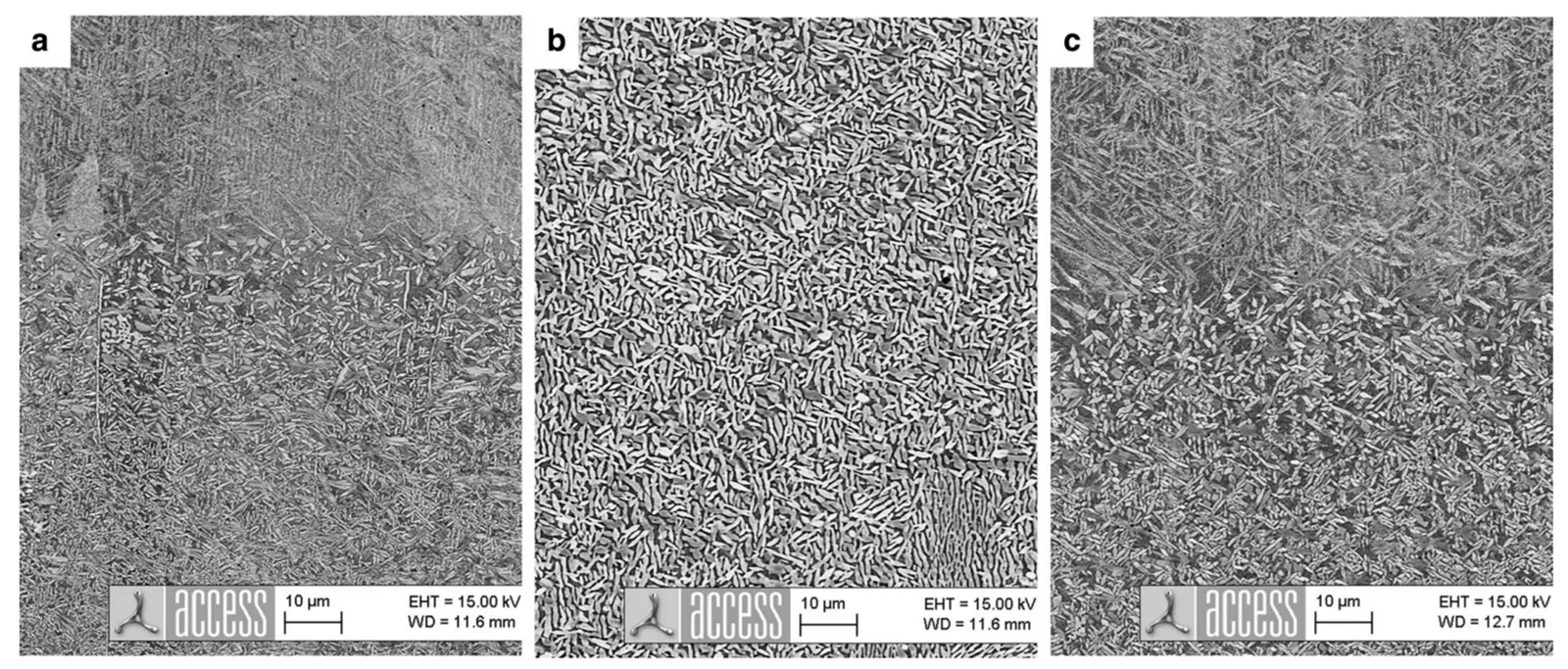
SEM images of powder DED AlCrFe_2_Ni_2_ samples showing the duplex microstructure in (**a**) as-deposited condition, with increase in fcc phase fraction due to (**b**) conventional heat treatment and (**c**) intrinsic heat treatment at 600 mm/min [104]—reproduced under Creative Commons (CC BY) license.

In fewer studies, HT was integrated directly into the deposition sequence. In powder DED of an AlCrFe_2_Ni_2_ medium-entropy alloy (MEA), the same diode laser used for deposition was rescanned over each completed layer at a reduced power and with a similar hatch pattern [104]. As shown in Figure 21a, the as-built material retained the track-wise heterogeneity of the DED process, with thin FCC Widmanstätten plates distributed within the BCC matrix. Conventional furnace HT at 900 °C for 6 h largely removed the visible track boundaries and produced the coarser, more homogeneous FCC-rich duplex structure shown in Figure 21b. By comparison, the in-situ HT in Figure 21c nucleated new FCC plates and thickened existing plates, but the short thermal exposure did not fully remove the track-wise contrast. For this condition, the laser was operated at 105 W, 600 mm/min, and a 1.2 mm track offset, producing transient surface-temperature peaks up to ~1450 °C. The FCC fraction consequently increased from ~40–45% in the as-built material to ~50–55%. Increasing thermal exposure by reducing the scan speed to 480 mm/min and the track offset to 1 mm produced surface peaks of ~1580 °C. This increased the FCC fraction further to ~58–61%. The conventional 900 °C treatment produced a still higher FCC fraction of ~65–70%, but with much greater microstructural coarsening and softening. Consequently, hardness decreased minimally after in-situ treatments, to 388 ± 30 HV0.3 (600 mm/min) and 413 ± 29 HV0.3 (480 mm/min), compared with 441 ± 26 HV0.3 in as-deposited and 275 ± 18 HV0.3 after conventional HT conditions.

A related layer-wise reheating strategy was applied during powder DED of NiTi, where the deposition path was rescanned after each completed layer using the same laser at a reduced power of 30.5 W [105]. The reheating cycle produced a simulated peak temperature of ~1207 °C, exceeding the ~984 °C peritectic temperature of Ti_2_Ni and subsequently passing through the high-temperature solution-treatment range during cooling. This promoted reverse peritectic dissolution and transient solution treatment, reducing the Ti_2_Ni fraction by ~50–70% across the tested deposition conditions. This type of microstructural optimization may be particularly important to the fatigue behavior of NiTi in biomedical applications [112].

Unlike other in-situ HT studies, in Inconel 625—HSLA steel functionally graded material, global heat treatment was performed by interrupting the AM deposition for phase control, by pausing in the critical transition region (50% Inconel 625—50% HSLA) [103]. The entire semi-finished component was heat treated at 1080 °C for 1 or 2 h, air cooled, and then returned to the deposition sequence. This heat treatment dissolved island-like Laves precipitates (~600 nm) and promoted fine Nb-rich carbides associated with dislocation tangles and stacking faults, with greater Laves dissolution after 2 h than after 1 h. Correspondingly, the build-direction tensile strength increased from 512 ± 15 MPa in the continuously deposited condition to 584 ± 10 MPa after 1 h and 602 ± 10 MPa after 2 h treatments.

### 3.5. Mechanistic Pathways and Outcomes

The outcomes of in-situ hybrid processes were not governed by the secondary process alone, but by timing and the mechanism that each intervention activated in the evolving deposit. A useful distinction between recrystallization-centered and non-recrystallization-centered approaches can be recognized. Recrystallization-centered strategies were common because many DED alloys solidify with coarse, textured, and anisotropic grain structures. Across the in-situ hybrid DED literature reviewed, 41 studies reported evidence of recrystallization during the secondary process, during subsequent thermal cycling by next layer deposition, or by post heat treatment. In these studies, recrystallization was rarely the only useful outcome; it was often accompanied by texture modification, pore closure, residual stress redistribution, and improved mechanical response.

However, recrystallization cannot be treated as the only measure of success. Several in-situ hybrid routes were designed to address specific microstructural features or process-based limitations. Interlayer surface preparation improved bonding reliability by removing slag or surface contamination after each layer [108]. In-situ alloying and reinforcement strategies modified solidification through heterogeneous nucleation, growth restriction, segregation control, or particle-assisted strengthening [98,99,100,101]. Ultrasonic vibration and stirring acted mainly through melt-pool agitation, cavitation, acoustic streaming, dendrite fragmentation, and porosity reduction [88,90,91,102]. Magnetic-field-assisted processing altered arc behavior, melt-pool convection, solute redistribution, and texture development through electromagnetic effects [73,106,107]. In-situ heat treatment aimed at phase dissolution, precipitate control, transformation management, or local property tuning rather than deformation-induced recrystallization [103,104,105]. These pathways and outcomes are summarized in Table 4.

### 3.6. Material Systems Explored

**Figure 22:**
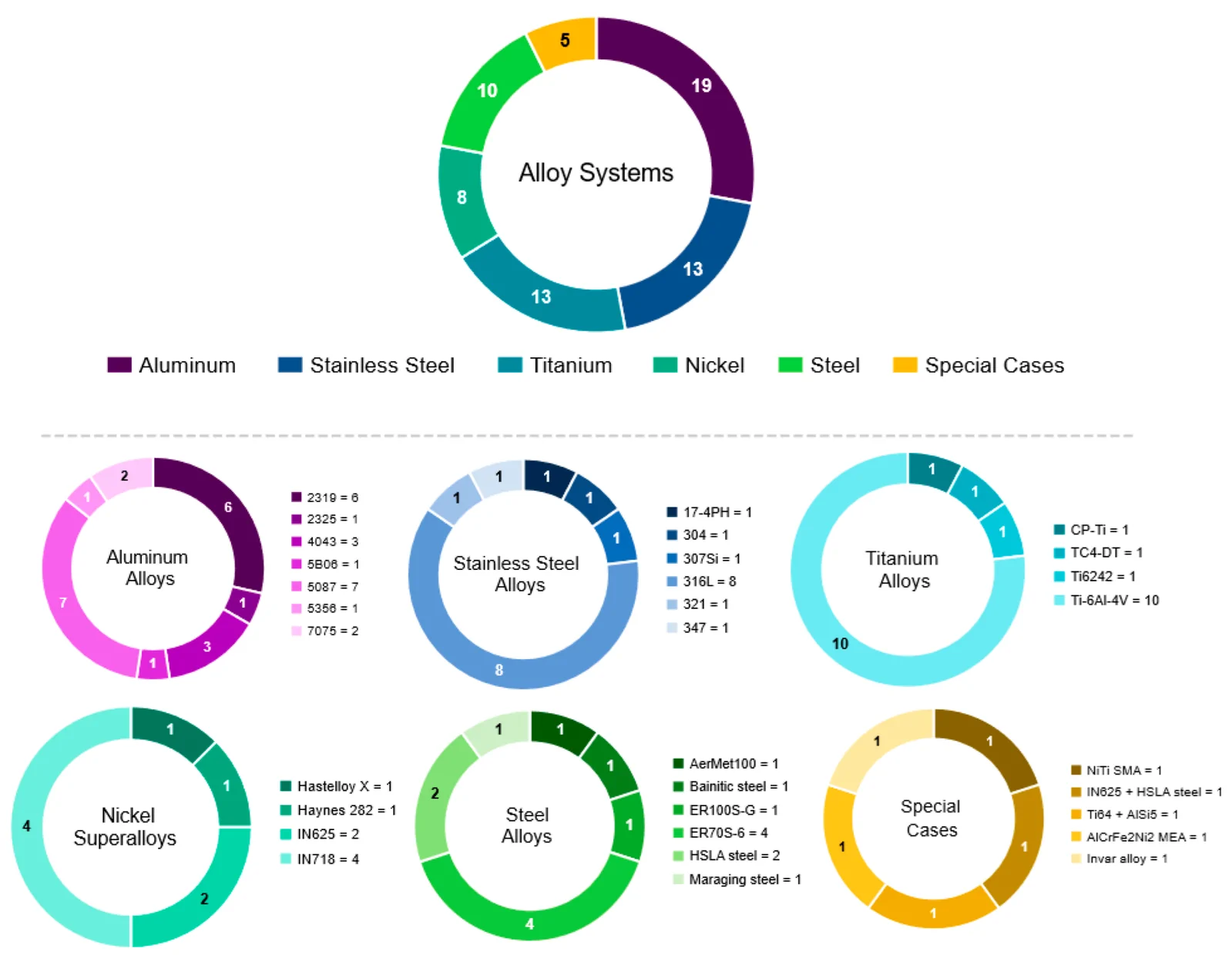
Distribution of alloy systems investigated in in-situ hybrid DED studies.

This section discusses the effectiveness of in-situ hybrid DED approaches across the major alloy families, with particular emphasis on addressing the dominant material-specific limitations. These include porosity in aluminum alloys, columnar grain structure in steel and stainless steels, prior-β transformation control in titanium alloys, interdendritic segregation in Ni-based superalloys, and localized metallurgical challenges in specialty alloys and dissimilar systems. The distribution of material families and individual alloys covered in the reviewed studies is summarized in Figure 22, in which the upper ring reports unique studies by material family, and the lower insets report alloy entries, with studies investigating more than one alloy counted once for each alloy. Thus, the inset totals may exceed the corresponding family total. Each alloy family is discussed below in detail. To support these discussions, the accompanying tables summarize representative quantitative outcomes reported in individual studies. These values are intended to illustrate study-specific changes and broader mechanistic trends rather than serve as normalized cross-study comparisons, since the studies differ in alloy composition, processing conditions, build geometry, thermal history, sampling location, and characterization methodology.

#### 3.6.1. Aluminum Alloys

Aluminum alloys formed the largest material family in the reviewed literature, with all studies based on wire DED. Porosity was the most persistent limitation across this family, particularly near interlayer fusion regions where gas bubbles could be trapped by rapid solidification, dendritic structures, and surface oxide films. Hydrogen originated mainly from moisture and oxides on the wire and substrate surfaces, as well as from the surrounding environment. Aluminum-based wire DED studies reported that hydrogen solubility decreased by nearly twentyfold during solidification. When the hydrogen content exceeded the reduced solubility limit, the remaining liquid became supersaturated, promoting bubble nucleation and hydrogen-pore formation if the bubbles cannot escape before solidification [113]. These pores were reported to reduce the effective load-bearing area and acted as preferential sites for crack initiation.

**Figure 23:**
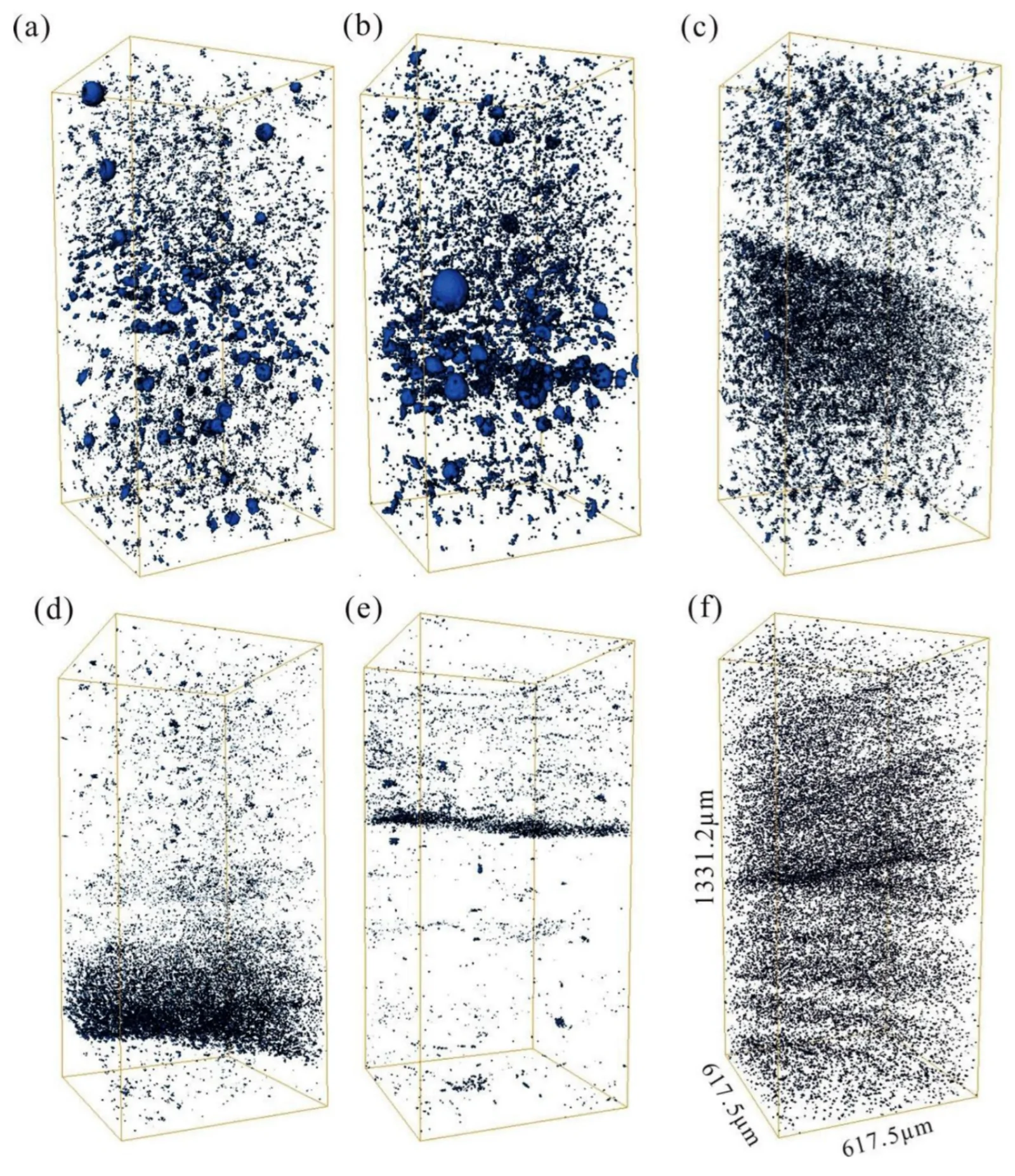
3D view (XCT) of micropores in wire DED Al 5087 alloy: (**a**) as-deposited (**b**) post heat treated without rolling, (**c**–**e**) 15 kN, 30 kN, 45 kN interlayer rolled, and (**f**) 45 kN rolled + heat treated, showing pores reopening [74]—reproduced under Creative Commons (CC BY NC ND) license.

In-situ hybrid DED studies, summarized in Table 5, show that pore closure does not necessarily mean complete pore annihilation. Rolling converted large interlayer pores into flattened cavities, closing smaller pores, and introducing dislocations and vacancies that could trap atomic hydrogen. For instance, 45 kN interlayer rolling reduced pore volume fraction from 1.08% to 0.05% in Al 2319 and from 1.06% to 0.07% in Al 5087 [74]. Subsequent treatment at 535 °C for 90 min increased these fractions to 0.51% and 0.74%, respectively, while producing relatively homogeneous, near-spherical pores with an average diameter of ~5.3 µm (Figure 23). After 45 kN rolling, only 0.8% of the measured hydrogen in 2319 and 1% in 5087 was contained in pores, while the dislocation densities reached 44.1 × 10^13^ and 50.2 × 10^13^ m^−2^. After heat treatment, the dislocation densities decreased to 12.7 × 10^13^ and 11.6 × 10^13^ m^−2^, whereas the fractions of hydrogen contained in pores increased to 7.5% and 8.5%. Thus, the authors conclude that recovery and dislocation annihilation released hydrogen from deformation-induced traps, allowing it to diffuse toward residual cavities and reprecipitate as molecular hydrogen [74].

Typically, some hydrogen remains atomically dissolved or trapped at defects such as dislocations, vacancies, interfaces, and second-phase particles, while molecular hydrogen is retained within pores [114,115]. Rolling could collapse these pores and bring their opposing surfaces into contact without necessarily producing complete metallurgical bonding, resulting in incompletely healed pores [116,117]. During subsequent heat treatment, increased hydrogen mobility, matrix softening, and recovery release hydrogen from deformation-induced traps. Hydrogen accumulation and thermal expansion then increased the internal gas pressure, promoting the reopening, growth, and spheroidization of residual pores [115,117]. Other pore-evolution mechanisms included vacancy migration and coalescence, Ostwald ripening of the existing pore population, and void formation associated with dissolution of soluble second phases. In the reviewed in-situ hybrid DED studies, heat treatment dissolved ~81% of the reticulated second-phase volume in Al 2319 but only 18% in Al 5087. Consequently, pore evolution in Al 5087 was governed mainly by diffusion-controlled Ostwald ripening, whereas Al 2319 additionally developed new voids as the Al-Cu eutectic network dissolved [74,77].

**Figure 24:**
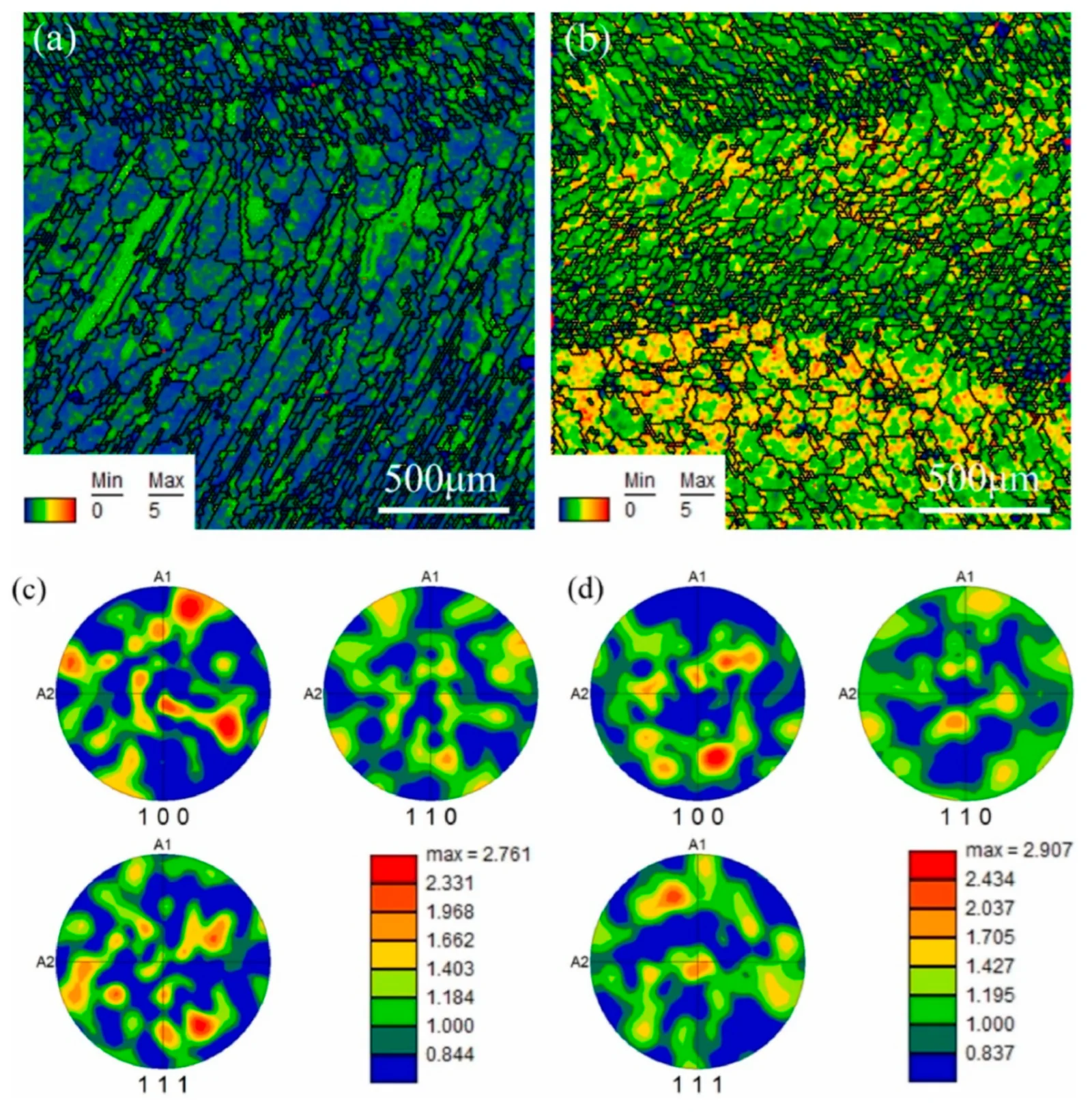
Electron Backscatter Diffraction images of Wire DED 5087 Al alloy showing (**a**,**b**) Kernel Average Misorientation maps and (**c**,**d**) pole figures of (**a**,**c**) as-deposited and (**b**,**d**) deposited + hot rolled conditions showing minimal change in texture and increased local misorientation, consistent with a higher geometrically necessary dislocation density [84]—reproduced under Creative Commons (CC BY NC ND) license.

In non-heat-treatable (i.e., solid solution strengthened) Al-Mg alloys, the benefit of in-situ deformation arose mainly from pore closure and deformation strengthening rather than precipitation. In Al 5087, interlayer hot rolling at ~400 °C applied 20% deformation at 4 kN force, reducing porosity from 1.89% to 0.56% and average pore diameter from 47.3 to 36.1 µm. In the build direction, YS, UTS, and elongation increased from 195 MPa, 263 MPa, and 15.1% to 276 MPa, 338 MPa, and 20.9%, respectively [84]. The geometrically necessary dislocation density increased by an order of magnitude to 7.74 × 10^13^ m^−2^, with the total dislocation density estimated at 1.16–1.50 × 10^14^ m^−2^ (Figure 24). Dislocation strengthening alone contributed ~60–68 MPa, whereas precipitation strengthening was negligible because the secondary phases were coarse and widely spaced. However, recovery and partial recrystallization by subsequent deposition refined the grain structure and retained ductility.

In a non-heat-treatable Al-Si alloy such as Al 4043, the as-deposited strength was partly increased by the continuous Si-rich eutectic network, which restricted dislocation motion but also created brittle crack-propagation paths. Interlayer friction stir processing (FSP) reduced the average α-Al grain size from 40.9 to 1.9 µm, eliminated optically detectable pores from an initial porosity of ~1.2%, and fragmented the Si-rich network into more uniformly dispersed particles [94]. The YS remained nearly unchanged at ~82–88 MPa, while the UTS decreased from 164 MPa to ~148–149 MPa because fragmentation of the Si-rich network reduced the load-bearing contribution of the continuous Si network. In contrast, elongation increased from ~13.8% to 37.5% horizontally and 28.8% vertically. The fatigue limit at 10^7^ cycles reportedly increased from 64 to 82 MPa because pore-controlled crack initiation was removed [94].

For precipitation-hardenable Al-Cu and Al-Zn-Mg-Cu alloys, in-situ deformation similarly removed defects and reconstructed the solidification microstructure, but the final strength remained primarily controlled by precipitate evolution. This was reported in Al 7075, where interlayer FSP eliminated visible pores, fragmented or dissolved the continuous Mg(Al,Zn,Cu)_2_ grain-boundary eutectic, and transformed coarse columnar grains into equiaxed grains smaller than 1.77 µm [96]. The top-layer stir zone reached a YS of 388 MPa, UTS of 504 MPa, and elongation of 16.1%, whereas the thermally stabilized lower region averaged 205 MPa YS, 348 MPa UTS, and 19% elongation. Grain size changed only slightly from 1.69 to 1.77 µm, contributing an estimated ~4 MPa to the build-height YS variation. The much larger property decrease was attributed to repeated thermal cycling, which coarsened η′ and η precipitates and depleted strengthening solute atoms. Overall, porosity control was the most common objective across the aluminum studies; however, the benefits of controlling or removing it depended on the specific alloy class.

#### 3.6.2. Stainless Steel Alloys

In stainless steel alloys, in-situ hybrid processing was used to disrupt directional solidification and control grain growth. This was particularly important for the most explored 316L alloy, where repeated remelting and epitaxial growth produced coarse columnar austenite grains, strong texture, and anisotropic mechanical response. The data summarized in Table 6 show that substantial refinement can occur when deformation and subsequent thermal exposure act together. Deformation introduced dislocations, subgrains, low-angle grain boundaries, and stored strain energy, while reheating by later deposition promoted recovery and recrystallization. The response, therefore, depended on the applied force (strain), strain rate, deformation temperature, deposition heat input, and the reheating or remelting of the affected region by subsequent layer deposition.

**Figure 25:**
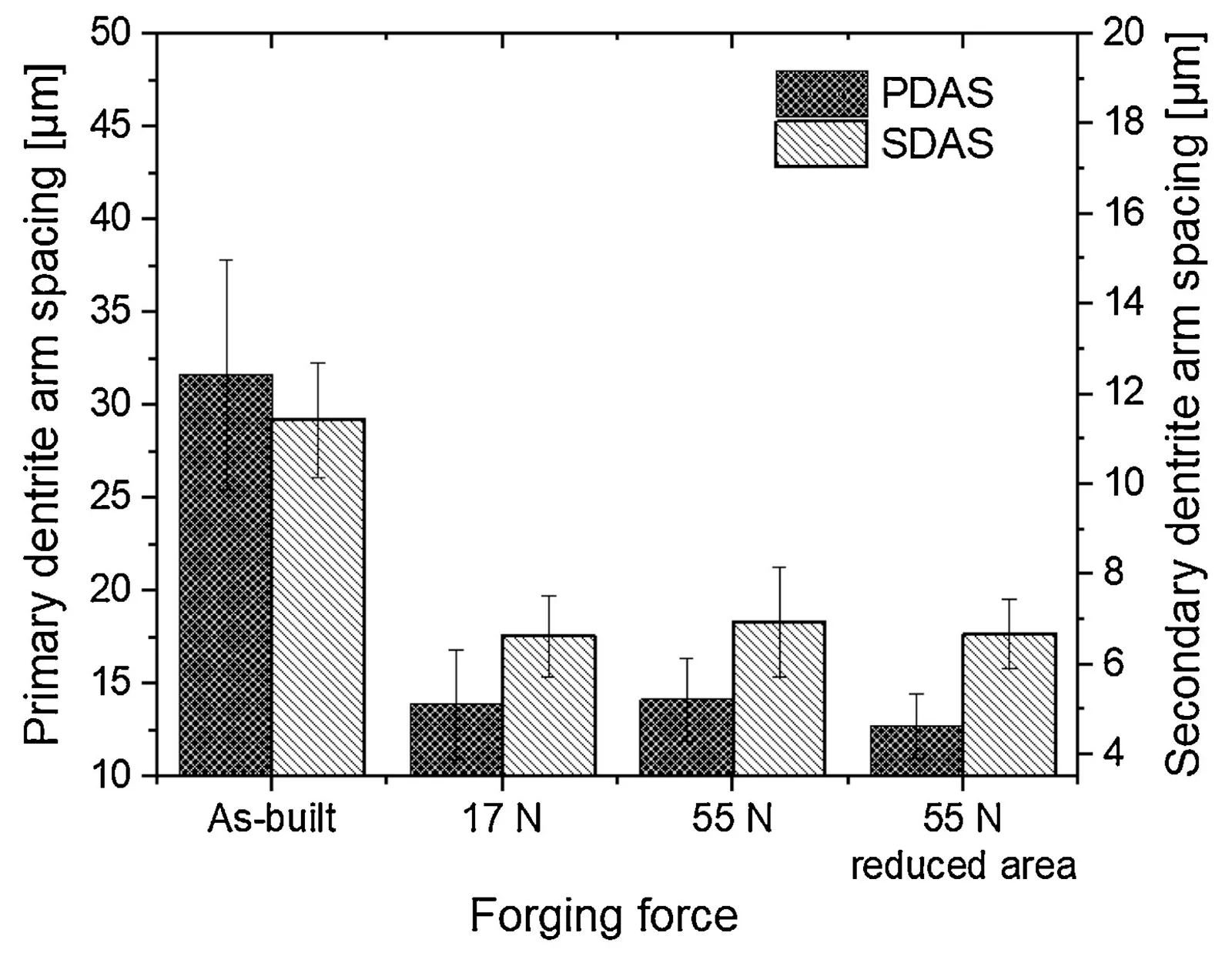
Primary and secondary dendrite arm spacings measured in Wire DED 316L specimens in all conditions [56]—reproduced under Creative Commons (CC BY NC ND) license.

In hot forged wire DED of 316L, deformation was applied immediately behind the deposition torch while the material remained near 900 °C [56]. Low forging forces of 17–55 N were sufficient at this temperature to plastically deform the deposited material and refine the freshly deposited layer, thus increasing the number of nucleation sites during partial remelting and resolidification associated with subsequent deposition. At 55 N, reducing the hammer contact area from 78.5 to 27 mm^2^ further increased the local stress and resulting deformation. Across the forged conditions, the primary dendrite arm spacing decreased from ~32 to 13–15 µm and the secondary spacing from ~12 to 7–8 µm (Figure 25). Synchrotron diffraction also showed weaker solidification texture after forging. Consequently, YS increased from about 360 to 450 MPa and UTS from 574 to 622 MPa, although elongation decreased from 32.5% to 27.9%, indicating a strength-ductility trade-off associated with grain refinement and deformation hardening.

**Figure 26:**
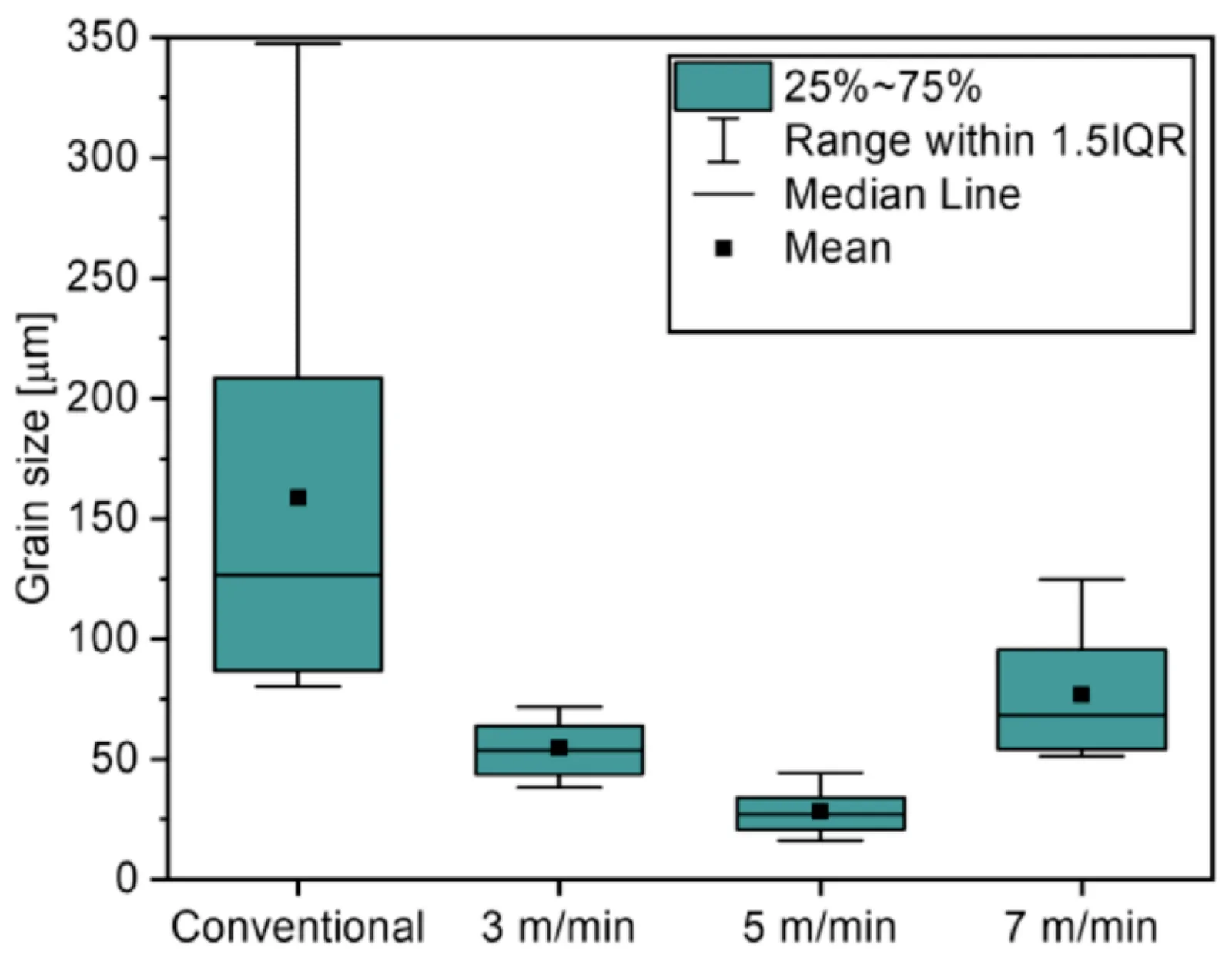
Grain size measured for wire DED of 316L through the intercept method in non-forged (conventional at 3 m/min) and forged conditions (with varying wire-feed speeds) [58]—reproduced with modifications under Creative Commons (CC BY) license.

A more recent study examined the relationship between deposition heat input and hot forging in wire DED of 316L [58]. A pneumatic hammer applied ~2.1 kN at 109 Hz, with deformation occurring within the 950–1150 °C hot-working range for 316L. Wire-feed speeds of 3, 5, and 7 m/min were varied to achieve heat inputs of 0.21, 0.35, and 0.42 kJ/mm, respectively. The conventional non-hammered condition contained coarse columnar grains with a mean size of 158.6 µm and a strong <100>/{200} solidification texture. As shown in Figure 26, hot forging transformed this structure into finer equiaxed grains, reducing the mean grain size to ~55 µm at 3 m/min, 28 µm at 5 m/min, and 76 µm at 7 m/min, while lowering the texture index to below 3. The non-monotonic grain-size response shows that refinement depended on the balance between deformation, recrystallization, and subsequent remelting. The intermediate heat input produced the finest grains, whereas the deeper penetration and greater thermal exposure at 7 m/min WFS remelted more of the deformed region and promoted grain coarsening. Grain refinement was attributed mainly to dynamic recrystallization, followed by static recrystallization and grain growth during subsequent reheating. Consequently, microhardness increased from 175 ± 9 to 238 ± 11 HV at 0.21 kJ/mm, from 172 ± 5 to 264 ± 19 HV at 0.35 kJ/mm, and from 164 ± 8 to 244 ± 11 HV at 0.42 kJ/mm. Meanwhile, the δ-ferrite content decreased only modestly, from 6.6–7.2% without forging to 4.5–5.1% after forging, indicating that the hardness increase arose primarily from grain boundary and dislocation strengthening [58].

A related powder DED 316L study showed that grain refinement changed with depth below the peened surface [67]. Laser-shock, shot, and hammer peening were used to impose low, moderate, and high deformation, producing approximate affected depths of 600, 350, and 1200 µm, respectively. During subsequent deposition, the upper ~150 µm was remelted, erasing the deformation history in that region. Below the remelt boundary, the minimal deformation induced did not provide sufficient stored strain energy for recrystallization, whereas moderate and high deformation produced recrystallized bands ~100 and ~450 µm thick, respectively, with the high-deformation band containing equiaxed grains of 12.8 ± 2.4 µm. EBSD showed many twin boundaries and low internal misorientation within the recrystallized bands. Deeper regions retained higher hardness because the temperature was too low for recrystallization to remove the deformation structure. Peening did not noticeably change the melt-pool size, but the material deposited above the interface still developed finer grains and subgrains. This was correlated with static recrystallization below the interface and improved nucleation during resolidification above it. Therefore, the final microstructure depended on how deformation depth, remelting depth, and reheating overlapped, rather than on deformation level alone.

Beyond deformation-based hybrid approaches, including in-situ rolling [82,83], a non-contact refinement route was demonstrated using ultrasonic-vibration-assisted powder DED of 17-4 PH stainless steel [90]. Unlike other in-situ hybrid DED 316L studies, the influence of ultrasonic vibration acted directly on the liquid melt pool. Acoustic streaming and cavitation were proposed to disturb the solid-liquid interface, detach dendrite tips, and transport them into the undercooled liquid, where they promoted new grain nucleation. This changed the columnar grain structure to equiaxed grains of ~1.5–3 µm. Acoustic pressure and the resulting melt stirring also reduced porosity from 0.68% to 0.35%. As a result, UTS, YS, ductility, and toughness increased by 144.2%, 117.7%, 108.5%, and 313.9%, respectively.

#### 3.6.3. Titanium Alloys

Across the reviewed titanium studies, in-situ hybrid processing primarily aimed to disrupt the columnar prior-β structure, promote finer or more equiaxed regions, modify α/α′ morphology, and weaken the as-deposited texture. The resulting response was governed by the effectiveness of interrupting prior-β inheritance across successive layers and altering the subsequent β→α transformation, as summarized in Table 7.

**Figure 27:**
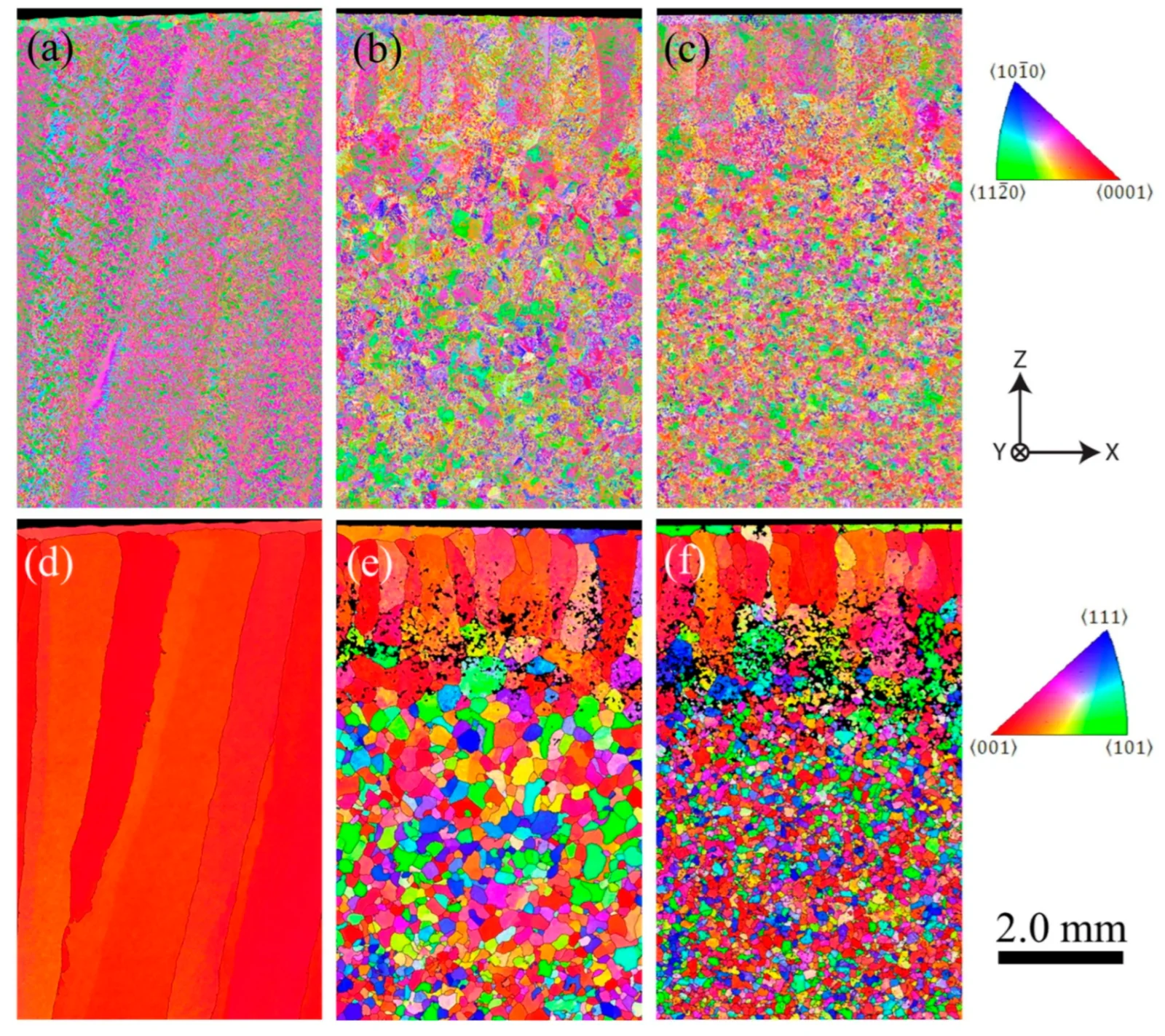
(**a**–**c**) Measured α-phase and (**d**–**f**) reconstructed parent β-phase inverse pole figure orientation maps from the mid-plane x-z sections of (**a**,**d**) as-deposited and in-situ rolled Ti-6Al-4V wire DED using (**b**,**e**) 50 kN and (**c**,**f**) 75 kN loads [79]—reproduced under Creative Commons (CC BY) license.

Among the deformation-based approaches, interpass rolling demonstrated substantial capability to disrupt the repeated regrowth of columnar prior-β grains in wire DED of Ti-6Al-4V [79]. The non-rolled material contained prior-β grains ~2 mm wide and several centimeters long, together with a strong ⟨001⟩ β fiber texture. Rolling each layer below 300 °C at 50 and 75 kN introduced average compressive strains of ~8% and 19%, respectively. As shown in Figure 27, the measured α-phase maps retained a clear memory of the parent β structure, while reconstruction of the β phase revealed the transition from coarse columnar grains in the undeformed wall to much finer, more equiaxed grains after rolling. Following reheating by subsequent deposition, the mean prior-β grain size in the wall center decreased to 130 and 94 µm at 50 and 75 kN, respectively. The maximum β-texture intensity decreased to 3.5 and 2.8 multiples of a random distribution (MRD), while the corresponding α-texture intensities decreased to 2.7 and 2.2 MRD. Because these strains were too low for conventional β recrystallization, refinement was attributed to deformation heterogeneities that generated new β orientations during the subsequent α→β transformation, thereby interrupting epitaxial inheritance of the original β grains. However, deformation was concentrated ~1.5–2.5 mm below the surface and near the wall center, producing a refined core with coarser outer regions. The final layer also retained a columnar structure because it was not reheated after rolling, confirming that deformation and subsequent exposure above the β transus had to act together.

A related thermomechanical response was observed in powder DED of Ti-6Al-4V subjected to ultrasonic impact peening every four layers, followed by thermal cycling, solution treatment, and aging [63]. The as-deposited structure contained columnar prior-β grains 100–400 µm wide, α/α′ laths ~570 nm thick, and β laths ~58 nm thick. Peening at 20 kHz produced an affected depth of ~0.4 mm and introduced fractured α/α′ laths, dislocation networks, cells, tangles, and mechanical twins. It also changed the surface residual stresses from 43 and 159 MPa in tension to −527 and −605 MPa in compression along two orthogonal in-plane directions. These deformation features subsequently promoted thermal grooving and boundary splitting during heat treatment. Compared with heat treatment alone, peening followed by heat treatment increased the primary α fraction from 38.6% to 76.7%, increased α size from 4.0 to 9.9 µm, and reduced its aspect ratio from 2.8 to ~1.5, producing a bimodal structure of globular α and fine α + β lamellae. As a result, YS, UTS, and elongation reached ~910–922 MPa, 1022–1027 MPa, and 14.2–14.9%, respectively, while their directional anisotropies decreased significantly. Thus, unlike rolling, which primarily refined prior-β grains and weakened texture, peening combined with heat treatment additionally reconstructed the α morphology and restored ductility.

**Figure 28:**
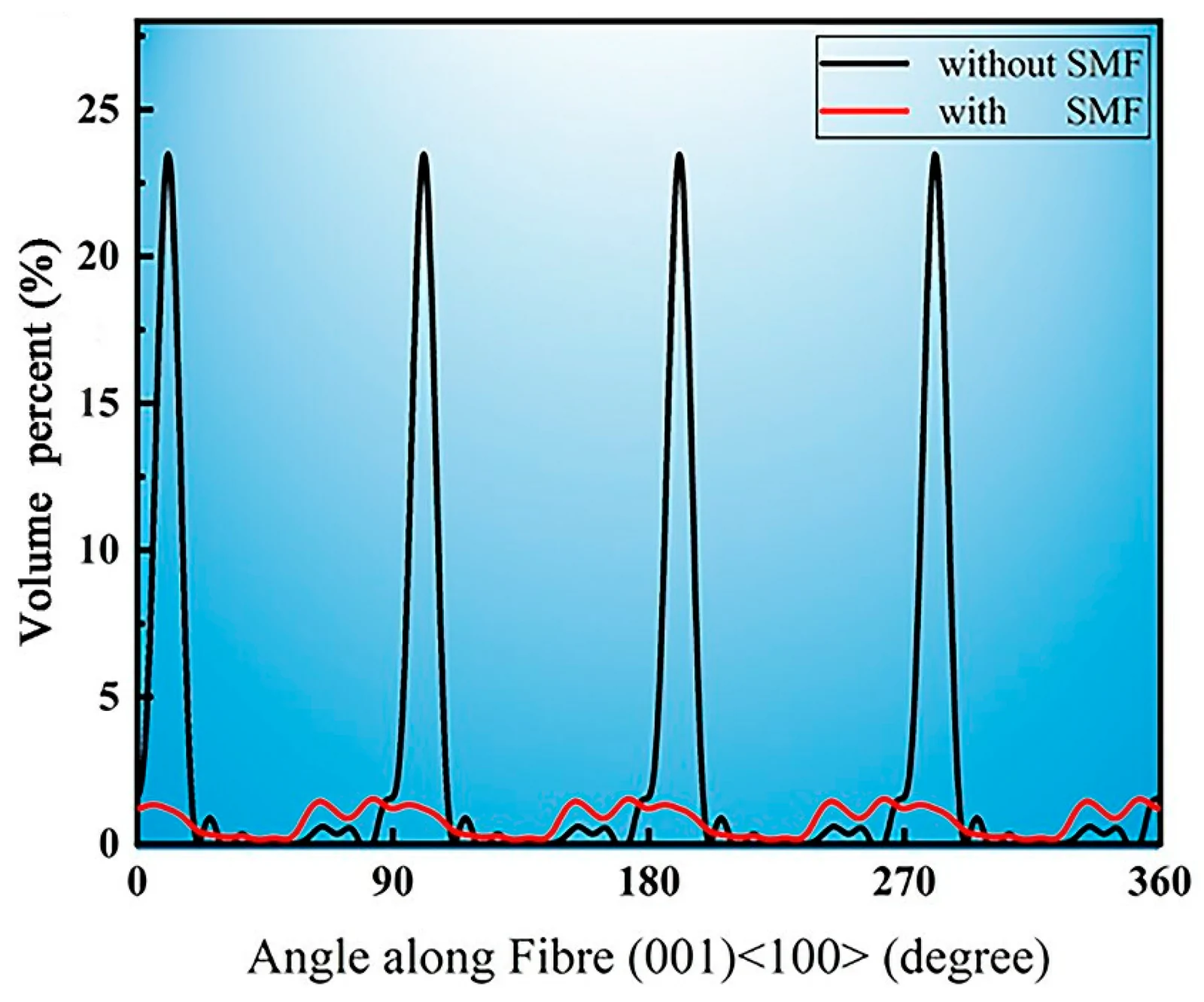
Volume percent of preferred orientations of samples without and with static magnetic field (SMF) along (001) <100> fiber texture in powder DED of Ti-6Al-4V [107]—reproduced under Creative Commons (CC BY) license.

Beyond deformation-based approaches, static magnetic-field (SMF) assistance provided a non-contact route for modifying solidification in powder DED of Ti-6Al-4V [107]. A transverse field of 0.55 T generated thermoelectric magnetic forces, promoting melt convection, dendrite-tip fragmentation, and rotation of the growing β grains. This reduced prior-β grain width from ~0.91 to 0.57 mm and changed the strong ⟨001⟩β texture to a weaker ⟨110⟩ texture. As shown in Figure 28, the volume fraction of the preferred (001) ⟨100⟩ fiber component decreased under SMF. The maximum β-texture intensity decreased from 11 to 8.2 multiples of uniform distribution (MUD), while the α-texture intensity decreased from 38 to 25 MUD. The altered β orientations also changed the subsequent β→α transformation, producing more α variants and converting continuous grain-boundary α into discontinuous segments. Consequently, longitudinal elongation increased from 3.4 ± 0.7% to 10.8 ± 2.8%, while transverse elongation increased from 11.2 ± 1.1% to 12.6 ± 1.7%. This improvement was accompanied by a decrease in longitudinal YS and UTS from 913.1 and 1030.0 MPa to 788.5 and 920.6 MPa, respectively. Thus, the improved ductility arose from the combined effects of narrower β grains, weaker texture, and discontinuous grain-boundary α, which reduced preferential fracture along the prior-β boundaries [107].

Beyond Ti-6Al-4V, an in-situ alloying study demonstrated a chemistry-based route for controlling solidification in wire DED of commercially pure titanium (CP-Ti Grade 1) [99]. Silicon was introduced before each layer to promote constitutional supercooling and restrict β-grain growth. Increasing the Si content from ~0.04 to 0.19 and 0.75 wt.% increased the prior-β grain density from ~0.7 to 1.6 and 3.1 grains/mm^2^, respectively, while reducing grain size from ~1.1–1.2 mm to 0.55–0.60 mm, at 0.75 wt.%. Silicon mainly reduced the width of the columnar grains and enabled limited equiaxed nucleation but did not eliminate their vertical continuity. Repeated remelting removed many initially formed fine grains and restored epitaxial growth, while comparison with cast Ti-Si indicated that DED contained a lower density of activated nucleants. Thus, growth restriction alone reduced columnar-grain width, whereas a complete columnar-to-equiaxed transition required both solute-induced undercooling and a sufficient population of potent nucleation sites [99].

#### 3.6.4. Nickel-Based Superalloys

Nickel-based superalloy in-situ hybrid AM studies focused on interdendritic segregation of refractory alloying elements. This was particularly important in Nb-bearing IN718 and IN625, where Nb and Mo accumulated in the residual interdendritic liquid, promoting Laves phase formation. Coarse or continuous Laves constituents can provide preferential crack paths and deplete the γ matrix of Nb and Mo, thereby limiting γ″ precipitation in IN718 [118] and reducing mechanical properties. In-situ strategies reduced these effects by fragmenting and dissolving the Laves phase, suppressing its formation through inoculation, or redistributing interdendritic solute.

A powder DED investigation on IN718 employing interlayer hammering demonstrated effective control of both Laves phase and grain structure [64]. The as-deposited material exhibited coarse grains with an average size of 206.6 µm and Laves-phase features measuring up to ~1.7 µm. After interlayer hammering, the maximum feature size decreased to 1.1 µm, and dislocations, subgrains, and twins were introduced. Compared with the as-deposited condition, repeated hammering and reheating produced alternating fine and coarse-grained regions with average grain sizes of 12.0 and 68.9 µm, respectively, while the corresponding Laves-phase fractions decreased to 0.26% and 0.47%, respectively. This reduction was attributed to reheating during subsequent deposition, which promoted recrystallization and dissolution of fragmented constituents through shorter diffusion distances and enhanced niobium transport along deformation-induced defects. Correspondingly, horizontal ultimate tensile strength (UTS) and yield strength (YS) increased from 810 and 624 MPa to 1075 and 901 MPa, respectively, and elongation improved from 13.9% to 17%.

**Figure 29:**
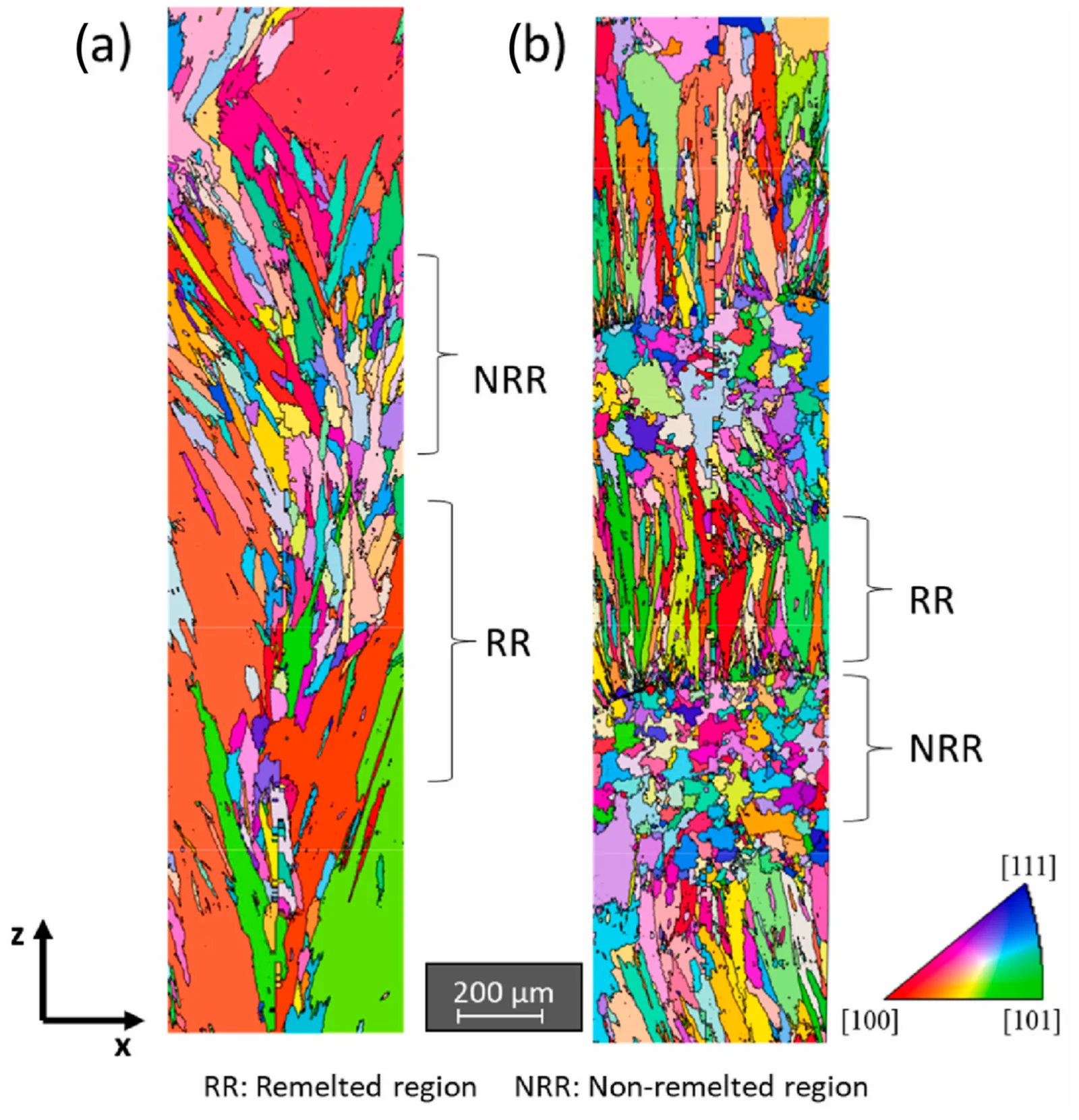
Electron Backscatter Diffraction orientation maps showing grain size variation between (**a**) as-deposited and (**b**) in-situ TiB_2_ alloyed conditions in wire DED IN625 study [98]—reproduced with modifications under Creative Commons (CC BY) license.

A wire DED investigation on IN625 employed in-situ TiB_2_ inoculation to control grain structure and interdendritic segregation [98]. By adding 0.56 wt.% TiB_2_, non-remelted regions transitioned from columnar to equiaxed grains, although some columnar grains persisted in remelted regions (Figure 29). As a result, the average grain area decreased from 1823 to 583 µm^2^, the dendrite width declined from 39.1 to 14.8 µm, and the maximum texture intensity decreased from 5.4 to 2.2. The study also observed smaller and less continuous Nb-rich Laves-phase regions. These modifications were attributed to increased heterogeneous nucleation and reduced time for Nb and Mo enrichment in the residual liquid. Partial dissolution of TiB_2_ was predicted to decrease the solidification range from 363 to 257 °C. Horizontal and vertical compressive yield strength increased from 386 and 376 MPa to 407 and 405 MPa, respectively.

A separate powder DED study on IN718 investigated the influence of a static magnetic field on the formation of Nb-rich secondary phases [106]. Application of a ~0.2 T magnetic field resulted in minimal grain refinement. In contrast, the fraction of Nb-rich phase decreased from 14.1 to 9.1 wt.%, planar porosity declined from 0.3% to 0.2%, and average elongation increased from 23% to 27%. The authors attributed these changes to thermoelectric magnetic convection within the mushy zone, which altered the redistribution of Nb and Mo despite exerting only a weak influence on bulk melt-pool flow.

**Figure 30:**
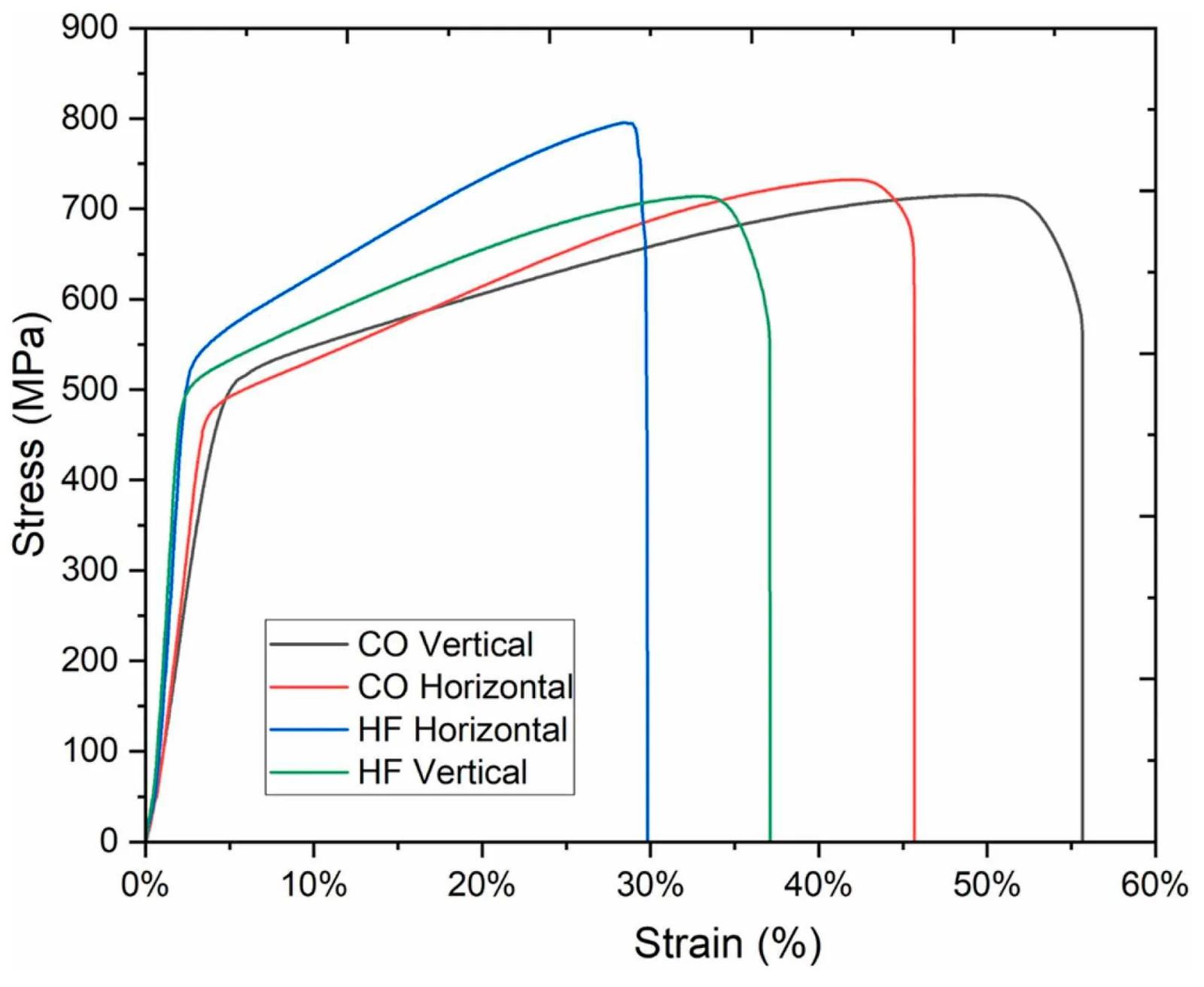
Uniaxial tensile test data of wire DED Haynes^®^282 in the vertical and horizontal directions for hot forged (HF) and as-deposited (CO) conditions [43]—reproduced under Creative Commons (CC BY) license.

Beyond Inconel alloys, a wire DED study used in-situ hot forging to address coarse columnar grains, texture, and internal voids in Haynes 282 [43]. A crowned hammer operated at 4 bar and 8 Hz, delivering an estimated impact force of 1022 N and stress of ~239 MPa. Compared with the untreated condition, hot forging reduced the average grain size from 1746 to 1262 µm near the wall top and from 1053 to 696 µm in the middle region, while the internal void fraction decreased from 1.09% to 0.89%. Synchrotron diffraction revealed only minor phase changes, indicating that the observed effects resulted primarily from hot deformation, recrystallization, texture weakening, and pore collapse. Correspondingly, as shown in Figure 30, horizontal UTS and YS increased from 732 and 453 MPa to 795 and 522 MPa, respectively, whereas vertical UTS remained nearly unchanged at ~715 MPa because the grains were not fully equiaxed and the strengthening effect was less effective along the retained columnar direction, while the vertical YS increased from 443 to 485 MPa. Elongation decreased from 46% to 32% horizontally and from 56% to 38% vertically, indicating a strength-ductility trade-off after hot forging.

#### 3.6.5. Steels

In-situ hybrid AM studies on steels primarily tackled the control of coarse and directionally growing grain structures and heterogeneous transformed microstructures resulting from repeated deposition and reheating cycles. The formation of ferrite, bainite, and martensite was governed by the prior austenite grain size and the cooling history through the austenite-to-ferrite transformation range. Accordingly, in-situ strategies primarily utilized mechanical deformation to refine the prior austenite grain size, increase transformation nucleation sites, weaken texture, and reduce anisotropy.

A wire DED study on ER70S-6 low-carbon steel utilized synchronous hammering for in-situ deformation [53]. Hammering was applied to each bead at frequencies of 21–31 Hz, with temperatures ranging from 850 to 1050 °C and deformations between 20% and 40%. The optimal condition, 850 °C and 40% deformation, reduced the average grain size from ~361 to 30 µm and decreased the maximum texture intensity from 10.1 to 4.66. Since 850 °C is below the austenite recrystallization temperature for these strains, grain refinement was primarily attributed to deformation-induced defects that increased ferrite nucleation sites and nucleation rate, as well as deformation-induced ferrite transformation. At 1050 °C, dynamic recrystallization of austenite occurred under these conditions; however, subsequent austenite grain growth diminished the final ferrite-refinement effect. Yield strength (YS) increased from 297.4 to 529.2 MPa in the build direction and from 282.2 to 506.3 MPa perpendicular to the build direction, while elongation decreased from 42.4% to 37.3% and from 38.3% to 34.2%, respectively.

**Figure 31:**
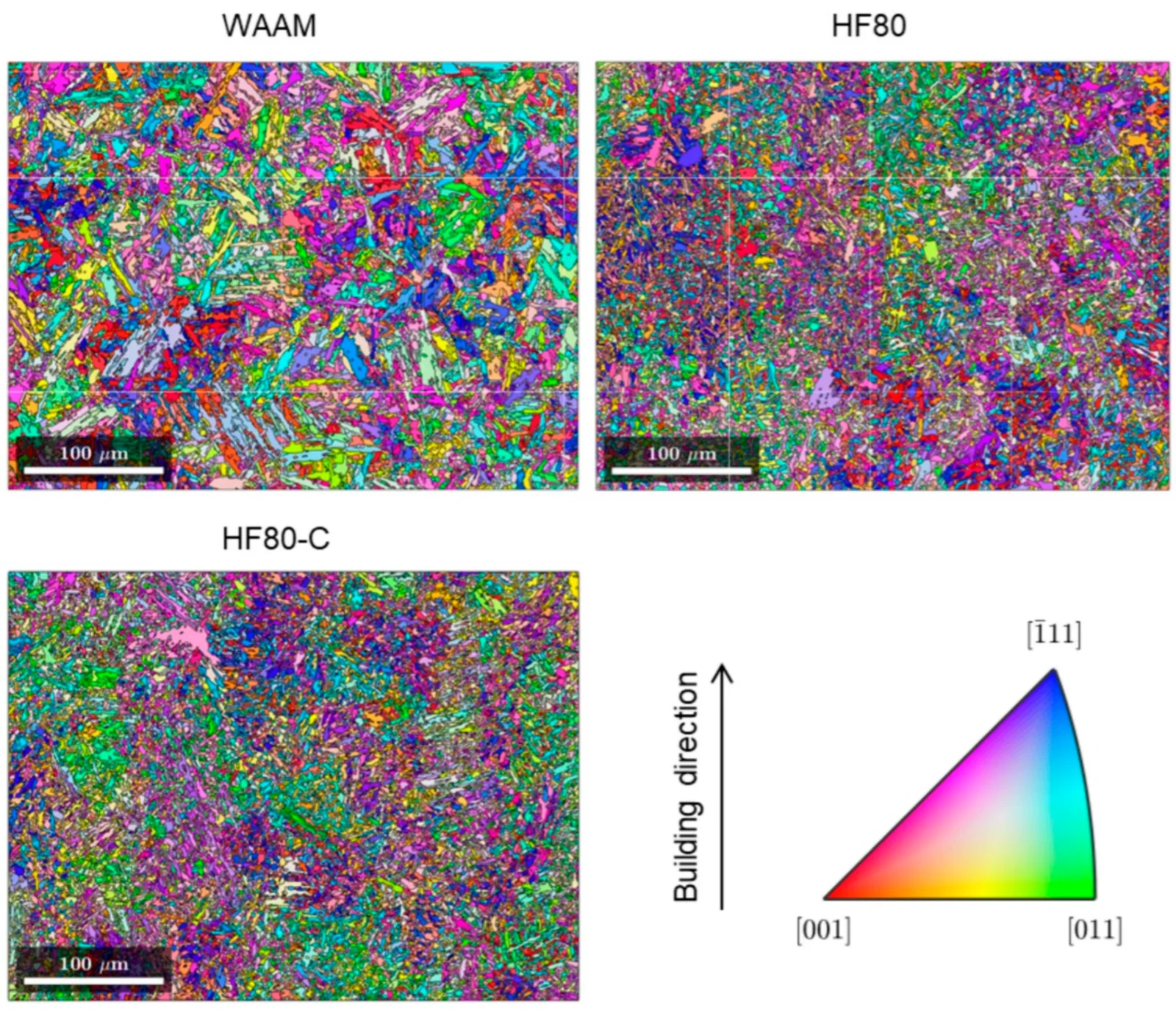
Electron Backscatter Diffraction orientation maps of wire DED ER110S-G in (wire DED) as-deposited, (HF80) in-situ hot forged at 80% duty cycle, and (HF80-C) in-situ hot forged at 80% duty cycle with active cooling conditions [54]—reproduced under Creative Commons (CC BY NC ND) license.

Another wire DED study combined interlayer hot forging with direct cooling to regulate the prior-austenite grain size and transformed microstructure of ER110S-G high-strength low-alloy (HSLA) steel [54]. Forging was applied at 7 Hz and 5 bar, with the hammer operating at duty cycles of 20% or 80%, corresponding to contact with the deposit for 20% or 80% of each hammering cycle. G13 coolant at −25 °C was circulated through the hammer to increase heat extraction. For the 80% duty-cycle condition, the average major-axis length of ellipses fitted to the EBSD-resolved grains in the final transformed microstructure decreased from 2.24 µm in the as-deposited material to 1.66 µm after forging and to 1.55 µm after forging with cooling, with corresponding orientation maps shown in Figure 31. The authors separately inferred that hot forging refined the prior-austenite structure and thereby reduced hardenability. This refinement promoted a greater fraction of softer ferritic constituents, including acicular and grain-boundary ferrite. Consequently, the average hardness decreased from 335 ± 24 HV to 302 ± 28 HV after forging and 281 ± 20 HV after forging with cooling. UTS anisotropy decreased from 1.61% to 0.23% after forging and to 0.57% when cooling was applied as well. Repeated reheating by subsequent layers partially removed the local forging effect, indicating that thermal cycling strongly influenced the final bulk response.

At higher alloying levels, in-situ microrolling was implemented during wire DED of AerMet100 ultra-high-strength steel to fragment the prior austenite structure and reduce mechanical anisotropy [85]. A rolling force of 10 kN was applied synchronously, resulting in irregularly oriented lath-martensite packets rather than a strongly directional columnar structure. UTS, YS, and elongation of the rolled condition were 1280 MPa, 912 MPa, and 14.0% in the deposition direction, and 1277 MPa, 908 MPa, and 15.3% in the build direction, respectively. Following a normalizing heat treatment, high-temperature tempering, quenching, cryogenic treatment, and final tempering at 482 °C, the microstructure contained refined prior-austenite grains of ~40–60 µm, lath martensite, dispersed M_2_C carbides, and film-like reversed austenite, with a low maximum texture intensity of 1.82 MRD. UTS and YS increased to 1747.7 ± 16.3 and 1615 ± 40.6 MPa in the deposition direction and to 1821.3 ± 22.1 and 1624 ± 84.5 MPa in the build direction, respectively. Dispersion of carbides and high dislocation density contributed to strengthening, while film-like reversed austenite deflected and blunted cracks, resulting in mean K_IC_ values of 68.5 and 59.3 MPa√m in the deposition and build directions, respectively.

#### 3.6.6. Special Cases in Metal Alloys

In addition to the major alloy families, very few in-situ hybrid approaches have been studied in dissimilar deposits and specialty alloys such as medium-entropy alloy (MEA) and shape memory alloy (SMA), where limitations were highly localized or chemistry-specific. In such instances, the secondary process served as a targeted intervention to address specific defects, phase instabilities, interfacial constraints, or functional-property requirements, rather than as a general refinement step. The significance here is that the hybrid step was chosen to address chemistry-specific bottlenecks rather than universal DED-related defects.

In Ti-6Al-4V + AlSi_5_ dissimilar wire DED, interlayer ultrasonic peening was applied specifically to the Al alloy region, where hydrogen porosity was the dominant concern. The treatment mechanically collapsed pores from ~70 µm to 10 µm and produced refined equiaxed α-Al grains of ~200–400 nm, along with dislocation walls and tangles [44]. For Inconel 625—HSLA steel functionally graded material (FGM), the critical issue was confined to the 50% Inconel 625—50% HSLA transition region, where the as-deposited material contained Nb/Mo-rich Laves particles measuring ~600 nm and tensile failure consistently occurred. Interrupting the deposition to heat this region at 1080 °C for 1 or 2 h dissolved the Laves phase and promoted the formation of Nb-rich carbides, dislocation tangles, and stacking faults [103]. For AlCrFe_2_Ni_2_ (MEA), a low-power laser heat treatment pass was applied after each deposited layer to promote the solid-state bcc to fcc transformation without the extensive coarsening and softening caused by conventional treatment at 900 °C for 6 h. The short term in-situ thermal cycles increased the fcc fraction from ~40% to 58% through nucleation and thickening of Widmanstätten fcc plates, while retaining hardness. However, this brief exposure did not eliminate bead-scale microstructural heterogeneity [104]. Like the MEA study, for NiTi (SMA), each deposited layer was rescanned, producing a simulated peak temperature of ~1207 °C, followed by transient solution treatment during cooling through ~800–984 °C. This sequence reduced the Ti_2_Ni fraction by ~50–70% and lowered microhardness by about 35 HV [105]. Ultrasonic-assisted wire DED of Invar 36 provides another chemistry-specific instance. Use of 15 kHz vibration treatment converted the periodic columnar structure to predominantly equiaxed grains, reducing the mean grain size from 269 to 51 µm and the maximum ⟨100⟩ texture intensity from 17.55 to 2.33 MUD, resulting in improved tensile properties [88].

#### 3.6.7. Polymers and Composites

Although this review focuses on metal AM, related developments in polymer and composite manufacturing show that hybrid processing is not limited to metals. Ex-situ thermomechanical polymer studies demonstrated the broader principle that secondary processing, especially deformation, can deliberately restructure material morphology. For instance, cross-rolling followed by orientation of high-density polyethylene (HDPE) was reported to increase the tangent modulus from ~1 to 37 GPa and the tensile strength from 51 to 680 MPa at a draw ratio of 11, although the strain at break decreased from 460% to 3.7% [119]. Differential-speed rolling of immiscible HDPE/polypropylene blends increased toughness from 0.74 to 16.2 MJ/m^3^ while reducing density from 0.927 to 0.900 g/cm^3^ through shear-induced domain coalescence, fibrillation, micro and nanovoid formation, and controlled interfacial debonding [120]. These ex-situ studies, while not direct analogs of in-situ AM, support the broader principle that secondary processing can be deliberately used to alter material morphology and mechanical response of non-metals, including those produced via AM processes.

Related in-situ or inline hybrid approaches have also been reported in polymer and composite manufacturing. In thermoplastic composites, localized heating and compaction during in-situ consolidation promoted intimate contact, molecular diffusion across interfaces, control of crystallinity, and void reduction, while also requiring careful thermal control to avoid degradation [121]. Similarly, ultrasound-assisted continuous-fiber composite printing reported improved fiber—matrix wetting and interfacial bonding, increasing tensile and flexural strengths by 34% and 29%, respectively, under optimized conditions in ultrasonic amplitude, resin solution fraction, and processing speed [122].

Together, these representative studies on polymers and composites support a broader view of hybrid processing as a material-state-aware manufacturing strategy. However, the analogy to metal in-situ hybrid AM can only be made at the framework level. In metals, the critical state variables include solidification, defect evolution, dislocation storage, recrystallization, segregation, and phase transformation, whereas in polymers and composites, they include polymer chain mobility, crystallinity, fiber architecture, interlayer bonding, and interface stability. In the case of cross-material expansion, future studies should translate the processing logic rather than the underlying mechanisms.

## 4. Discussion

Ex-situ and in-situ hybrid processing act at different stages in the development of an AM component and are best viewed as complementary. Ex-situ routes are applied after the build, when the solidification structure, residual-stress state, and defect population are already established. They are particularly effective when uniform modification is required across the bulk component or within well-defined local regions and can be utilized on AM preforms in near-net-shape operations. However, the added handling, tooling, energy demand, and geometric constraints associated with downstream processing remain consistent drawbacks. In contrast, in-situ routes intervene before the material state is fully determined. By using retained heat, interlayer access, and subsequent reheating, they can modify defects and microstructure closer to the stage at which they form, often with lower local force or energy. Their effects, however, are more sensitive to timing, treatment depth, and subsequent deposition cycles.

Based on the in-situ hybrid DED literature, effectiveness depended on coupling the secondary process to the appropriate thermal and structural state. The same intervention could produce different outcomes depending on whether it acted on the melt pool, a viscoplastic bead, or a solidified and cooling/cooled layer(s). This was especially evident in deformation-assisted studies, where the final microstructure was governed by the overlap among the deformed zone(s), the region(s) reheated by subsequent deposition, and the portion that was fully remelted. Material nearest the surface could lose its deformation history through remelting, material below the surface could recrystallize, and deeper regions could remain strain hardened. This path dependence is a defining characteristic of in-situ hybrid processing where secondary-process parameters cannot be optimized independently of the material property and primary deposition conditions.

Across the reviewed material families, the most effective hybrid strategies addressed the dominant alloy-specific limitation without compromising other critical properties. In aluminum alloys, porosity was the most persistent concern, but pore closure did not necessarily mean permanent pore healing because later thermal exposure could reopen incompletely bonded cavities without removing the residual dissolved hydrogen. In solid-solution-strengthened Al-Mg alloys, rolling could improve strength through dislocation storage, grain refinement, and pore closure. In precipitation-hardenable Al-Cu and Al-Zn-Mg-Cu alloys, deformation first modified the defect and phase population, but the final properties remained strongly dependent on subsequent solution treatment, aging, and precipitate stability. In stainless steels, the main objective was disruption of columnar austenitic grain growth, texture, and anisotropy. Deformation-assisted approaches were most effective when the strain field and later thermal cycle were coordinated to promote recovery or recrystallization without excessive remelting, grain growth, or formation of harmful secondary phases. Titanium alloys required simultaneous control of the prior-β structure and the transformed α/α′ or α/β morphology. Prior-β refinement and texture weakening could reduce anisotropy but did not by themselves guarantee adequate ductility if continuous grain-boundary α, fine martensitic constituents, or unfavorable variant selection remained. In Nb-bearing nickel-based superalloys, the central concern was segregation of Nb and Mo and the associated formation of Laves phase. Forging, ultrasonic vibration, magnetic-field assistance, and inoculation were useful when they shortened diffusion distances, altered solute redistribution, increased heterogeneous nucleation, or reduced the continuity of interdendritic constituents. Steels required a broader framework for hybrid processing because their properties depended on multiple features, including the prior-austenite grain size, transformation temperature, cooling rate, ferrite, bainite, and martensite formation, carbide precipitation, retained or reversed austenite, and interlayer cleanliness. In these systems, even a reduction in hardness could represent a favorable shift toward a tougher transformed microstructure rather than a loss of performance.

A comparison of ex-situ and in-situ thermomechanical processing of DED Ti-6Al-4V illustrates their common metallurgical basis but different operating scales. In the ex-situ hybrid route, DED Ti-6Al-4V was forged at approximately 930 °C to 50% reduction and then stress-relief annealed at 710 °C for 6 h [30]. The large imparted bulk strain substantially changed the characteristic DED morphology, producing partially globularized α grains of approximately 2.3 µm, about 9% β phase, and nearly orientation-independent tensile properties. UTS, YS, and elongation reached 912–916 MPa, 838–844 MPa, and 13.1–14.5%, respectively. In the in-situ hybrid route, interpass forging interrupted the vertical continuity of the prior-β structure, reducing its span from three or four deposited layers to one or two layers [60]. Subsequent treatment at 850 °C for 2 h decomposed the acicular α′ constituent and modified the α/β morphology, producing elongation of ~13–14% while retaining a higher UTS of ~980–1000 MPa. Although these studies were not a controlled head-to-head comparison, they highlight an important distinction. Ex-situ forging used large bulk deformation to reconstruct the AM microstructure throughout the preform, whereas in-situ forging used repeated local deformation and the deposition thermal cycle to interrupt prior-β inheritance before the structure became fully established. The ex-situ hybrid route provided greater bulk uniformity and geometric forming capability. The in-situ hybrid route retained more of the AM strength and required smaller loads applied locally, but its response was more dependent on treatment depth, layer sequence, and subsequent reheating. The in-situ hybrid approach also still benefited from post-deposition heat treatment to obtain the desired α/β morphology.

A similar relationship was observed in stainless steels. In ex-situ hybrid processing of wire DED 304L, warm rolling to 24.9–41.1% true strain followed by heat treatment at 850–1050 °C removed much of the columnar structure, crystallographic texture, and elastic anisotropy that heat treatment alone could not eliminate [31]. After 41.1% rolling and recrystallization at 850 °C, the vertical and longitudinal elastic moduli converged to 190–193 GPa, while UTS and elongation reached 641–652 MPa and 54–67%, respectively. By comparison, in-situ rolling of wire DED 316L at 8 kN and approximately 810–830 °C introduced a deformation structure that became highly responsive to later thermal exposure [83]. Heat treatment at 1000 °C produced equiaxed austenite grains of ~22 µm in the rolled material, while treatment at 1200 °C produced grains of ~45 µm, compared with approximately 180 µm in the non-rolled condition. The two stainless steel hybrid routes relied on the same general sequence of deformation/strain storage followed by thermally activated recrystallization but differed in when, where, and how the deformation/strain was introduced. The ex-situ hybrid route imposed sufficient bulk deformation/strain to produce section-wide recrystallization and nearly eliminate orientation-dependent properties. The in-situ hybrid route created a refined microstructure through lower-force interlayer deformation, but remained sensitive to the subsequent thermal path, including undesirable σ-phase formation after treatment at 650 °C. These examples emphasize that in-situ hybrid processing does not eliminate the need for conventional thermomechanical process metallurgy; rather, it distributes those metallurgical principles/changes across a sequence of short, spatially varying, and recurring deformation and thermal events. The relative effectiveness of ex-situ and in-situ processing is application-dependent rather than universally rankable; their principal differences in processing stage, material-state interaction, capabilities, and limitations are summarized in Table 8.

Specialty alloys, dissimilar systems, and functionally graded materials further showed that the value of process hybridization extends beyond general grain refinement. In these systems, the main benefit was site-specific metallurgical control, such as stabilizing a dissimilar-material interface, strengthening a vulnerable graded transition, adjusting local phase balance, reducing harmful secondary phases, or preserving functional behavior. Such applications are particularly attractive for in-situ hybrid processing because global post-processing may be inefficient, may alter regions that already possess an acceptable microstructure, or may be unable to selectively correct a narrow transition zone.

Industrial interest in hybrid AM is reflected in both full-scale demonstrations and patent activities spanning ex-situ and in-situ process integration. For instance, in an early aerospace demonstration, a near-net-shape Ti-6Al-4V forging preform was produced by laser deposition of a rib onto a Ti-6Al-4V plate and subsequently subjected to HIP, heat treatment, and closed-die forging [26]. The targeted MD-11 airframe structural component conventionally required four forging operations, whereas the laser-deposited preform was designed to reach the final configuration in a single forging operation, with substantial β-grain refinement to satisfy the targeted mechanical-property requirements. The projected production cost remained ~35% higher than that of the conventional route, demonstrating the feasibility of DED in industrial applications, while also reporting that reductions in forging steps do not necessarily provide an overall economic advantage without optimization of the complete process chain. This trend is also evident in commercial offerings such as the TRUMPF TruLaser Cell 3000, which integrates DED platform with laser cutting and welding within a unified system [123].

Relevant industrial patents further demonstrate that both ex-situ and in-situ hybrid concepts are considered for high-value structural components. A Howmet/Arconic patent disclosed a workflow in which an AM preform is first produced and then converted into a final forged or otherwise worked product, with optional annealing after forming [124]. The disclosed post-AM hybrid routes include forging, rolling, ring rolling, ring forging, shaped rolling, extrusion, and combinations of these operations. This reflects the industrial logic of ex-situ thermomechanical processing, where AM provides a material-efficient preform, while downstream working provides final shape and microstructural control. A United Technologies/Raytheon patent application for additively manufactured superalloy turbine components describes a more extensive ex-situ processing chain [125]. In that disclosure, a turbine component containing internal cooling passages is produced by AM and then subjected to HIP, directional recrystallization, bond-coat application, thermal-barrier coating, and final precipitation heat treatment. This route illustrates the extent of ex-situ hybrid processing combinations to manufacture high-temperature aerospace components. In contrast, a more recent General Electric patent describes a forming system in which AM is combined with sequential micro-forging tools that deform the material during or immediately after solidification, followed by further forming as the material cools [126]. This disclosure approaches the in-situ hybrid-processing regime and suggests a shift from global post-build correction toward localized deformation coupled more closely to deposition. While patent disclosures do not by themselves establish production readiness or qualified performance, they show industrial interest across the full integration spectrum.

## 5. Future Directions

Future work should move hybrid AM beyond isolated proof-of-concept studies toward integrated, repeatable, and application-specific manufacturing routes. This requires different priorities for ex-situ and in-situ hybrid processing, as well as the desired properties to be modified/optimized. For ex-situ hybrid approaches, emphasis should be placed on defect-aware, sequence-optimized process chains rather than isolated post-processing steps. This requires defining thermomechanical conditions that achieve stable defect control while tailoring the microstructure for desired properties without compromising other properties. Multiaxial deformation and optimized sequencing of forging or rolling, HIP, heat treatment, and machining should, therefore, be explored to achieve the desired final material state. For in-situ hybrid approaches, the main priority is to replace fixed secondary-process settings with control based on the evolving material state. Interventions should be applied within a defined processing window rather than at a fixed distance or delay that may no longer remain valid as component size and geometric complexity increase. As indicated by emerging industrial activity and shifts in the academic literature [52,73,126], multi-process approaches represent a logical extension of this strategy because different interventions can act at different stages of the deposition cycle. Mechanistic claims, therefore, require appropriate controls for each individual intervention and for the combined process. Attention should also be given to process interactions, for instance, whether magnetic stirring alters bead geometry and the effective rolling reduction, or whether active cooling preserves stored deformation while reducing the time available for recrystallization.

Progress in in-situ hybrid approaches will also depend on the invention/generation of purpose-built machines at different scales. Most existing hybrid systems add a secondary tool to equipment originally designed only for deposition. Future hybrid machines should instead provide mechanically stiff and independently controlled paths for deposition and secondary processing, with unified sensing and machining coordinate systems. More importantly, the equipment must provide collision-free access to three-dimensional deposited geometries for all subsystems. In many setups, thermal protection, active heating and cooling, lubricant management, tool-life monitoring, automated cleaning, and rapid recalibration will also be crucial for reliable long-duration builds. Because existing studies have shown that simplified single-process simulations may be insufficient [127,128,129], reliable models must be developed alongside these hybrid machines. A coupled wire DED—rolling simulation demonstrated that deposition and rolling must be modeled together to capture the repeated regeneration and redistribution of plastic strain and residual stress. Flat interlayer rolling reduced the longitudinal tensile residual stress in an underlying layer from 550 to 201 MPa, whereas a slotted roller reduced comparable stress from 500 to 3 MPa. Stacked-four-layer rolling using a slotted roller also achieved similar residual-stress mitigation with fewer rolling operations [127]. Multi-process simulations of ultrasonic-assisted rolling in wire DED of a Pb-Sn alloy linked melting, solidification, dynamic recrystallization, and grain growth, showing a relative error of 4.33% compared with experiment and predicting an average grain size of 18.8 µm, 23.23% lower than the non-rolled condition, at a 20 µm ultrasonic amplitude and 24 mm tool offset [128]. With robust models, the next step will be to convert them from post-build interpretation tools into reduced-order models that support near-real-time decisions. This will require synchronized sensing of the hybrid machine, process, and material-state variables, together with uncertainty quantification. The Agility Forge platform provides a useful forward-looking framework in which thermal and geometry sensing, load–stroke data, finite-element simulation, microstructure prediction, machine learning, and a digital twin are combined to select and update thermomechanical paths [130].

Future studies must also move beyond hardness and monotonic tensile mechanical property measurements. This is crucial for in-situ hybrid approaches, where many studies have demonstrated favorable changes, but after more than a decade of research, relatively few have connected these changes to service-relevant fracture-critical behavior such as fatigue, fracture toughness, corrosion resistance, cryogenic impact toughness, high-temperature tensile response, or direction-dependent mechanical performance. This gap is particularly important for widely studied engineering alloys such as Al 7075, 316L stainless steel, Ti-6Al-4V, and IN718, where microstructural refinement alone does not ensure reliable performance in demanding applications. In many cases, an in-situ hybrid intervention may improve one material response while compromising another, such as enhanced grain refinement accompanied by reduced ductility. Therefore, the most impactful studies are those that move beyond microstructural refinement alone and establish clear process–structure–property–performance (PSPP) relationships, showing how the secondary-process mechanism affects application-relevant material behavior.

A broader conclusion from the reviewed literature is that process innovation has often advanced faster than property validation, consistent with a general trend in additive manufacturing [1,11,12]. A crucial step is, therefore, standardized reporting of the complete process-structure-property-performance record. Detailed documentation should include machine configuration, tool geometry and contact area, force or displacement history, processing temperature, tool-to-heat-source offset, interpass delay, deformation and remelting depths, shielding and cooling conditions, toolpath, and measured process signals. These data should be linked to spatially resolved measurements of defects, grain structure, texture, phase distribution, residual stress, and properties and, where possible, deposited in open-access databases. The value of such structured datasets is demonstrated by recent work on PBF Ti-6Al-4V, in which processing parameters for 175 fatigue specimens were linked with fatigue life and fracture-based characterization of fatigue-initiating defects, including defect type, size, shape, and location relative to the free surface [131]. Extending this approach to hybrid AM will require additional documentation of secondary-process parameters, processing sequence, and evolving material-state variables so that the combined effects of primary and secondary processing can be related to final performance. Appropriate untreated, single-process, and conventionally post-processed controls are also needed, together with sufficient replication to quantify process variability. Consistent reporting will support cross-study comparison, model calibration, process transferability, nondestructive-evaluation development, and future part qualification.

Confidence in hybrid AM will also require demonstrations using industry-relevant materials and challenge parts with mechanical property goals/metrics, rather than only straight walls and extracted coupons. A useful challenge part should combine realistic scale, geometric transitions, constrained tool access, multiple deposition orientations, stress concentrations, and representative loading conditions. A pickle-fork benchmarking study provided one such instance, evaluating wire DED, wire-laser DED, powder DED, PBF, additive friction stir deposition (AFSD), and Agility Forged materials through a common workflow of preform fabrication, structured-light scanning, finish machining, and mechanical testing [132]. PBF required only ~1 mm of overbuild, whereas wire DED and AFSD generally required 2–3 mm of machining allowance. Even after machining to a common geometry, performance remained process dependent. In preliminary part-level fatigue testing, the as-deposited/machined Al wire DED and AFSD parts failed after 2040 and 1054 cycles, respectively, compared with 8530 cycles for wrought Al 6061, while 316L wire-laser DED reached approximately 9 × 10^5^ cycles compared with 4.7 × 10^5^ cycles for wrought 316L. Heat treatment of the Al-based samples increased both their hardness and monotonic properties, but fatigue performance was still lacking due to AM process-induced defects. Continuing work is examining hybrid approaches to improve a range of mechanical properties. These results demonstrate why part-level validation must account for geometry, defects, surface condition, and loading path rather than relying only on coupon-scale tensile data.

Industrial adoption will ultimately depend on demonstrated value. In line with a detailed techno-economic analysis (TEA), hybrid routes should be benchmarked against both the as-deposited condition and established post-processing routes using common measures of performance, cycle time, energy consumption, material utilization, machining allowance, tooling requirements, maintenance, repeatability, and total cost. Preliminary efforts include recent studies within the NSF ERC HAMMER program [133,134]. However, while a hybrid process may not outperform conventional processing in every category, it must provide a clear advantage in at least one critical metric without introducing unacceptable trade-offs elsewhere. The long-term objective is to achieve repeatable and certifiable material states with performance comparable to conventionally processed components while retaining the geometric flexibility, local control, and process-integration advantages of AM.

## 6. Conclusions

Hybrid processing provides a unified framework for tailoring the local or global microstructure, defect population, residual stress, and performance of metal AM components. Ex-situ hybrid routes, including heat treatment, HIP, thermomechanical processing, machining, and surface treatment, remain effective for bulk densification, homogenization, phase control, and surface improvement, but act only after the AM microstructure and defects have formed and may add geometric constraints, handling, cost, and energy demand. In-situ hybrid routes instead use the evolving liquid, viscoplastic, or solidified material state to modify defects and microstructure during fabrication, often with lower local force or energy. Their effectiveness, however, depends strongly on the timing, treatment depth, alloy response, and interaction with subsequent remelting and reheating. Across both approaches, the most successful strategies were alloy-specific and property-specific and often combined complementary processes rather than relying on a single intervention. Hybrid processing should, therefore, be designed as part of the overall manufacturing route, rather than added as a separate step. Broader adoption will require repeatable processing windows, mechanistic validation, geometry-aware control, and demonstration of fatigue, fracture, corrosion, and other service-relevant performance in representative industry-relevant components.

## Figures and Tables

**Table 1:** Major metal AM defects and typical consequences.

Defect/Heterogeneity	AM Origin	Consequence
Porosity/lack of fusion	Gas entrapment, insufficient fusion, interlayer discontinuities	Reduced fatigue/fracture resistance
Residual stress/distortion	Steep thermal gradients and cyclic heating	Warping, cracking, dimensional error
Columnar grains/strong texture	Directional heat flow and epitaxial growth	Mechanical anisotropy
Elemental segregation/ undesirable phases	Rapid solidification and interdendritic partitioning	Brittle phases, reduced ductility/strength
Roughness/surface-connected defects	Layer-wise deposition and partially fused material	Fatigue crack initiation, poor dimensional accuracy
Oxides/inclusions/slag	Surface contamination or arc-process by-products	Weak bonding and crack initiation

**Table 2:** Representative ex-situ processing routes for AM metallic preforms.

Secondary Process	Source	AM ^1^	Alloy	Primary Focus
Heat Treatment (HT)	[17]	PBF	Ti-6Al-4V	Martensite decomposition, phase morphology, ductility
	[18]	PBF	IN718	AM-specific heat treatment, precipitation, Laves/δ control, strength-ductility
	[19]	BJ	IN625	Solutionizing/aging, precipitation, phase evolution, mechanical properties
Hot Isostatic Pressing (HIP)	[20]	PBF	Ti-6Al-4V	Internal pore closure by XCT, surface-connected defect limitation
	[21]	ME	IN625	Defect-density reduction, microstructure, tensile ductility
	[22]	BJ	Ti-6Al-4V	Densification, porosity, mechanical/fatigue properties
	[23]	PBF	Ti-6Al-4V	Porosity closure, prior-β grain morphology, strength retention
HIP + HT	[24]	PBF	IN718	Porosity removal, precipitation homogenization, tensile-property uniformity
HIP + Forging + HT	[25]	DED	Ti-6Al-4V	Porosity closure, α globularization, recrystallized β-grains
	[8]	PBF	AlSi10Mg	Recrystallization, defect modification, strength and ductility
	[26]	DED	Ti-6Al-4V	Grain refinement, qualification, and process-chain study
Warm Isostatic Pressing	[27]	ME	316L SS	Pore reduction, tensile strength, strain response
Forging	[28]	DED + PBF	Ti-6Al-4V	Hot deformation behavior, flow stress, α globularization
Forging + HT	[29]	DED	IN718	Hot workability, Laves dissolution, DRX, flow behavior
	[30]	DED	Ti-6Al-4V	Grain refinement, α globularization, anisotropy reduction
Rolling + HT	[31]	DED	304L SS	Texture reduction, recrystallization, mechanical anisotropy
Peening + chemical surface treatment	[32]	PBF	Ti-6Al-4V	Fatigue performance, surface roughness, residual stress
Machining	[33]	PBF	Ti-6Al-4V	Surface roughness reduction, fatigue improvement
Machining + HIP	[34]	PBF	GRX-810	Fatigue resistance, volumetric defects, crack initiation
Friction Stir Processing (FSP)	[35]	PBF	AlSi10Mg	Pore elimination, grain refinement, densification, ductility

^1^ AM types were grouped using broad categories. DED, directed energy deposition, includes wire and powder-fed deposition routes. PBF, powder bed fusion, includes laser and electron-beam routes. ME, material extrusion, includes all metal-filled filament-based routes followed by debinding and sintering. BJ, binder jetting, refers to powder-bed binder jet printing followed by sintering. When a study includes more than one AM route, both process families were listed.

**Table 3:** Classification of in-situ hybrid DED based on secondary processing.

Secondary Process	Included Type	DED Type	RX Response ^1^	Alloys Explored
Compression (Forging + Peening)	Hammering; deformation; forging; shock-forging; micro-forging; hammer-forging; forming; compression; peening; impact treatment; machine hammer peening; localized compression.	Wire DED = #20 [43,44,45,46,47,48,49,50,51,52,53,54,55,56,57,58,59,60,61,62] Powder DED = #7 [63,64,65,66,67,68,69]	SRX = #13 [45,46,50,51,52,55,60,62,64,65,66,67,68] DRX = #10 [43,47,49,53,54,56,57,58,59,61] No RX evidence = #4 [44,48,63,69] RX reported = 85%	Al; Ni-based; stainless steel; steel; Ti. Most explored: 316L [56,58,59,66,67,69]
Rolling	Rolling; micro-rolling; side rolling; vertical rolling; pinch rolling; inline rolling/forming.	Wire DED = #19 [52,61,70,71,72,73,74,75,76,77,78,79,80,81,82,83,84,85,86]	SRX = #10 [52,71,73,75,76,78,79,81,83,86] DRX = #5 [61,70,72,82,84] No RX evidence = #4 [74,77,80,85] RX reported = 79%	Al; stainless steel; steel; Ti. Most explored: Al 5087 [72,73,74,75,77,84]
Ultrasonic Vibration	Ultrasonic vibration; power ultrasound; ultrasonic assistance.	Wire DED = #2 [87,88] Powder DED = #4 [89,90,91,92]	SRX = #1 [87] No RX evidence = #5 [88,89,90,91,92] RX reported = 17%	Fe-Ni; Ni-based; stainless steel; Ti. Most explored: IN718 [89,91]
Friction Stir Processing	Interlayer FSP; unified additive-deformation; high-speed friction treatment.	Wire DED = #5 [93,94,95,96,97]	DRX = #4 [93,94,96,97] No RX evidence = #1 [95] RX reported = 80%	Al; stainless steel. Most explored: 4043 Al [94,97]
Alloying	Inoculation; reinforcement; particle addition; interlayer coating; coating assisted control; nanoparticle assistance.	Wire DED = #4 [98,99,100,101]	No RX reported	Al; Ni-based; steel; Ti.
Multi-process Hybrid	Magnetic-field assisted hot rolling; arc vibration shock forging rolling.	Wire DED = #2 [52,73]	SRX = #2 [52,73] RX reported = 100%	Al; steel.
Stirring	Ultrasonic molten pool stirring.	Wire DED = #1 [102]	No RX reported	7075 Al matrix nanocomposite
Heat Treatment	Intrinsic heat treatment; selective local heat treatment.	Wire DED = #1 [103] Powder DED = #2 [104,105]	No RX reported	Ni-based; medium entropy alloy; shape memory alloy.
Magnetic Field	Static magnetic field; alternating magnetic field; magnetic field combined with rolling.	Wire DED = #1 [73] Powder DED = #2 [106,107]	SRX = #1 [73] No RX evidence = #2 [106,107] RX reported = 33%	Al alloys; Ni-based superalloys; Ti alloys.
Surface Preparation (Machining + Cleaning)	Machining; milling; slag removal; surface cleaning;	Wire DED = #3 [108,109,110]	No RX reported	Al alloys; ER100S-G steel.

^1^ For recrystallization (RX) response, studies were classified as follows: SRX (static recrystallization) was assigned when prior deformation stored strain energy and recrystallization occurred later during reheating, subsequent deposition, or post-thermal exposure, including post-build heat treatment. DRX (dynamic recrystallization) was assigned when recrystallization occurred during active hot deformation, where deformation and thermal activation overlapped. No RX evidence was assigned when globularization, phase transformation, or deformation was reported without strong claims of recrystallization. For transformation-assisted β recrystallization in Ti alloys, the response was classified as SRX or DRX based on timing of the recrystallization claims.

**Table 4:** Mechanistic pathways and outcomes based on secondary processing in in-situ hybrid DED.

Secondary Process	Deposition State Interaction	Metallurgical Response	Typical Outcomes
Deformation (Compression + Rolling)	Solidified or viscoplastic layer	Plastic deformation, stored strain energy, dislocation generation	Subgrain formation, pore closure, texture weakening, residual-stress redistribution, recovery or recrystallization, grain refinement; improved strength, ductility, hardness, fatigue resistance, and impact toughness
Friction Stir Processing	Solidified layer	Severe plastic deformation, frictional heating, mechanical stirring	Dynamic recrystallization, pore closure, oxide disruption, local homogenization, grain refinement, improved bonding; enhanced strength-ductility balance, and reduced anisotropy
Ultrasonic Vibration and Stirring	Melt pool or partially solidified region	Acoustic streaming, cavitation, forced convection, dendrite fragmentation, melt-pool homogenization	Grain refinement, reduced porosity, improved bead morphology, reduced cracking tendency, modified secondary phases; improved hardness and tensile response
Alloying	Melt pool	Heterogeneous nucleation, solute redistribution, particle dispersion, dispersion strengthening	Grain refinement, reduced segregation, modified secondary phases, improved particle distribution; higher hardness, strength, wear resistance, and hot-cracking resistance
Magnetic Field	Arc plasma and melt pool	Lorentz-force effects, modified convection, solute redistribution, thermal-gradient modification	Texture modification, grain refinement, reduced segregation, improved melt-pool stability, microstructural uniformity, increased ductility, and defect reduction
Heat Treatment	Solidified layer	Diffusion activation, recovery, phase dissolution, precipitation	Phase control and transformation, precipitate modification, adjusted hardness; improved strength-ductility balance, corrosion response, and functional property control
Surface Conditioning	Solidified layer	Surface cleaning, surface finish	Reduced inclusions and interlayer defects, improved bonding, reduced surface roughness, increased toughness, and fatigue performance
Multi-process Hybrid	Melt pool, solidified or viscoplastic layer	Coupled thermal, mechanical, acoustic, chemical, or electromagnetic mechanisms	Melt-pool flow control, pore closure, dendrite fragmentation, recrystallization, phase modification, residual stress redistribution, coordinated grain control, texture weakening, and improved mechanical response

**Table 5:** Representative porosity evolution in in-situ hybrid processing of aluminum alloys.

Study	Methodology ^1^	As-Deposited Data	After In-Situ Data	Key Takeaway
[93] Al 2319 + Friction Stir Processing	OM	Porosity ~1.7%	Pores undetectable by optical microscopy	No pores detected in FSP processed region
[46] Al 2319 + Interlayer Hammering	XCT	Pore volume 0.46 mm^3^; pores concentrated near fusion lines	With 50.8% deformation, total pore volume 0.12 mm^3^	Reduced pore volume by ~74%.
[73] Al 5087 + Magnetic Field + Hot Rolling	OM + ImageJ binarization	Average porosity 0.76%; pores concentrated near fusion lines	0.2 T MF: 0.40%; 0.4 T MF: 0.52%; 0.2 T MF + HR: ~0.23% using 4 kN, ~20% deformation, ~400 °C rolling	0.2 T field + hot rolling gave the lowest porosity, reducing from 0.76% to ~ 0.23%.
[74] Al 2319/Al 5087 + Rolling + Post Heat Treatment	SR-XRT + 3D pore reconstruction	Al 2319: 10.72 × 10^3^ pores/mm^3^, vol. fraction 1.08%, mean dia. 8.82 µm, sphericity 0.64. Al 5087: 56.10 × 10^3^ pores/mm^3^, 1.06%, 5.38 µm, 0.69	45 kN rolling: Al 2319 = 10.46 × 10^3^ pores/mm^3^, vol. fraction 0.05%, mean dia. 4.01 µm, sphericity 0.55 45 kN rolling: Al 5087 = 13.63 × 10^3^ pores/mm^3^, 0.07%, 4.13 µm, 0.50 45 kN rolling + post HT: Al 2319 = 53.44 × 10^3^ pores/mm^3^, 0.51%, 5.30 µm, 0.70 45 kN rolling + post HT: Al 5087 = 80.50 × 10^3^ pores/mm^3^, 0.74%, 5.31 µm, 0.74	Rolling reduced pore volume fraction, but subsequent heat treatment partially regenerated fine spherical pores.
[77] Al 2319/Al 5087 + Rolling + Post Heat Treatment	OM; pores > 5 µm over 120 mm^2^	Al 2319: 614 pores > 5 µm, mean diameter 13.5 µm, area fraction 0.176%, sphericity 0.74. Al 5087: 454 pores > 5 µm, 25.1 µm, area 0.232%, 0.74. Post-deposition HT alone increased pore area to 0.657% for 2319 and 0.365% for 5087	30 kN rolling: Al 2319 = 5 pores, mean diameter 8.8 µm, area fraction 0.005% 30 kN rolling: 5087 = 11 pores, 9.6 µm, 0.007% 45 kN rolling: no pores >5 µm optically observed. 45 kN rolling + post HT: no pores >5 µm re-initiated/ observed optically	45 kN rolling eliminated pores >5 µm, and prior rolling prevented large pores from re-initiation after heat treatment.
[50] Al-Mg + Hammer Forging	3D X-ray µCT	26,198 pores; porosity 0.0658%; mean equiv. diameter 1.60 µm; mean Area3d 11.77 µm^2^; mean Volume3d 5.80 µm^3^	~80 N forging force: 8297 pores; porosity 0.0065%; mean equivalent diameter 1.38 µm; mean Area3d 6.03 µm^2^; mean Volume3d 1.90 µm^3^.	Reductions: pore number 68.33%, porosity 90.12%, equivalent diameter 13.75%, surface area 48.77%, volume 67.24%
[94] Al 4043 + Friction Stir Processing	OM; 4 × 6 mm^2^ area	Average porosity ~1.2%; maximum pore size ~567 µm;	Pores undetectable by optical microscopy	Removed large visible pores from the effective processed region.
[84] Al 5087 + Hot Rolling	OM + ImageJ binarization	Average pore diameter 47.3 µm; porosity 1.89%	Average pore diameter 36.1 µm; porosity 0.56%; largest pore <120 µm.	Reductions: average pore diameter 23.7%, porosity 70.4%

^1^ OM, optical microscopy; XCT, X-ray computed tomography; XRT, X-ray tomography; SR-XRT, synchrotron X-ray tomography; µCT, micro-computed tomography.

**Table 6:** Representative microstructural evolution in in-situ hybrid processing of stainless steel alloys.

Study	Methodology ^1^	As-Deposited Data	After In-Situ Data	Key Takeaway
[90] 17-4 PH + Ultrasonic Vibration	SEM	Columnar grains; average grain size ≈7–11 µm.	Equiaxed grains ≈1.5–3 µm.	Columnar-to-equiaxed refinement with fewer solidification defects.
[47] 321 + Ultrasonic Impact Treatment	OM; XRD; EBSD	44.6 ± 4.8 µm upper region; 48.6 ± 5.7 µm lower region.	15.4 ± 3.2 µm upper region; 13.4 ± 2.8 µm lower region.	Finer equiaxed dendrites with reduced texture/ dislocation heterogeneity.
[66] 316L + Forging	SEM; EDS; XRD; EBSD; TEM	Average grain size ≈55 µm; maximum grain size >≈280 µm.	Average grain size ≈17 µm; maximum grain size <≈120 µm.	Strong grain refinement with texture weakening.
[67] 316L + Peening	OM; SEM; EBSD	Largely columnar grains with <100> texture; no single full-section average grain size reported.	Low deformation: no clear recrystallized zone. Moderate deformation: ≈100 µm recrystallized zone. High deformation: ≈450 µm recrystallized zone; recrystallized-zone grain size 12.8 ± 2.4 µm.	Deformation-dependent recrystallized bands over retained deformation zones.
[51] 347 + Cold Forging	OM; SEM; EDS	Large dendritic/cast-like structure; no whole-wall average grain size reported.	Recrystallized grain diameter: AR 11 µm, BR 9.2 µm, CR 8.2 µm, DR 4.0 µm. Repeated-cycle region: 8.2 to 4.8 µm.	Reheating-driven recrystallization; higher cold work gave finer grains.
[95] 307Si + High Speed Friction	OM; EBSD	Maximum grain dia.: top ≈660 µm, middle ≈420 µm, bottom ≈460 µm.	Maximum grain diameter: top ≈580 µm, middle ≈320 µm, bottom ≈420 µm.	Partial grain refinement with disrupted columnar growth direction.
[56] 316L + Hot Forging	OM; Synchrotron XRD	PDAS ≈32 µm; SDAS ≈12 µm.	PDAS ≈13–15 µm; SDAS ≈7–8 µm, depending on force/area.	Refined dendrite arm spacing with weaker solidification texture.
[82] 316L + Hot Rolling	EBSD	Average grain size in deposited layer ≈143 µm and interface ≈102 µm.	At 950 °C: deposited layer ≈68 µm at 15% strain and ≈65 µm at 25% strain; interface ≈38 µm at 25% strain. Multi-pass repaired layer ≈14 µm.	DRX/SRX-assisted refinement of deposited layer and interface.
[68] 304 + Ultrasonic Impact Treatment	OM; XRD; SEM; EBSD; TEM	EBSD maps contained 92 and 97 grains at top/ middle locations; coarse columnar grains dominated. Ferrite fraction ≈13.15%.	Grain number increased by ≈180% on average; finer equiaxed grain-gradient structure formed. Ferrite fraction ≈13.78%.	Coarse-columnar to equiaxed grain-gradient structure.
[83] 316L + Rolling + Post Heat Treatment	OM; BSE; EDS; EBSD	Elongated columnar austenite grains up to several millimeters	1000 °C HT: equiaxed grains ≈22 µm. 1200 °C HT: grains ≈45 µm. Non-rolled + 1200 °C HT: recrystallized grains ≈180 µm.	Rolling-enabled recrystallization; σ phase formed at 650 °C.
[58] 316L + Hot Forging	OM; EBSD	Coarse columnar grains; average grain size ≈158.6 µm; strong <100> {200} build-related texture.	Fine equiaxed grains ≈45 µm overall; strongest refinement at medium heat input.	Columnar-to-equiaxed refinement.
[59] 316L + Hot Forging with Interlayer Cooling	OM; EBSD	Average grain width ≈0.180–0.190 mm.	Best refined condition: average grain width ≈0.038 mm upper region and ≈0.020 mm lower region.	Strong grain-width reduction with fine/ equiaxed grain formation.

^1^ OM, optical microscopy; SEM, scanning electron microscopy; BSE, backscattered electron imaging; EDS, energy-dispersive X-ray spectroscopy; XRD, X-ray diffraction; EBSD, electron backscatter diffraction; and TEM, transmission electron microscopy.

**Table 7:** Representative microstructural evolution in in-situ hybrid processing of titanium alloys.

Study	Methodology ^1^	As-Deposited Data	After In-Situ Data	Key Takeaway
[45] Ti-6Al-4V + Ultrasonic Impact Peening	EBSD	Columnar β grains, ~1–2 cm; strong ⟨001⟩ texture	β grains ~1.2 mm in refined center; texture ~5.84 MRD; strain ~6–9% to ~0.45 mm depth	Refined β grains localized near peened center region.
[70] Ti-6Al-4V + Hybrid In-situ Micro-rolling	TEM; EBSD	α laths ~3–6 µm, commonly ~4 µm; dislocation density ~8.1 × 10^16^ m^−2^	α laths ~8–11 µm, commonly ~10 µm; dislocation density ~10.0 × 10^16^ m^−2^	Higher dislocation density with coarser α laths.
[63] Ti-6Al-4V + Ultrasonic Impact Peening + Post Heat Treatment	OM; SEM; TEM	Columnar prior-β grains, 100–400 µm wide; α/α′ laths ~570 nm; β laths ~58 nm	Fine equiaxed prior-β grains; primary α 76.7%; α size 9.9 µm; aspect ratio ~1.5 after HT	Fine equiaxed prior-β plus globular α after HT.
[99] CP Ti + Si In-situ Alloying	OM	Grain density ~0.7 grains/mm^2^ at ~0.04 wt.% Si; prior-β size ~1.1–1.2 mm	Grain density ~1.6 grains/mm^2^ at 0.19 wt.% Si and ~3.1 grains/mm^2^ at 0.75 wt.% Si; grain size ~0.55–0.60 mm at 0.75 wt.% Si	Narrower columnar grains with partial equiaxed nucleation.
[79] Ti-6Al-4V + Interpass Rolling	OM; EBSD	Large columnar prior-β grains; strong α/β texture	Refined prior-β grains; 75 kN refined to <100 µm; 50 kN was coarser and less homogeneous	Strong β refinement with weakened texture.
[92] Ti-6Al-4V + Ultrasonic Vibration	OM; SEM; EBSD; XRD	Columnar grains 200–500 µm; α′ needles 50–100 µm	Equiaxed regions 10–100 µm, often 70–100 µm; α′ needles 2–10 µm	Local equiaxed regions and finer α′ needles.
[55] Ti-6Al-4V + Machine Hammer Peening	OM; EBSD	Large prior-β grains; several-centimeter growth through wall	Refined depth ~3 mm for top peening; almost ~2 mm for side peening; exact β-grain size not reported	β refinement over limited peened depth.
[81] Ti-6Al-4V + Interpass Rolling	OM; EBSD	Thick-wall columnar prior-β grains	Mean prior-β grain area reduced to ~0.39 mm^2^ at 60 kN/R1, ~0.26 mm^2^ at 60 kN/R3, and ~0.17–0.23 mm^2^ at 90 kN	β refinement despite thick-wall restraint.
[107] Ti-6Al-4V + Static Magnetic Field	OM; XRD; SEM; EBSD; TEM	Prior-β grain width ~0.91 mm; strong ⟨001⟩ texture; continuous α grain-boundary films	Prior-β grain width ~0.57 mm at 0.55 T; weaker ⟨110⟩ texture; discontinuous α grain boundaries	Narrower/twisted β grains; discontinuous α grain boundaries.
[62] Ti6242 + Hammer Peening	OM; SEM/EDS; EBSD	Coarse columnar prior-β grains ~mm in height; maximum β-texture intensity 14.7 MRD	Prior-β grain size 263 ± 8 µm and maximum texture intensity 3.5 MRD at 0.4 mm peening pitch	Finer β grains and weaker texture with decreasing peening pitch.
[86] Ti-6Al-4V + Interpass Rolling	OM; Neutron Diffraction; Contour method	Large columnar prior-β grains several mm in size; strong crystallographic texture	Fine equiaxed prior-β grains <100 µm after 75 kN rolling; texture largely eliminated	Strong grain refinement and texture weakening, with little change in residual stress.

^1^ OM, optical microscopy; SEM, scanning electron microscopy; BSE, backscattered electron imaging; EDS, energy-dispersive X-ray spectroscopy; XRD, X-ray diffraction; EBSD, electron backscatter diffraction; and TEM, transmission electron microscopy.

**Table 8:** Qualitative comparison of ex-situ and in-situ hybrid processing approaches in metal AM.

Parameters	Ex-Situ Processing	In-Situ Processing
Stage of intervention	After completion of the AM build.	During deposition, between layers, or within the fabrication sequence.
Material state targeted	Fully consolidated component after the AM thermal history, microstructure, and defect population are established.	Liquid melt pool, hot/viscoplastic material, or recently solidified layer before the final structure is fully established.
Typical processing scale	Primarily global/bulk treatment, although local surface or machining operations may also be used.	Primarily localized, layer-wise, interlayer, or region-specific treatment.
Defect control	Effective for post-build densification, surface correction, residual-stress modification, and removal or reshaping of established defects.	Can suppress, close, redistribute, or modify defects closer to the stage at which they form, before subsequent layers fully establish the structure.
Microstructure control	Can provide substantial bulk homogenization, recrystallization, phase control, texture modification, and microstructural reconstruction.	Can interrupt epitaxial grain inheritance, modify solidification, introduce stored strain, promote static or dynamic recrystallization.
Processing force/thermal exposure	Bulk deformation commonly requires larger loads; thermal treatments may require substantial machinery, energy and processing time.	Can exploit retained process heat and achieve useful local deformation with smaller loads; thermal interventions may be short and spatially targeted.
Geometry/accessibility	Limited by compatibility with furnaces, HIP vessels, forming dies, machining tools, and handling of the completed component.	Limited by access of secondary tooling or energy sources to the evolving deposit and by the architecture of the AM system.
Process sensitivity	Less dependent on individual layer timing; however, final response still depends on prior AM history and treatment conditions.	Strongly dependent on treatment timing, affected depth, layer sequence, remelting/reheating, and overlap with subsequent deposition.
Major advantage	Greater bulk uniformity and use of mature, established industrial processing routes, also provide bulk geometric forming capability.	Earlier and localized intervention, potential process-chain consolidation, reduced local forming load, and the ability to modify microstructure/defects before they are even established.
Major limitation	Additional handling, equipment, processing steps, energy, lead time, and potential loss of AM geometric freedom.	Integration complexity and spatially non-uniform response. Effectiveness depends strongly on process accessibility and subsequent thermal history.

## Data Availability

No new data were created or analyzed in this study. Data sharing is not applicable to this article.

## Abbreviations

The following abbreviations are used in this manuscript:

AFSD	Additive friction stir deposition
AM	Additive manufacturing
BCC	Body-centered cubic
BD	Build direction
BJ	Binder jetting
BSE	Backscattered electron imaging
CASE	Chemically assisted surface enhancement
CAVSFR	Compound arc and vibration shock forging–rolling
CNC	Computer numerical control
CO	Conventional or as-deposited condition
DED	Directed energy deposition
DRX	Dynamic recrystallization
EBSD	Electron backscatter diffraction
EDS	Energy-dispersive X-ray spectroscopy
FCC	Face-centered cubic
FGM	Functionally graded material
FSP	Friction stir processing
HAGB	High-angle grain boundary
HDPE	High-density polyethylene
HF	Hot-forged condition
HIP	Hot isostatic pressing
HR	Hot rolling
HSLA	High-strength low-alloy
HT	Heat treatment
HV	Vickers hardness
IQR	Interquartile range
KAM	Kernel average misorientation
K_IC_	Mode-I plane-strain fracture toughness
LAGB	Low-angle grain boundary
LP	Laser peening
ME	Material extrusion
MEA	Medium-entropy alloy
MF	Magnetic field
MRD	Multiples of a random distribution
MUD	Multiples of uniform distribution
NRR	Non-remelted region
OM	Optical microscopy
PBF	Powder bed fusion
PDAS	Primary dendrite arm spacing
PH	Precipitation hardening
PSPP	Process–structure–property–performance
Ra	Arithmetic average surface roughness
RR	Remelted region
RX	Recrystallization
SB	Sand-blasted baseline
SDAS	Secondary dendrite arm spacing
SEM	Scanning electron microscopy
SMA	Shape memory alloy
SMF	Static magnetic field
SP	Shot peening
SR-XRT	Synchrotron X-ray tomography
SRX	Static recrystallization
TCP	Topologically close-packed
TEA	Techno-economic analysis
TEM	Transmission electron microscopy
UIP	Ultrasonic impact peening
UTS	Ultimate tensile strength
WAAM	Wire arc additive manufacturing
WIP	Warm isostatic pressing
XCT	X-ray computed tomography
XRD	X-ray diffraction
XRT	X-ray tomography
YS	Yield strength
µCT	Micro-computed tomography

## Appendix A. Literature Review Methodology and Scope

Literature search for this study was conducted between January 2024 and May 2026 using Google Scholar, Scopus, and Web of Science as the primary multidisciplinary search platforms. Additional targeted searches were performed through ScienceDirect, Taylor & Francis Online, Springer Nature, and ResearchGate. With no formal lower cutoff, relevant studies were considered regardless of publication year. Studies were identified through a combination of keyword-based searching and citation chaining from relevant primary studies and review articles. Search terms combined metal AM process terminology, including “additive manufacturing,” “directed energy deposition,” “DED,” “wire DED,” “wire arc additive manufacturing,” “WAAM,” “powder DED,” “powder bed fusion,” “PBF,” “binder jetting,” and “material extrusion,” with hybrid-processing and secondary-process terms, including “hybrid,” “in-situ,” “interlayer,” “forging,” “hammering,” “peening,” “rolling,” “friction stir processing,” “ultrasonic,” “magnetic field,” “alloying,” “stirring,” “heat treatment,” “hot isostatic pressing,” “HIP,” “machining,” and “surface preparation.” Equivalent, legacy, and process-specific terminology were also used where relevant. For instance, searches for PBF included terms such as “laser melting,” “selective laser melting,” “DMLS,” and “laser sintering,” while searches for DED included terms such as “laser wire,” “hot wire,” “laser metal deposition,” and related process-specific nomenclature.

For the in-situ portion of the review, the objective was to include all qualifying studies identified through this search strategy. Studies were included when they involved metal AM, incorporated a distinguishable secondary-processing function during deposition, between deposited layers, or elsewhere within the fabrication sequence, and provided sufficient information to identify the AM process, material system, secondary process, and resulting effect on defects, microstructure, residual stress, phase evolution, surface or geometry, or mechanical properties. A secondary process did not necessarily require separate equipment; for instance, studies in which the AM heat source was deliberately reused in a separate reheating or heat-treatment step were included when that operation served a distinct secondary-processing function. In contrast, studies limited to conventional optimization of the primary AM parameters without a distinguishable secondary-processing function were not treated as hybrid processing. Review articles, conference abstracts lacking sufficient technical detail, editorials, purely conceptual studies, duplicate reports of essentially the same experiment, and studies lacking sufficient information for classification were excluded from the primary study dataset. Non-metal AM studies were excluded from the metal-AM dataset and quantitative distributions, except for targeted searches conducted specifically for the polymers and composites discussion.

The ex-situ literature was treated differently because conventional post-processing of AM materials represents a substantially broader and more mature body of work. Rather than attempting an exhaustive accumulation, representative studies were selected to span the major ex-situ processing routes, commonly used metal AM process families, and representative alloy systems. Consequently, it can be noted that the quantitative distributions presented in this review are restricted to the in-situ literature set rather than the representative ex-situ dataset. Particularly, for quantitative classification in in-situ routes, studies involving more than one secondary process were counted in each applicable secondary-process category, and studies investigating more than one alloy were counted once for each alloy investigated. Therefore, process or alloy-level totals may exceed the number of unique publications, with the counting basis identified in the corresponding figures and tables. Recrystallization (RX) responses were classified as static recrystallization (SRX), dynamic recrystallization (DRX), or no RX evidence. SRX was assigned when prior deformation introduced stored strain energy and recrystallization occurred subsequently during reheating, later deposition, or post-thermal exposure, including post-build heat treatment. DRX was assigned when recrystallization occurred during active hot deformation such that deformation and thermal activation overlapped. “No RX evidence” was assigned when deformation, globularization, or phase transformation was reported without clear evidence or an explicit claim of recrystallization. For transformation-assisted β recrystallization in titanium alloys, the response was classified as SRX or DRX according to the reported timing of recrystallization.
